# Systematic Structural Characterization of Chitooligosaccharides Enabled by Automated Glycan Assembly

**DOI:** 10.1002/chem.202005228

**Published:** 2021-01-07

**Authors:** Theodore Tyrikos‐Ergas, Vittorio Bordoni, Giulio Fittolani, Manishkumar A. Chaube, Andrea Grafmüller, Peter H. Seeberger, Martina Delbianco

**Affiliations:** ^1^ Department of Biomolecular Systems Max-Planck-Institute of Colloids and Interfaces Am Mühlenberg 1 14476 Potsdam Germany; ^2^ Department of Chemistry and Biochemistry Freie Universität Berlin Arnimallee 22 14195 Berlin Germany; ^3^ Department of Theory Max-Planck-Institute of Colloids and Interfaces Am Mühlenberg 1 14476 Potsdam Germany

**Keywords:** carbohydrates, chitin, chitosan, MD simulations, structure–property correlations

## Abstract

Chitin, a polymer composed of β(1–4)‐linked *N*‐acetyl‐glucosamine monomers, and its partially deacetylated analogue chitosan, are abundant biopolymers with outstanding mechanical as well as elastic properties. Their degradation products, chitooligosaccharides (COS), can trigger the innate immune response in humans and plants. Both material and biological properties are dependent on polymer length, acetylation, as well as the pH. Without well‐defined samples, a complete molecular description of these factors is still missing. Automated glycan assembly (AGA) enabled rapid access to synthetic well‐defined COS. Chitin‐cellulose hybrid oligomers were prepared as important tools for a systematic structural analysis. Intramolecular interactions, identified by molecular dynamics simulations and NMR analysis, underscore the importance of the chitosan amino group for the stabilization of specific geometries.

## Graphical abstract

**Automated glycan assembly** was employed to prepare a collection of chitooligosaccharides with full control over length, fraction, and pattern of acetylation. Systematic structural studies, based on molecular dynamics simulations and NMR experiments, revealed intramolecular interactions responsible for the stabilization of particular conformations.

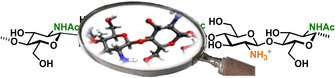

Polysaccharides are valuable biocompatible and recyclable materials. Chitin, a polymer composed of *N*‐acetylglucosamine repeating units, is the second most abundant polysaccharide in Nature, after cellulose. Chitin serves mainly structural roles in the exoskeleton of crustaceans, insects, and fungal cell wall.^[1]^ Its (partially) deacetylated counterpart—chitosan—is easily obtained via hydrolytic deacetylation of chitin and, due to its higher water solubility and easy functionalization, is used for industrial applications such as coating material, ingredient in cosmetics, and pharmaceutical excipient.^[2]^ Chitin and chitosan are commonly used to produce fibers, particles, and composites with exceptional biological and mechanical properties.^[3]^ Degree of polymerization (DP) and fraction of acetylation (FA) offer the opportunity to tune the stiffness, solubility, and transparency of the resulting materials.^[4]^

Chitin degradation produces chitooligosaccharides (COS). These short oligomers are known to trigger an innate immune response in humans^[5]^ and antifungal defense mechanisms in plants.^[6]^ The DP of COS is crucial for the biological response, as size‐dependent recognition was observed in plant chitin receptors as well as in toll‐like receptors (TLR2).^[7]^ It has been suggested that the acetylation pattern (AP) of COS modulates the biological activity^[8]^ and may explain the existence of sequence‐specific chitosan hydrolases in most organisms.^[9]^

A detailed molecular description of chitin, chitosan, and COS structure–function relation is missing, as most studies are performed with ill‐defined samples. Computationally, several all‐atom models have been applied to study the conformational space of COS, showing that DP, FA, AP as well as pH strongly affect the conformation and control aggregation.^[10]^ Coarse grained (CG) computational methods provide further insights on the COS interactions in solution, aiming for a description of chitin and chitosan polymers.^[11]^ However, due to the intrinsic CG approximation, chemical details are lost. In computational methods, the lack of standards to validate the theoretical models remains the major bottleneck, leading, in some cases, to contradictory theories.^[11b]^ Well‐defined samples with controlled DP, FA, and AP are therefore important targets to shine light on molecular conformations and interaction mechanisms.

COS commonly obtained by partial degradation of polymeric chitin and chitosan require extensive purification and typically exist as mixtures.^[6a]^ Chemical or enzymatic *N*‐(de)acetylation are common manipulations, but in most cases yield ill‐defined products with varying DP, FA, and AP.^[12]^ Sequence specificity or regioselectivity may be achieved enzymatically,^[13]^ but only few of the required enzymes are available. To date, no general method to produce all possible patterns exists.^[8]^ Alternatively, well‐defined but simple COS can be prepared by chemical synthesis using orthogonal protecting groups^[14]^ and glycosylation conditions.^[15]^ Conventional synthetic approaches are too laborious to prepare a large collection of well‐defined COS required for systematic structural studies. Solid phase based automated techniques offer the ideal solution for the quick production of large series of related compounds.^[16]^ Still, their scope is limited by problems with sequences that form rigid tertiary structures, such as cellulose and chitin oligomers.^[16b]^

Here, we report the automated glycan assembly (AGA) of a collection of hexasaccharides, including well‐defined COS as well as hybrid chitin‐cellulose oligomers. These unnatural analogues are designed to explore the importance of the amino groups in COS. Monosaccharide building blocks (BBs) are iteratively combined on a solid support, granting full‐control over length and acetylation pattern.^[17]^ The conformational space of the synthetic oligomers and recurrent intramolecular interactions are studied systematically using molecular dynamics (MD) simulations and NMR experiments.

Nine hexasaccharides were assembled by AGA employing four differentially protected thioglycoside or glycosyl phosphate monosaccharide BBs (Figure 1). A fluorenylmethoxycarbonyl (Fmoc) temporary protecting group masks the C(4) hydroxyl group that, upon cleavage, allows for chain elongation. Stereochemical control during glycosylation is ensured by C(2) anchimeric assistance by the protecting group.^[18]^ Two glucosamine BBs are designed for the introduction of either *N*‐acetyl (**N**) or free (**K**) glucosamine units. The amino group in **BB1** is equipped with the trichloroacetyl (TCA) group, whereas **BB2** bears a carboxybenzyl (Cbz) group. The glucose unit (**A**), required for the chitin‐cellulose oligomers, is installed using **BB3**. The desired sequence is assembled on solid support, following iterative cycles of glycosylation and deprotection (see Supporting Information). The protected oligomer is released from the solid support upon cleavage of the UV‐labile linker **4**.^[19]^ Hydrogenolysis removes all the benzyl ether (Bn) protecting groups and allows for the concomitant TCA reduction and Cbz cleavage, affording the desired COS with defined AP. The hybrid structures require basic hydrolysis of the benzoate esters (Bz), prior to hydrogenolysis.

**Figure 1:**
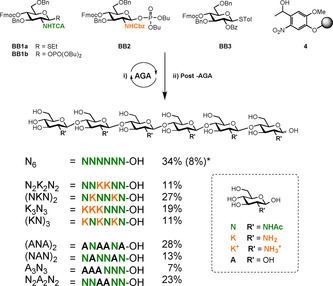
AGA of COS and cellulose–chitin hybrids. Isolated yields after AGA and post‐AGA (deprotection and purifications) are reported. * Yield obtained when AGA was performed by using **BB1a**.

AGA using thioglycoside **BB1a**, suffered from low yields and significant amounts of deletion sequences, particularly when multiple NN linkages had to be incorporated. This could be ascribed to the poor reactivity of the glucosamine acceptor^[20]^ and the potential formation of side‐products during activation of the thioglycoside.^[21]^ Glycosyl phosphate **BB1b** performed significantly better as indicated by an increase in yield of **N_6_** from 8 % to 34 %. Therefore, all COS were synthesized using glycosyl phosphate building blocks. Nine oligomers including the chitin oligomer **N_6_**, four COS with different acetylation degree and patterns, and four hybrid chitin‐cellulose analogues were assembled and were found to be highly water soluble (Figure 1).

The synthetic oligomers were modelled using MD simulations, employing a modified version of the GLYCAM06 carbohydrate force field.^[22]^ The partially deacetylated COS and the hybrid cellulose‐chitin oligomers were compared to the reference chitin oligomer **N_6_**. Amino substituted structures were simulated with neutral NH_2_ (**K**) as well as with protonated NH_3_^+^ (**K^+^**), as representative models of COS at different pH. All the modified analogues result in more flexible structures when compared to **N_6_**. Ramachandran plots are used to compare changes on the glycosidic bond torsion angles (Ψ, Φ) (Figure 2). No significant differences are observed for Φ, stabilized by the *exo*‐anomeric effect.^[23]^ The high population of Ψ at negative degrees (Figure 2 c, red circle) is related to the presence of the conventional O(5)⋅⋅⋅OH(3) hydrogen bond (Figure 2 b), which rigidifies the chitin structure (**N_6_**).^[16b]^ All modified oligomers show an increased population at positive Ψ (Figure 2 c, blue circles), albeit with different intensity. Major disruption of the conventional hydrogen bond is observed for the charged COS (e.g. **(NK^+^N)_2_**).

**Figure 2:**
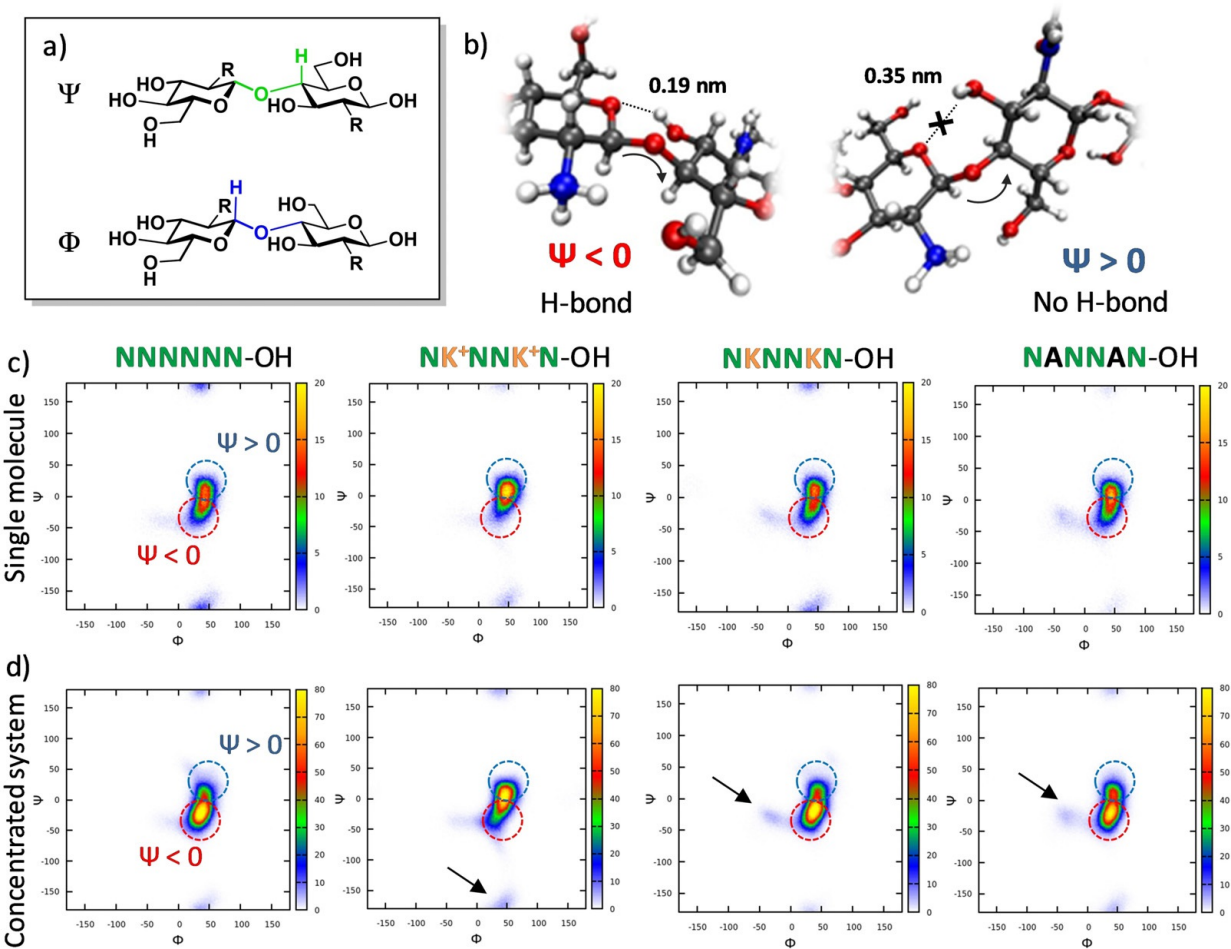
a) Definition of the glycosidic bond torsion angles (Ψ and Φ) and b) representative snapshots indicating the presence (Ψ**<**0) or absence (Ψ**>**0) of the conventional O(5)⋅⋅⋅OH(3) hydrogen bond. Analysis of the Ramachandran plots obtained by MD simulations c) for a single molecule and d) for a concentrated system with 25 molecules. Negative degrees of Ψ (red circles) indicate the presence of the conventional O(5)⋅⋅⋅OH(3) hydrogen bond, whereas the increased distance between these two residues is reflected by positive Ψ (blue circles). Deviation from the main conformations are highlighted with arrows.

Long MD atomistic simulations (1 μs production run) of concentrated experiments (25 molecules) were performed, to resemble a crowded environment. A remarkable increase in the population at negative Ψ is detected for **N_6_** and for all the modified uncharged structures (Figure 2 d, red circles). Stacking reduces the conformational freedom, favoring the formation of the O(5)⋅⋅⋅OH(3) hydrogen bond as well as inter‐molecular hydrogen bonds (Figure S13 in the Supporting Information). Deviations from the main population are observed for the modified compounds (Figure 2 d, arrows), suggesting a higher conformational freedom and a less regular packing than **N_6_**. The low aggregation tendency of the ionic COS **(NK^+^N)_2_** is confirmed by the similarity of the Ramachandran plots obtained for the single molecule and the concentrated experiments. In agreement with the computational model, powder XRD analysis shows sharp peaks for **N_6_**, indicating the tendency to pack with a regular architecture (Figure S6). All the modified compounds have amorphous XRD profiles, except from **A_3_N_3_** that shows a sharp peak at 21.3°, suggesting that the di‐block hybrid maintain the ability to pack in an ordered fashion (Figure S6). Interestingly, the XRD profile of **A_3_N_3_** does not resemble the XRD profiles of the natural analogs of cellulose (**A_6_**) or chitin (**N_6_**).

A closer look at the atomistic model shows a significant percentage of *tg* rotamers (orientation of the C6 side chain) for the charged COS (Figures 3 a and b).^[10a]^ MD suggests the formation of an intramolecular hydrogen bond between the NH_3_^+^(2) and the OH(6) of the following residue (R+1) (Figure 3 c). This happens regardless of the acetylation of the R+1 unit or the position of the two residues (**K^+^** and R+1) in the entire molecule. MD confirmed that this interaction is present also in the **KN** disaccharide (Figure S12). Therefore, to reduce the complexity of the hexamers that suffer from chemical shift degeneracy, NMR measurements were performed on the model dimer **5**. Selective 1D HOHAHA‐NMR experiments^[24]^ were performed to simplify the spectra even further (Figures S2–S5). ^3^ *J*_H5H6_ coupling constants were measured at different pH, as these values can be converted to rotamer percentage using empirical equations (Figure 3 d and Table S1).^[25]^ A small percentage of *tg* rotamers is detected at pH 4, when the amine is fully protonated. No *tg* rotamers are detected at basic pH (amine not protonated), in agreement with the predictions. Although NMR data indicate the existence of a small *tg* population at acidic pH, the calculated fraction of *tg* is significantly lower than the predicted value (Figure S12). Different simulation conditions using i) a different water model (tip3p), ii) N3 angle parameters derived in the context of GAG molecules,^[26]^ iii) increased ionic strength, and iv) reduced Lennard‐Jones interactions of the nitrogen atoms (consistent with the changes introduced in the GLYCAM_OSMO,r14_ force field) did not lead to major changes in the simulation results (Figure S12). This overrepresentation of the observed hydrogen bonds trend demonstrates the need of further optimization of the dihedral potentials, especially in the presence of ionic moieties (e.g., amines).

**Figure 3:**
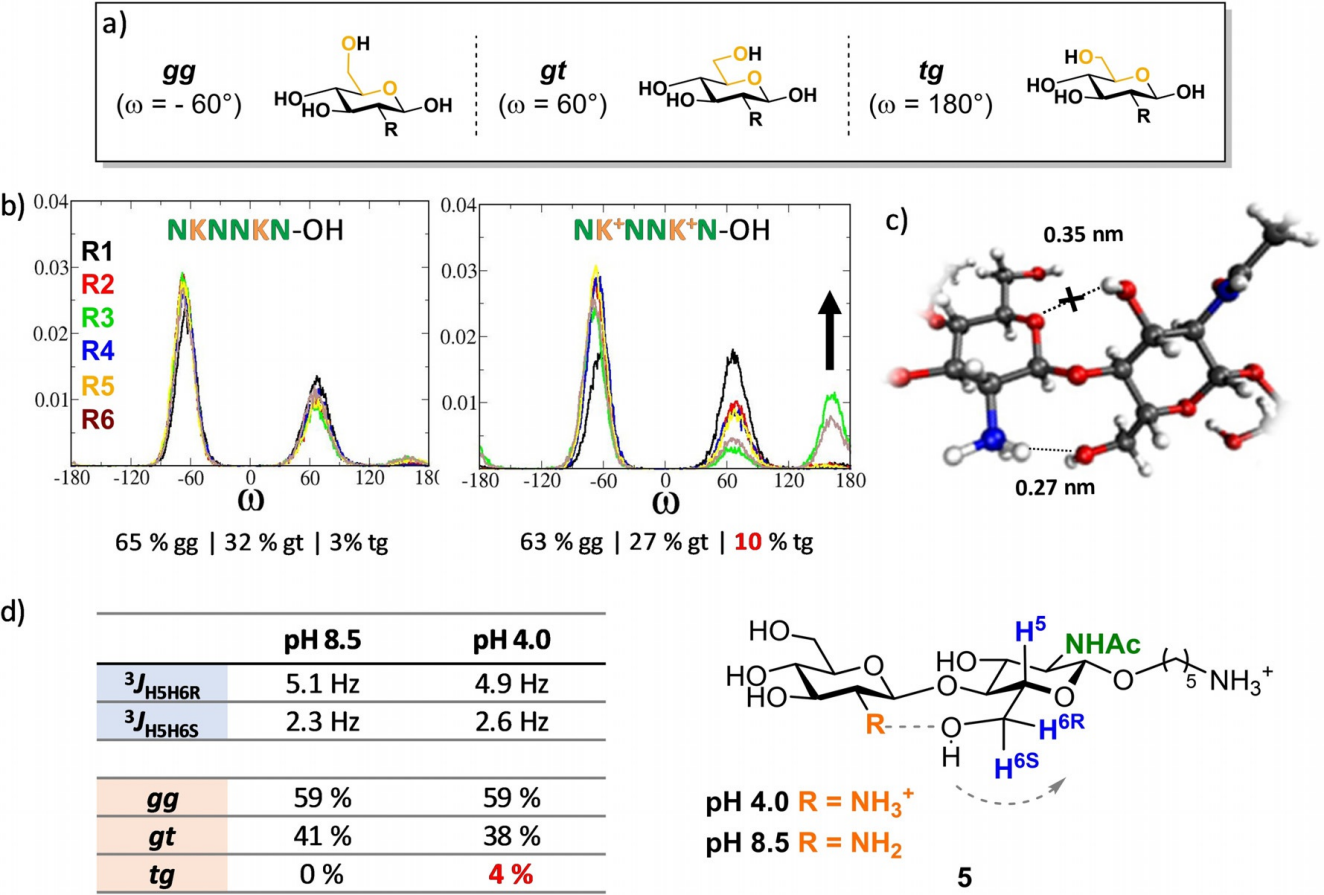
a) Definition of the ω torsion angle and b) ω distribution obtained by MD simulations for the NKNNKN analogue with different protonation. c) Representative snapshots indicating the presence of an intramolecular hydrogen bond between the NH_3_^+^(2) and the OH(6) of the following residue (R+1). d) NMR analysis of the ^3^ *J*_H5H6_ coupling constants measured for the model dimer **5** at a different pH.

In conclusion, AGA is a powerful tool to produce oligosaccharides that are essential for systematic structural analysis. Five COS were assembled with full‐control over length as well as acetylation degree and pattern. Four unnatural hybrid chitin‐cellulose oligomers were prepared to study the importance of the amino group in chitosan. Single molecule as well as concentrated MD simulations showed that all COS analogues have more conformational freedom than the fully *N*‐acetylated hexamer **N_6_**, resulting in amorphous aggregation upon drying. The hybrid compounds showed a similar conformational behavior as the neutral partially acetylated COS. Amine protonation results in intramolecular interactions, detected by NMR, that stabilize new geometries. This finding stresses the importance of the pattern of de‐acetylation of COS, because these interactions exist only in the presence of a deacetylated GlcN unit (**K^+^**). Therefore, knowing the position of the deacetylated residue in the polymer is essential for the description of the COS conformation. This observation is particularly relevant to clarify molecular mechanisms of chitosan‐protein interactions, as glycoside hydrolases binding is affected by the orientation of the C6 side chain.^[27]^

## Conflict of interest

The authors declare no conflict of interest.

## Supporting information

As a service to our authors and readers, this journal provides supporting information supplied by the authors. Such materials are peer reviewed and may be re‐organized for online delivery, but are not copy‐edited or typeset. Technical support issues arising from supporting information (other than missing files) should be addressed to the authors.

Supplementary
