# Supplementary material for: Systematic Structural Characterization of Chitooligosaccharides Enabled by Automated Glycan Assembly
Source: Chemistry. 2021 Jan 7;27(7):2321–5. doi: 10.1002/chem.202005228 (PMC7898498; doi:10.1002/chem.202005228)
Supplement: Supplementary file 1 — Supplementary [file CHEM-27-2321-s001.pdf]

Chemistry–A European Journal

Supporting Information

Systematic Structural Characterization of Chitooligosaccharides Enabled by Automated Glycan Assembly

Theodore Tyrikos-Ergas,[a, b] Vittorio Bordoni,[a] Giulio Fittolani,[a, b] Manishkumar A. Chaube,[a] Andrea Grafmüller,[c] Peter H. Seeberger,[a, b] and Martina Delbianco*[a]

Table of Contents

1. General Materials and Methods 2

2. Synthesis of Building Blocks 3

2.1 Synthesis of BB1 3

2.2 Synthesis of BB2 6

3. Automated Glycan Assembly 26

3.1 General Materials and Methods for AGA 26

3.2 Preparation of stock solutions 26

3.3 Modules for automated synthesis 26

3.4 Post-synthesizer manipulations 29

3.5 Oligosaccharides synthesis 31

3.5.1 Synthesis of NNNNNN-OH 31

3.5.2 Synthesis of ANAANA-OH 34

3.5.3 Synthesis of AAANNN-OH 37

3.5.4 Synthesis of NANNAN-OH 40

3.5.5 Synthesis of NNAANN-OH 43

3.5.6 Synthesis of KKKNNN-OH 46

3.5.7 Synthesis of KNKNKN-OH 49

3.5.8 Synthesis of NKNNKN-OH 52

3.5.9 Synthesis of NNKKNN-OH 55

4. Synthesis and NMR analysis of dimer 5 (KN-NH_2) 58

5. XRD Analysis 67

6. Molecular Dynamics Simulations 68

7. References 90

1. General Materials and Methods

All chemicals used were reagent grade and used as supplied unless otherwise noted. The automated syntheses were performed on a home-built synthesizer developed at the Max Planck Institute of Colloids and Interfaces. Analytical thin-layer chromatography (TLC) was performed on Merck silica gel 60 F254 plates (0.25 mm). Compounds were visualized by UV irradiation or dipping the plate in a *p*-anisaldehyde (PAA) solution. Flash column chromatography was carried out by using forced flow of the indicated solvent on Fluka Kieselgel 60 M (0.04 – 0.063 mm). Analysis and purification by normal and reverse phase HPLC was performed by using an Agilent 1200 series. Products were lyophilized using a Christ Alpha 2-4 LD plus freeze dryer. ^{1}H, ^{13}C and HSQC NMR spectra were recorded on a Varian 400-MR (400 MHz), Varian 600-MR (600 MHz), or Bruker Biospin AVANCE700 (700 MHz) spectrometer. Spectra were recorded in $CDCl_3$ by using the solvent residual peak chemical shift as the internal standard ($CDCl_3$: 7.26 ppm ^{1}H, 77.0 ppm ^{13}C) or in D_2O using the solvent as the internal standard in ^{1}H NMR (D_2O: 4.79 ppm ^{1}H) or in CD_3OD using the solvent as the internal standard in ^{1}H NMR (CD_3OD: 4.87 ppm ^{1}H, 49.0 ppm ^{13}C). High resolution mass spectra were obtained using a 6210 ESI-TOF mass spectrometer (Agilent) and a MALDI-TOF autoflex™ (Bruker). MALDI and ESI mass spectra were run on IonSpec Ultima instruments. IR spectra were recorded on a Perkin-Elmer 1600 FTIR spectrometer. Optical rotations were measured by using a Perkin-Elmer 241 and Unipol L1000 polarimeter. For XRD measurements, a Bruker D8 Advanced X-ray diffractometer with Cu Kα radiation was used.

2. Synthesis of Building Blocks

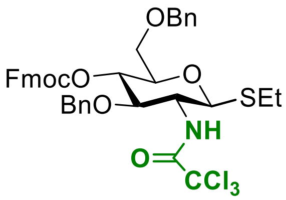

BB1a

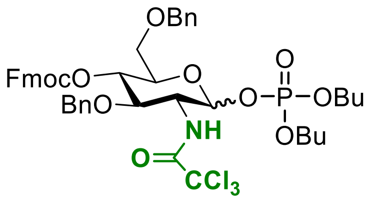

BB1b

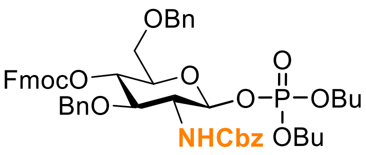

BB2

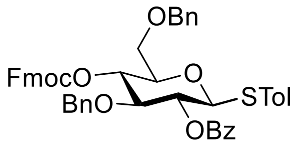

BB3

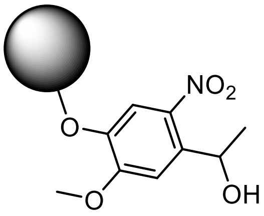

4

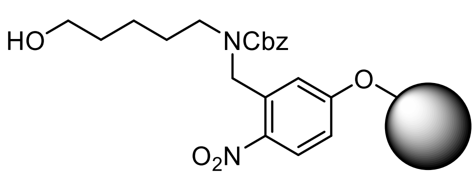

6

BB1a and **BB3** were obtained from commercial sources, while photocleavable linkers **4** and **6** were prepared according to literature.[1]

2.1 Synthesis of BB1

Dibutylphosphoryloxy 3,6-di-*O*-benzyl-4-*O*-fluorenylmethoxycarbonyl-2-deoxy-2-trichloroacetamido-α/β-D-glucopyranoside, BB1b

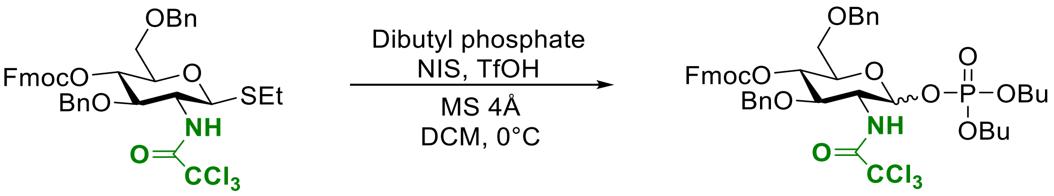

BB1a **BB1b**

Compound **BB1a** (2.00 g, 2.59 mmol) was coevaporated with toluene and dried in high vacuum overnight, after which time it was dissolved in anhydrous DCM (57.6 mL) under Argon atmosphere. Dibutyl phosphate (1.03 mL, 5.18 mmol) and 4Å molecular sieves were added to the solution. The suspension was stirred for 1 h and then cooled to 0°C. NIS (0.71 g, 3.16 mmol) and TfOH (16.3 µL, 0.18 mmol) were added. After 30 min, the reaction was carefully quenched with NEt_3 (36.2 µL, 26.0 µmol), diluted with DCM, allowed to room temperature and filtered. The mixture was washed once with 10% aqueous $Na_2S_2O_3$ and once with water. The organic phase was dried over $MgSO_4$, filtered and concentrated. The crude mixture was purified by column chromatography (EtOAc: Hexane = 1:2, R_f = 0.25) to obtain compound **BB1b** as a sticky colorless solid (2.02 g, 85%; α/β ~1:8).

Analytical data for the β-anomer: ^{1}H NMR (400 MHz, $CDCl_3$) δ 7.76 (t, *J* = 6.8 Hz, 2H), 7.56 (dd, *J* = 13.4, 7.5 Hz, 2H), 7.40 (td, *J* = 7.3, 4.9 Hz, 2H), 7.32 – 7.28 (m, 6H), 7.23 – 7.18 (m, 6H), 6.83 (d, *J* = 8.7 Hz, 1H, NH-β), 5.74 (dd, *J* = 6.1, 3.3 Hz, 1H, β-H1), 5.11 (t, *J* = 9.7 Hz, 1H), 4.61 (t, *J* = 11.6 Hz, 2H), 4.52 (d, *J* = 11.8 Hz, 1H), 4.48 (d, *J* = 11.8 Hz, 1H), 4.36 – 4.30 (m, 3H), 4.21 (dt, *J* = 10.2, 4.0 Hz, 1H), 4.14 – 3.94 (m, 6H), 3.62 – 3.60 (m, 2H), 1.64 – 1.56 (m, 4H), 1.39 – 1.30 (m, 4H), 0.93 – 0.88 (m, 6H); ^{13}C NMR (101 MHz, $CDCl_3$) δ 162.0, 154.1, 143.2, 143.1, 141.43, 141.40, 137.6, 137.1, 128.6, 128.4, 128.09, 128.03, 127.9, 127.8, 127.32, 127.31, 125.1, 125.0, 120.2, 95.5, 95.4, 92.1, 76.2, 74.7, 74.1, 73.7, 71.6, 70.2, 68.6, 68.46, 68.42, 68.40, 68.3, 54.3, 54.2, 46.7, 32.33, 32.31, 32.26, 32.24, 18.7, 13.74, 13.71, 13.6; ^{31}P NMR (162 MHz, $CDCl_3$) δ -2.59; (ESI-HRMS) m/z 940.2151 $[M+Na]^+$ ($C_{45}H_{51}Cl_3NO_{11}PNa$ requires 940.2158).

^{1}H NMR of BB1b (400 MHz, $CDCl_3$)

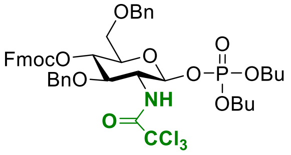

BB1b

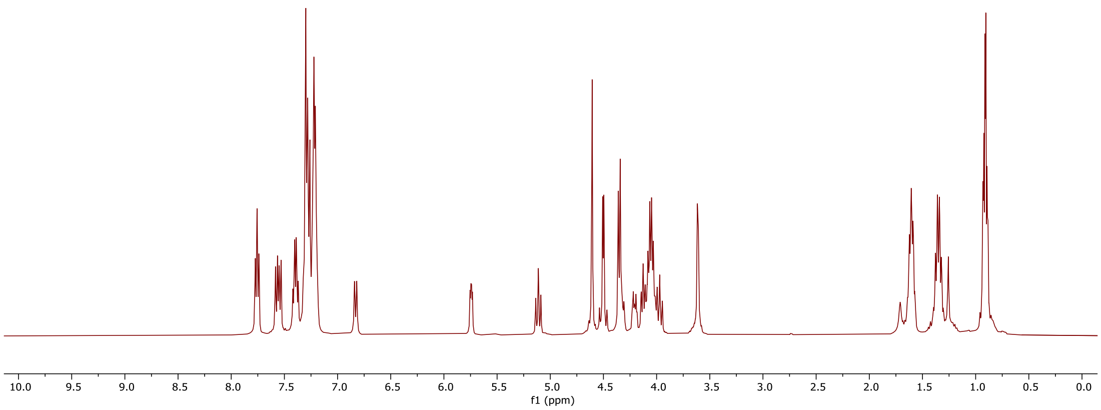

^{13}C NMR of BB1b (101 MHz, $CDCl_3$)

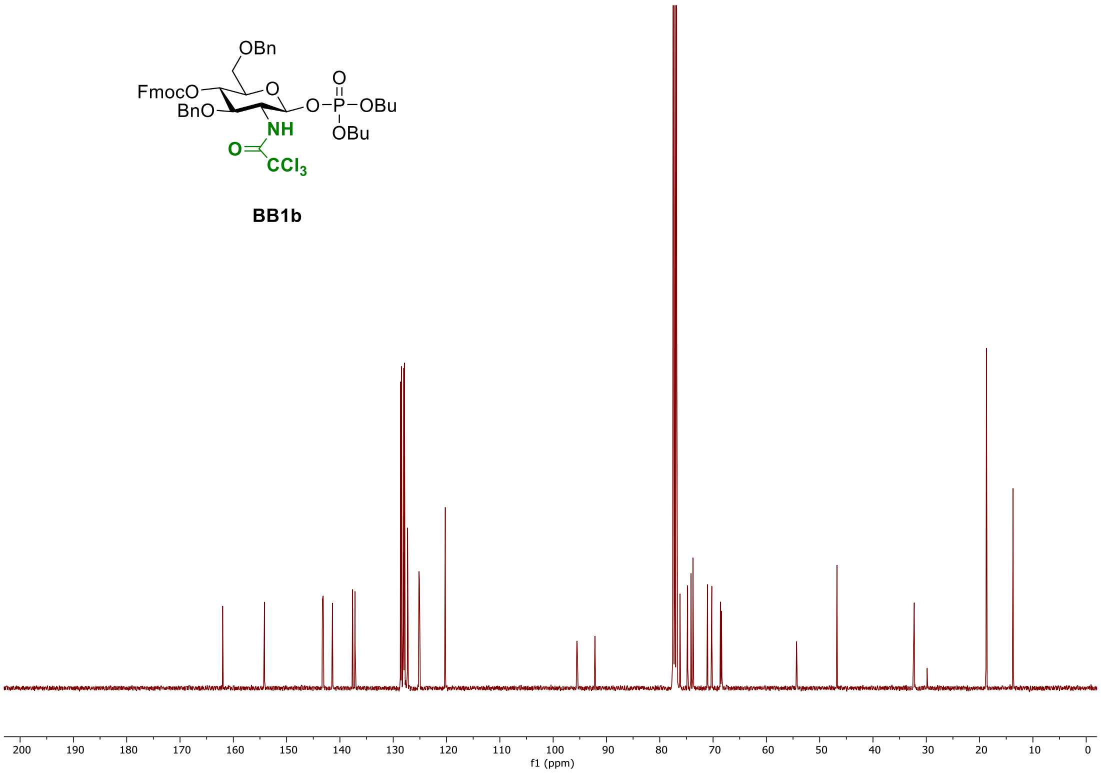

^{31}P NMR of BB1b (162 MHz, $CDCl_3$)

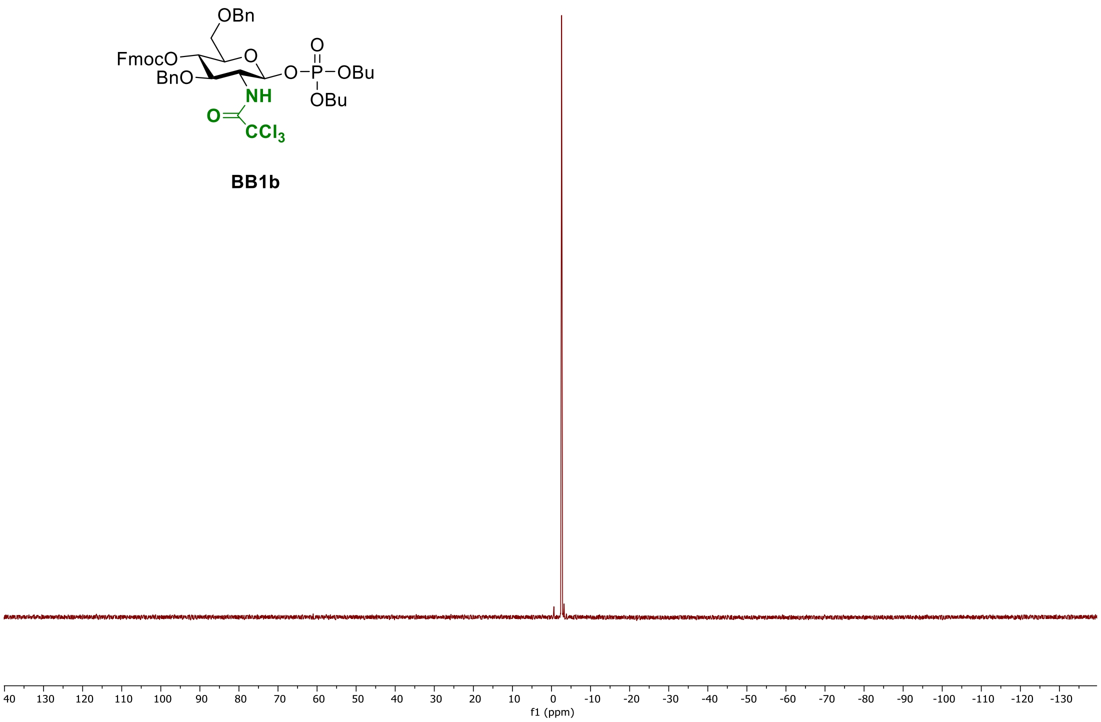

HSQC NMR of BB1 ($CDCl_3$)

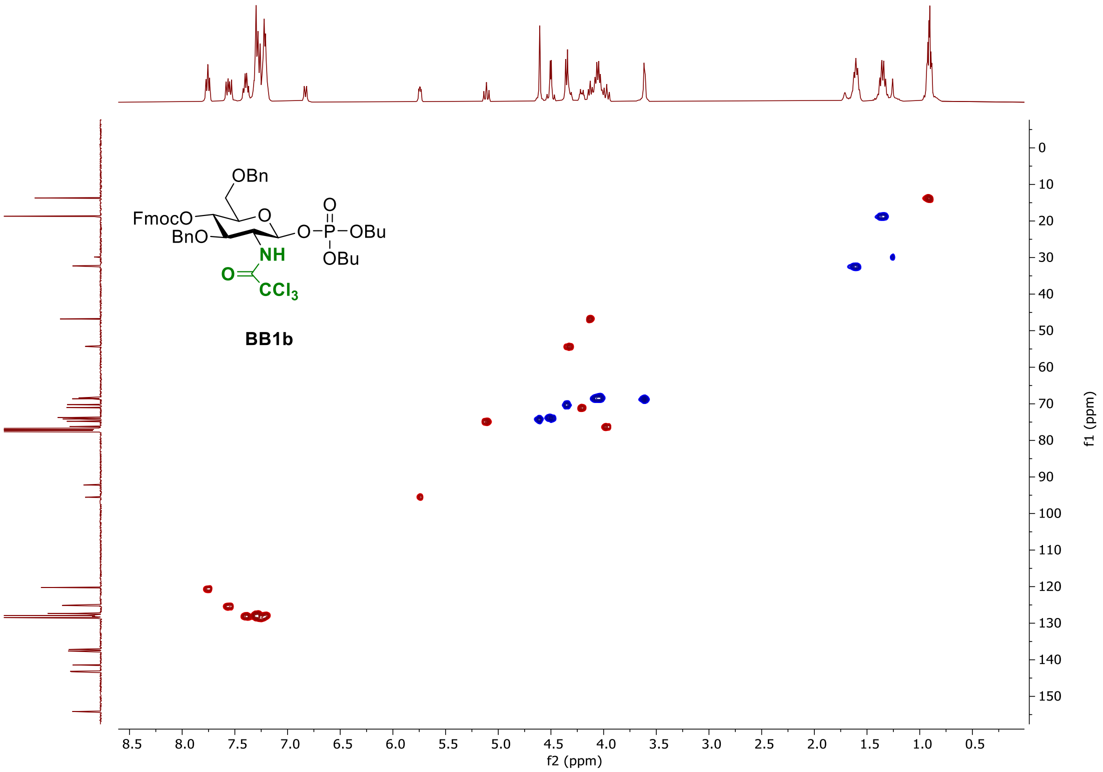

2.2 Synthesis of BB2

1,3,4,6-tetra-*O*-acetyl-2-deoxy-2-(benzyloxycarbonyl)amino-α/β-D-glucopyranoside, S1

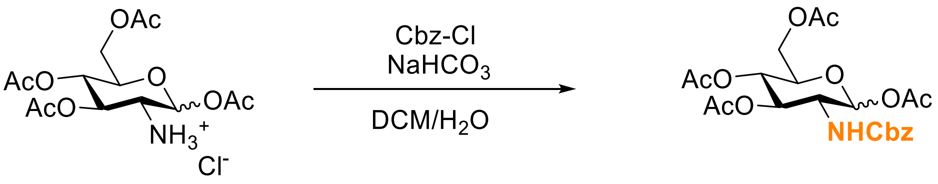

Glucosamine **S0** (10.0 g, 26.0 mmol) was dissolved in DCM (100 mL) and water (200 mL) and $NaHCO_3$ (5.5 g, 65 mmol) was added to reach pH 9. CbzCl (5.6 mL, 39 mmol) was added and the reaction was stirred at room temperature overnight, after which time the crude mixture was diluted with DCM. The solution was portioned between organic and aqueous layers and the aqueous layer was extracted with DCM (2 x 100 mL). The combined organic phases were washed with H_2O and brine. The organic phase was dried over Na_2SO_4, filtered and concentrated under reduced pressure. The crude product was purified by recrystallization from EtOAc – hexane (EtOAc: Hexane = 1:1, R_f = 0.4) to yield **S1** as white solid (12.4 g, 99%).

^{1}H NMR (400 MHz, $CDCl_3$) δ 7.40 – 7.26 (m, 5H), 5.67 (d, *J* = 8.8 Hz, 1H), 5.30 – 5.01 (m, 4H), 4.83 (d, *J* = 9.3 Hz, 1H), 4.28 (dd, *J* = 12.5, 4.6 Hz, 1H), 4.10 (dd, *J* = 12.6, 2.2 Hz, 1H), 3.94 (q, *J* = 9.5 Hz, 1H), 3.79 (ddd, *J* = 9.7, 4.5, 2.0 Hz, 1H), 2.08 (s, 3H), 2.03 (s, 6H), 1.94 (s, 3H).; ^{13}C NMR (101 MHz, $CDCl_3$) δ 171.04, 170.79,

169.54, 169.50, 155.92, 136.37, 128.58, 128.28, 128.06, 92.54, 72.68, 72.43, 68.06, 66.94, 61.71, 54.78, 20.82, 20.79, 20.64, 20.57; $[\alpha]_D^{20}$ +7.71 (*c* 1 g/100 mL, CH_2Cl_2); IR (neat) ν_{max} = 3347.66, 2963.68, 1747.74, 1533.08, 1368.59, 1215.98, 1075.75, 1036.24 cm^{-1}; (ESI-HRMS) m/z 504.1484 $[M+Na]^+$ ($C_{22}H_{27}NO_{11}Na$ requires 504.1482).

^{1}H NMR of S1 (400 MHz, CDCl$_3$)

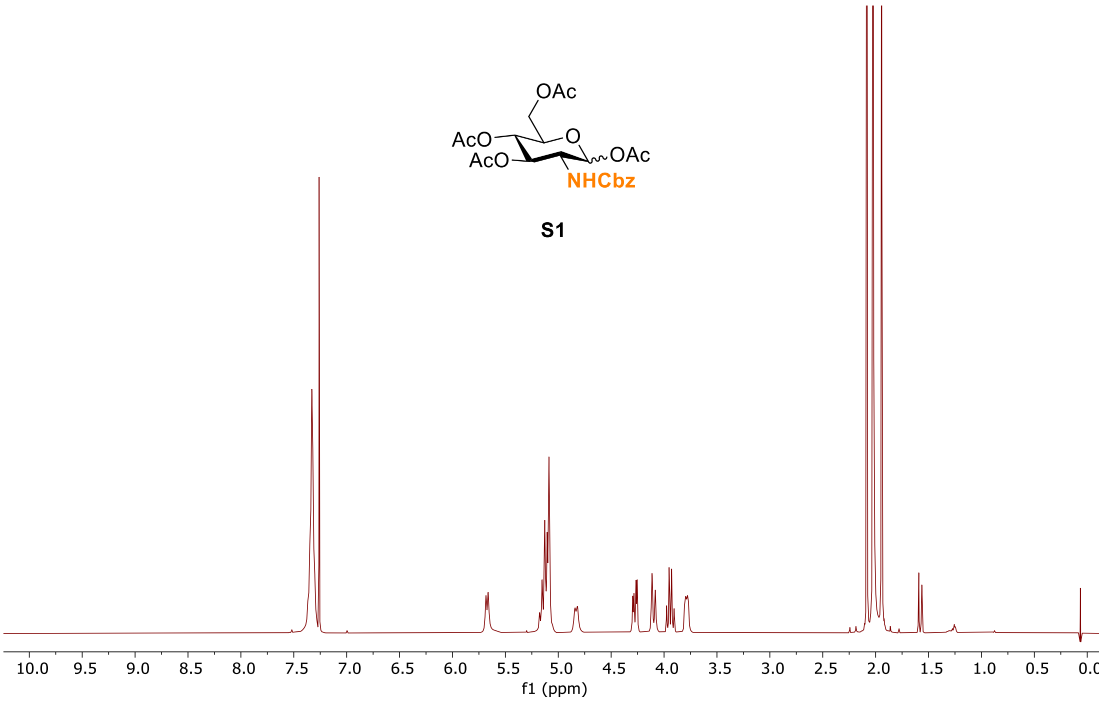

^{13}C NMR of S1 (101 MHz, CDCl$_3$)

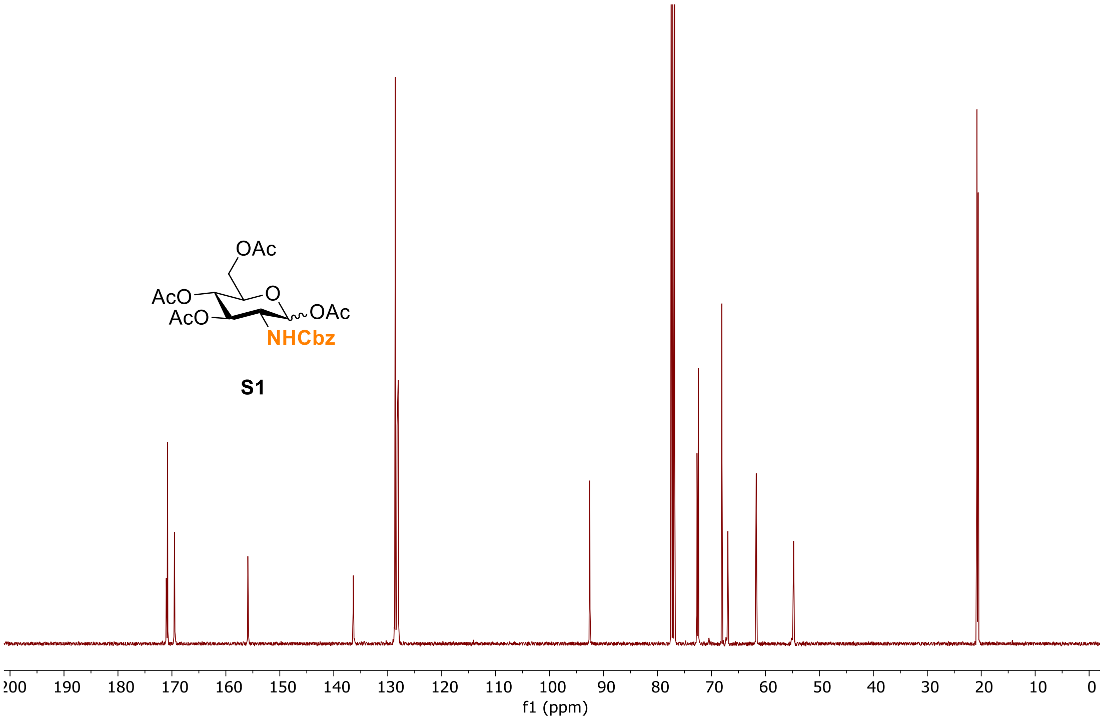

HSQC NMR of S1 ($CDCl_3$)

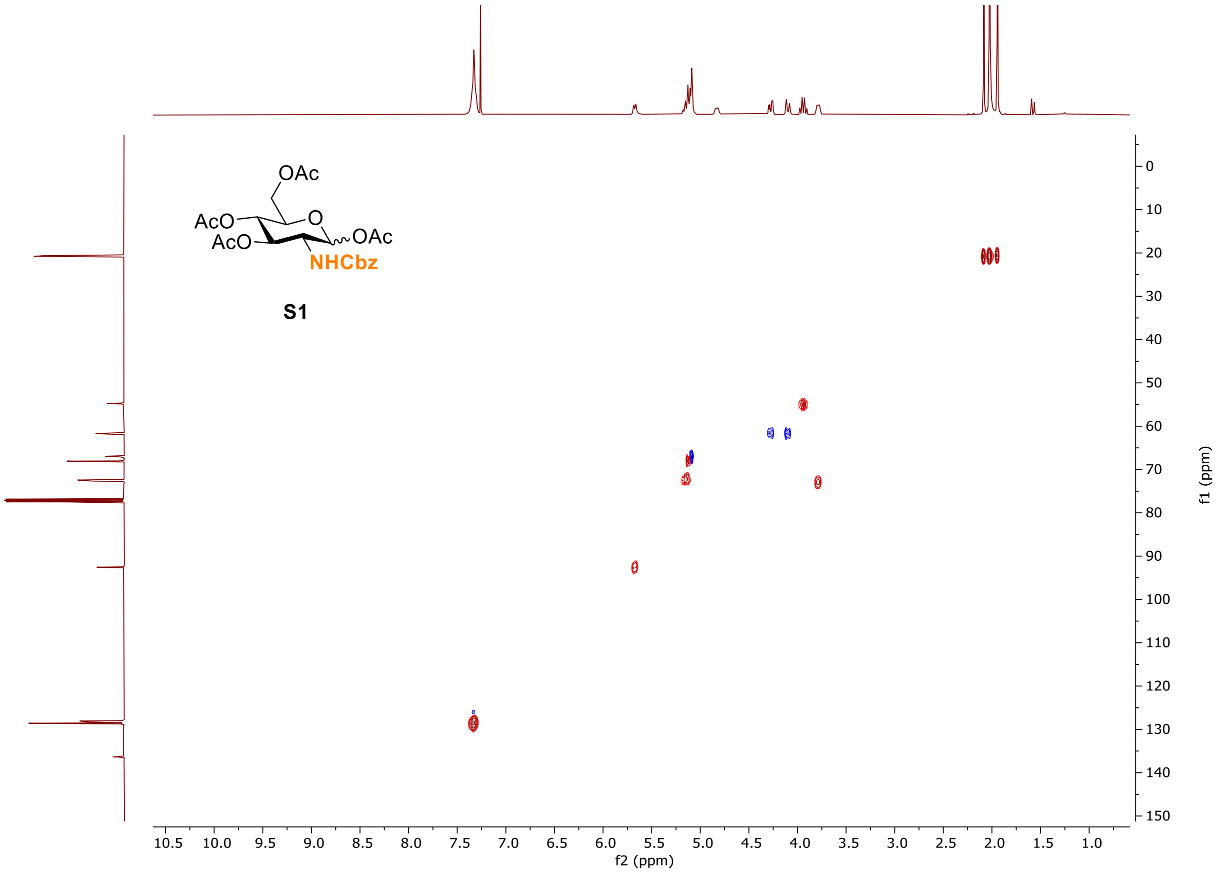

Ethyl 2,4,6-tri-*O*-acetyl-2-deoxy-2-(benzyloxycarbonyl)amino-1-thio-β-D-glucopyranoside, S2

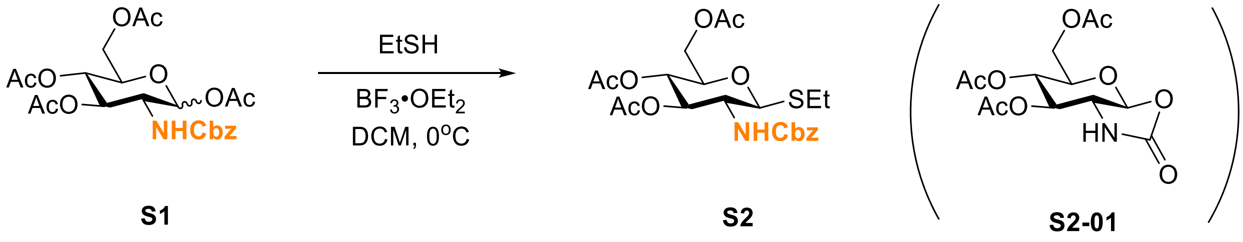

$BF_3{\cdot}EtO_2$ complex (8.0 mL, 65 mmol) was added dropwise to a stirred solution of **S1** (12.4 g, 26 mmol) and EtSH (4.8 mL, 65 mmol) in DCM (150 mL) at 0°C under Argon atmosphere. After 2 h at 0°C the reaction was quenched with $NaHCO_3$ sat. aq. solution. The mixture was diluted with DCM and washed three times with $NaHCO_3$ sat. aq. solution and one time with brine. The organic phase was dried over Na_2SO_4 and concentrated under reduced pressure. The crude mixture was purified by column chromatography

(EtOAc: Hexane = 2:3, R_f = 0.3) to yield **S2** as white solid (6.0 g, 48%). The yield of this reaction remained relatively low due to the significant formation of a side product identified as the bicyclic product **S2-01** (4.0 g, 46%, EtOAc: Hexane = 1:1, R_f = 0.2).

Analytical data for **S2**: ^{1}H NMR (400 MHz, $CDCl_3$) δ 7.43 – 7.29 (m, 5H), 5.37 – 4.99 (m, 4H), 4.83 (d, *J* = 9.4 Hz, 1H), 4.61 (d, *J* = 10.4 Hz, 1H), 4.24 (dd, *J* = 12.3, 5.1 Hz, 1H), 4.12 (dd, *J* = 12.3, 2.3 Hz, 1H), 3.76 (q, *J* = 10.0 Hz, 1H), 3.71 – 3.58 (m, 1H), 2.77 – 2.67 (m, 2H), 2.07 (s, 3H), 2.02 (s, 3H), 1.93 (s, 3H), 1.26 (t, *J* = 7.4 Hz, 3H);^{13}C NMR (101 MHz, $CDCl_3$) δ 170.95, 170.89, 169.58, 155.78, 136.37, 128.63, 128.32, 128.14, 84.84, 75.97, 73.62, 68.56, 67.15, 62.45, 55.21, 24.57, 20.93, 20.78, 20.70, 14.91; $[\alpha]_D^{20}$ -19.61 (*c* g/100 mL, CH_2Cl_2); IR (neat) ν_{max} = 3335.21, 1741.49, 1695.73, 1541.94, 1231.99, 1021.70 cm^{-1}; (ESI-HRMS) m/z 506.1460 $[M+Na]^+$ ($C_{22}H_{22}NO_9SNa$ requires 506.1461).

Analytical data for **S2-01**: ^{1}H NMR (400 MHz, $CDCl_3$) δ 6.41 (s, 1H), 5.95 (d, *J* = 7.0 Hz, 1H), 5.01 (ddd, *J* = 8.9, 5.1, 0.8 Hz, 1H), 4.96 (dd, *J* = 5.2, 3.9 Hz, 1H), 4.29 (dd, *J* = 12.3, 5.3 Hz, 1H), 4.18 (dd, *J* = 12.3, 2.7 Hz, 1H), 4.13 – 4.05 (m, 1H), 3.92 (dd, *J* = 7.0, 3.9 Hz, 1H), 2.10 (s, 3H), 2.08 (s, 3H), 2.07 (s, 3H); ^{13}C NMR (101 MHz, $CDCl_3$) δ 170.73, 170.39, 169.67, 156.80, 96.43, 72.01, 69.18, 66.51, 62.45, 52.97, 20.88, 20.82, 20.80; $[\alpha]_D^{20}$ +15.32 (*c* g/100 mL, CH_2Cl_2); IR (neat) ν_{max} = 3361.62, 2955.24, 1743.15, 1371.24, 1227.23, 1040.82 cm^{-1}; (ESI-HRMS) m/z 354.0832 $[M+Na]^+$ ($C_{13}H_{17}NO_9Na$ requires 354.0801).

^{1}H NMR of S2 (400 MHz, $CDCl_3$)

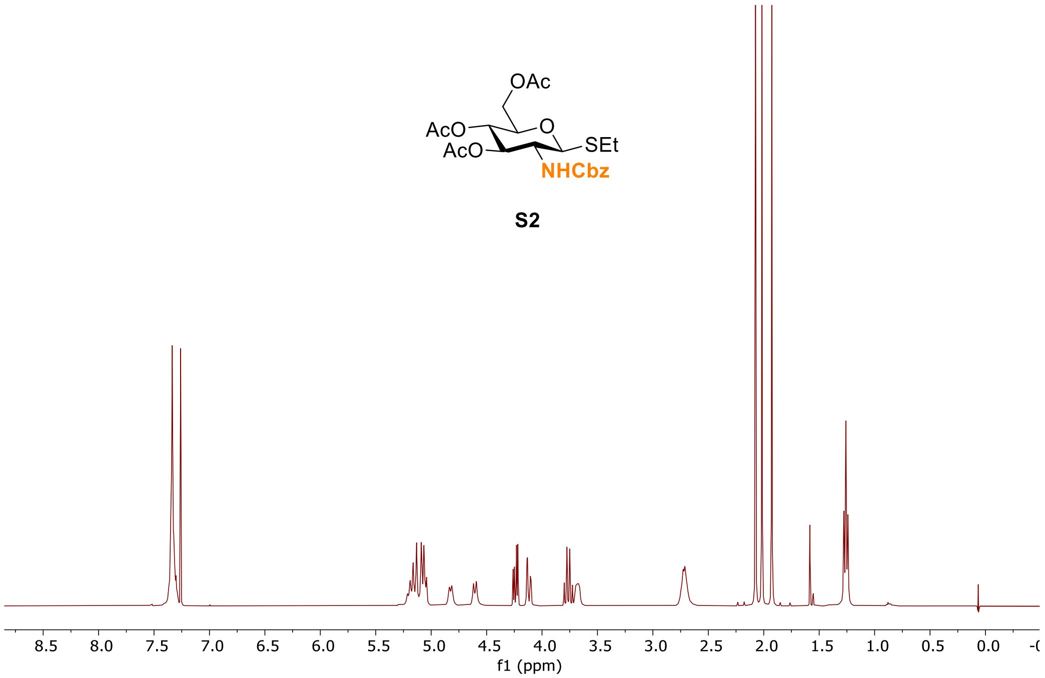

^{13}C NMR of S2 (101 MHz, $CDCl_3$)

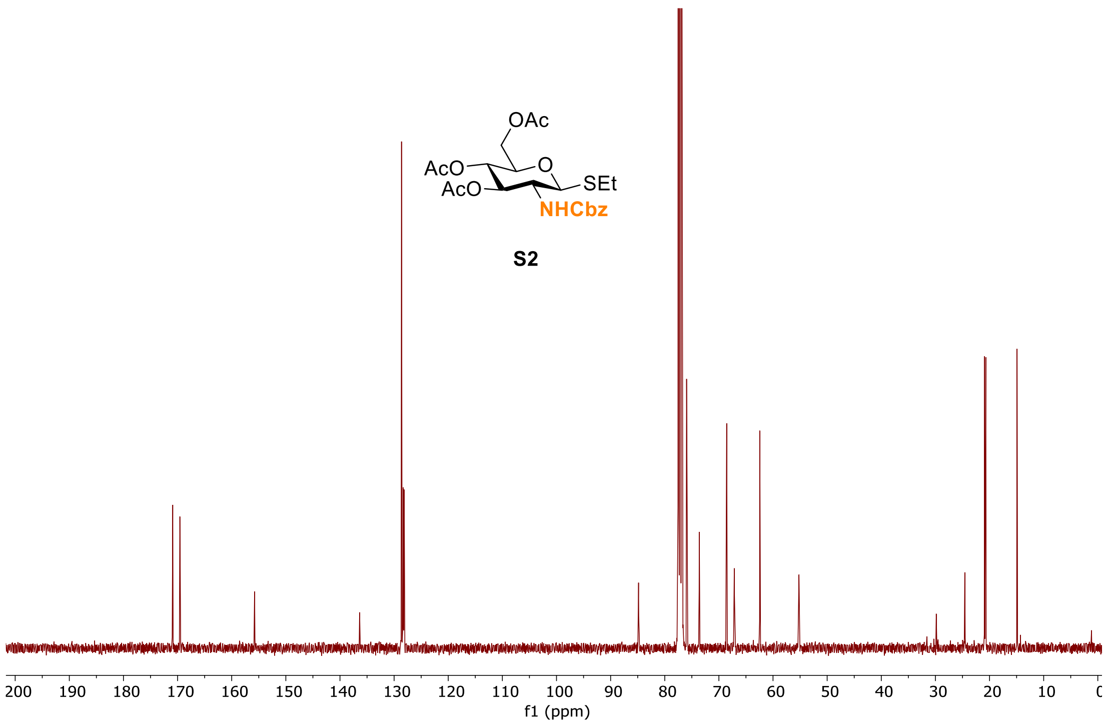

HSQC NMR of S2 ($CDCl_3$)

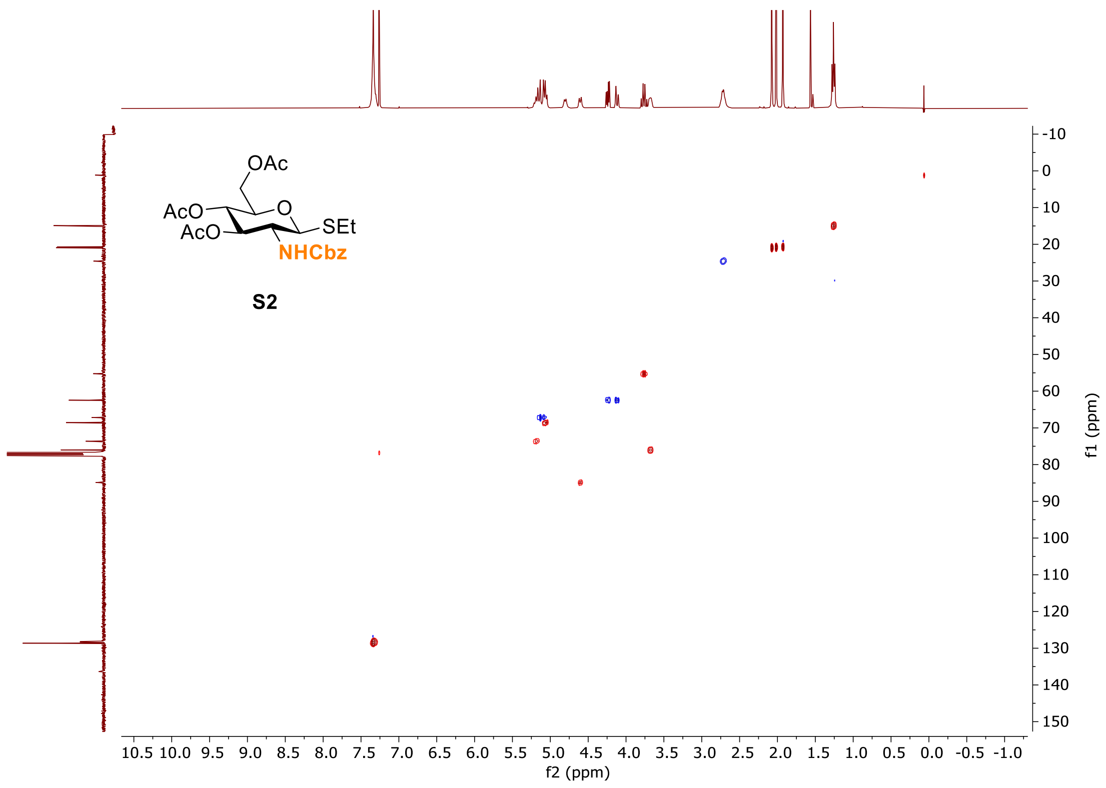

^{1}H NMR of S2-01 (400 MHz, $CDCl_3$)

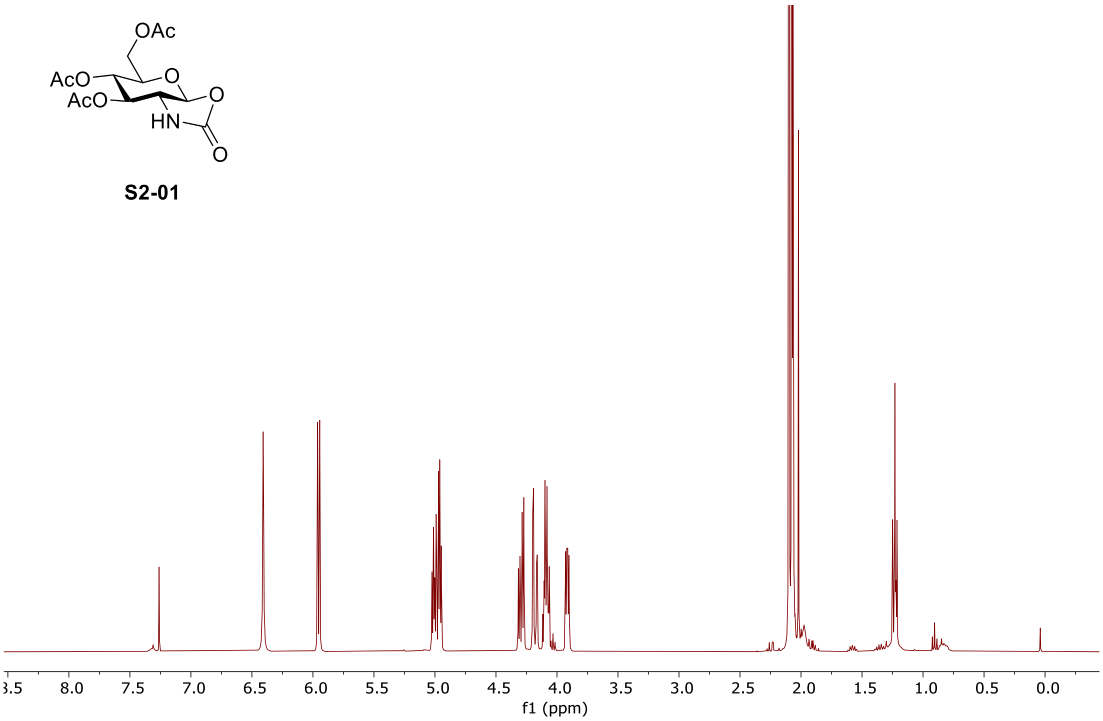

^{13}C NMR of S2-01 (101 MHz, $CDCl_3$)

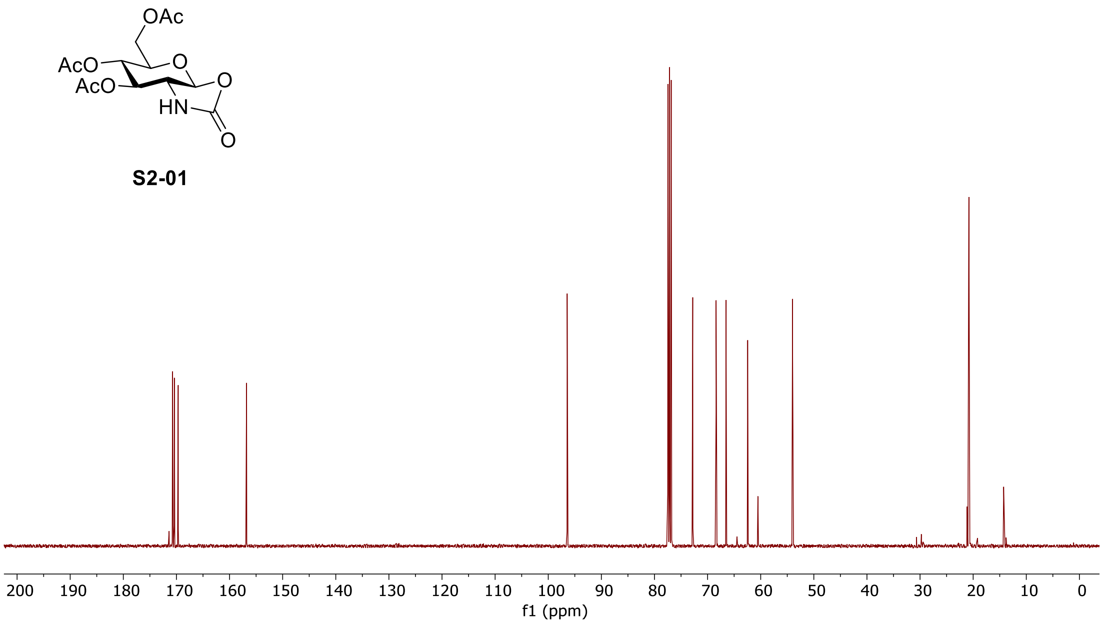

HSQC NMR of S2-01 ($CDCl_3$)

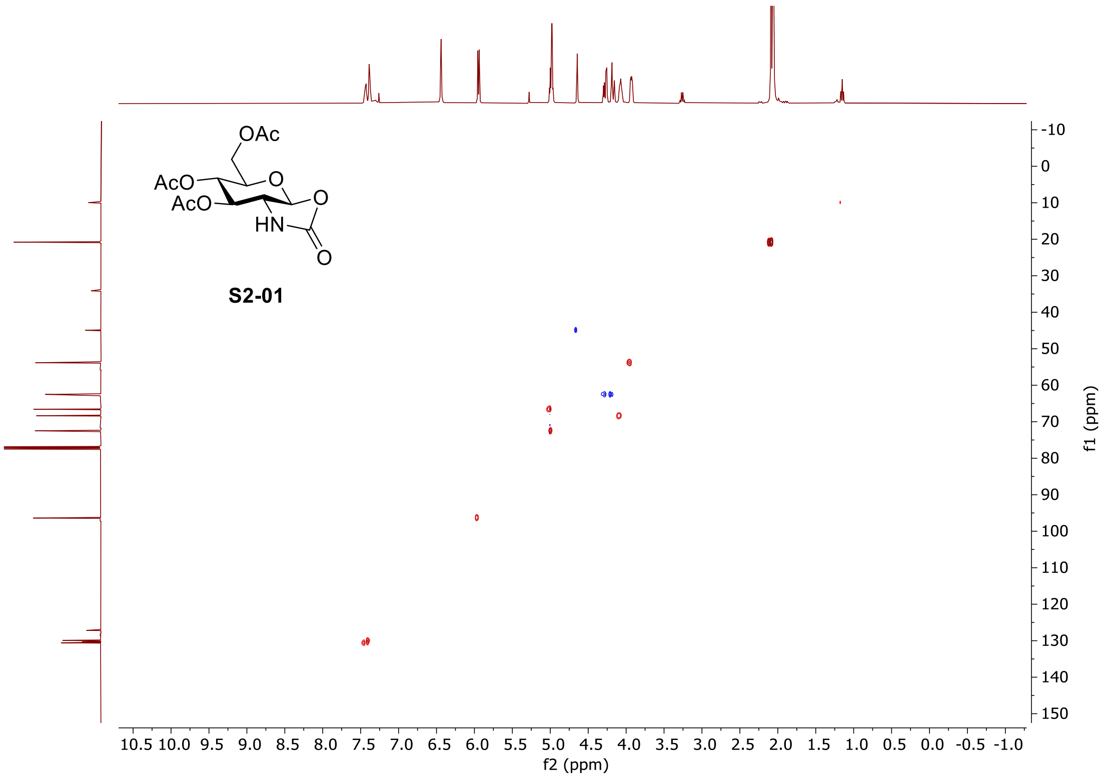

Ethyl 2-deoxy-2-(benzyloxycarbonyl)amino-1-thio-β-D-glucopyranoside, S3

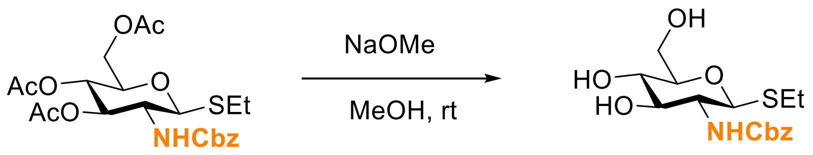

S2 **S3**

Ethyl 2,4,6-tri-*O*-acetyl-2-(benzyloxycarbonyl)amino-2-deoxy-1-thio-β-D-glucopyranoside **S2** (6.0 g, 12 mmol) was dissolved in a mixture of anhydrous MeOH-DCM (12.6 mL, 5:1 mL). A 0.5 M solution of MeONa in MeOH (7.20 mL, 3.60 mmol) was added. The mixture was stirred at room temperature for 1 h. The reaction was neutralized with Amberlite IR-120 (H^+ form), filtered, and concentrated under reduced pressure to yield **S3** as a white solid. The product was used in the next step without any further purification assuming quantitative conversion (4.4 g, quantitative, MeOH: DCM = 1:14, R_f = 0.2).
^{1}H NMR (400 MHz, MeOD) δ 7.34 – 7.24 (m, 5H), 5.09 (d, *J* = 12.4 Hz, 1H), 5.03 (d, *J* = 12.6 Hz, 1H), 4.46 (d, *J* = 9.9 Hz, 1H), 3.83 (dd, *J* = 12.0, 2.1 Hz, 1H), 3.62 (dd, *J* = 12.0, 5.7 Hz, 1H), 3.43 – 3.36 (m, 2H), 3.31 – 3.20 (m, 2H), 2.73 – 2.62 (m, 2H), 1.20 (t, *J* = 7.4 Hz, 3H); ^{13}C NMR (101 MHz, MeOD) δ 158.83, 138.38, 129.37, 128.85, 128.69, 85.98, 82.06, 77.35, 71.99, 67.39, 62.96, 58.10, 24.90, 15.17; $[\alpha]_D^{20}$ -26.25 (*c* g/100

mL, MeOH); IR (neat) ν_{max} = 3297.80, 1688.66, 1554.70, 1311.47, 1073.59 cm^{-1}; (ESI-HRMS) m/z 380.1142 $[M+Na]^+$ ($C_{16}H_{23}NO_6SNa$ requires 380.1144).

^{1}H NMR of S3 (400 MHz, MeOD)

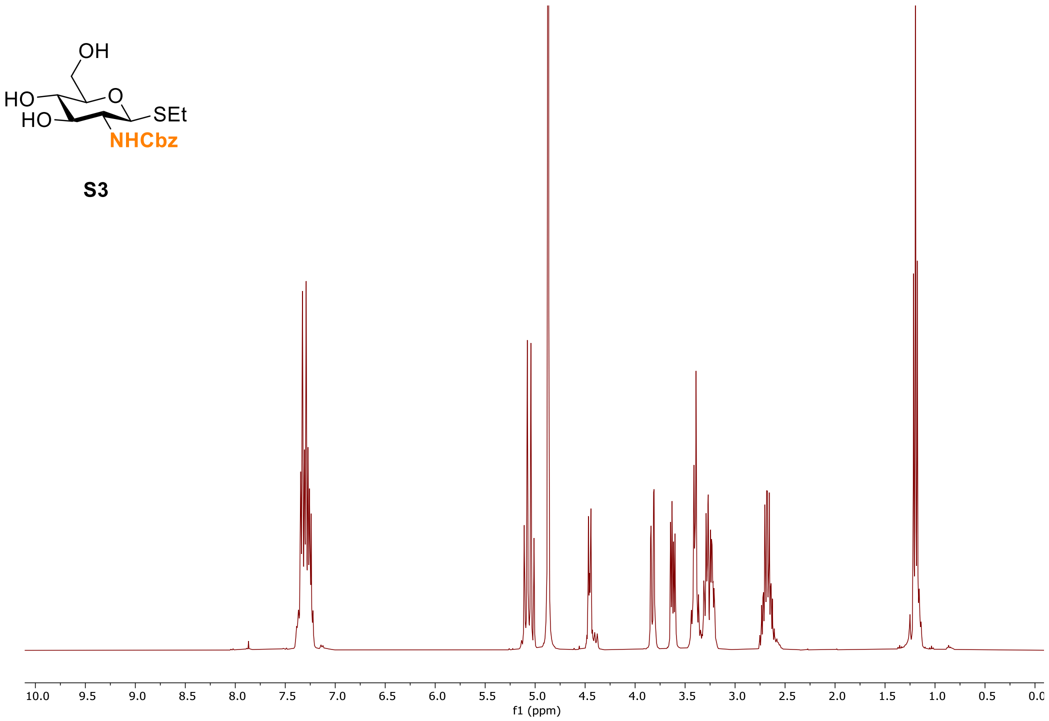

^{13}C NMR of S3 (101 MHz, MeOD)

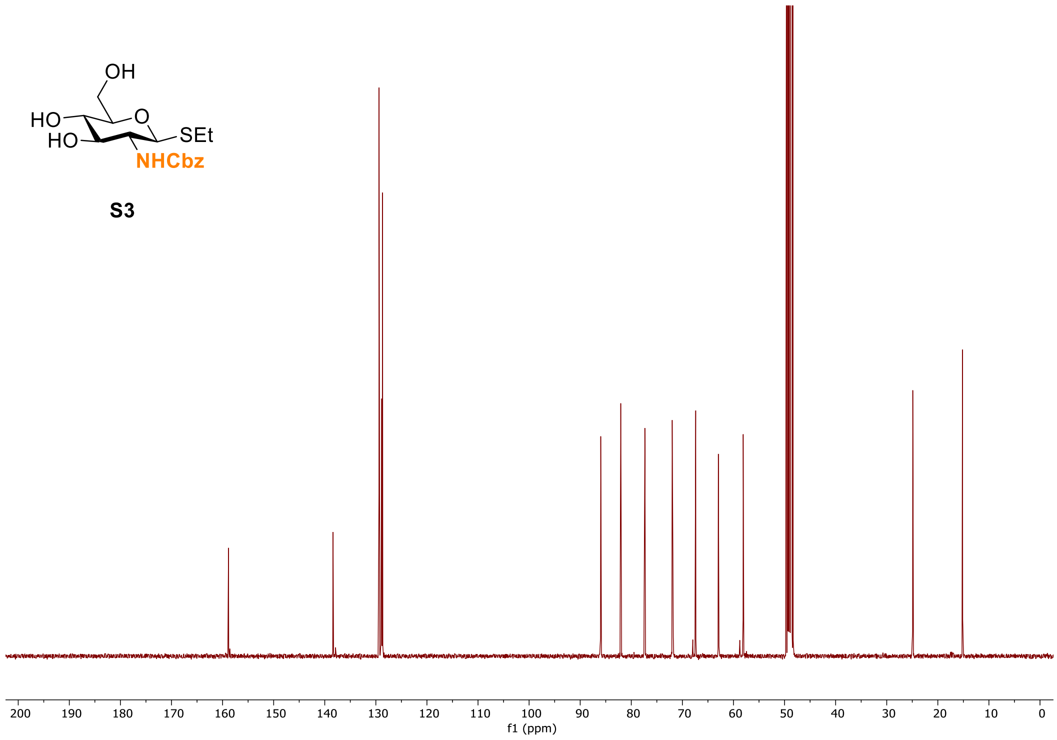

HSQC NMR of S3 (MeOD)

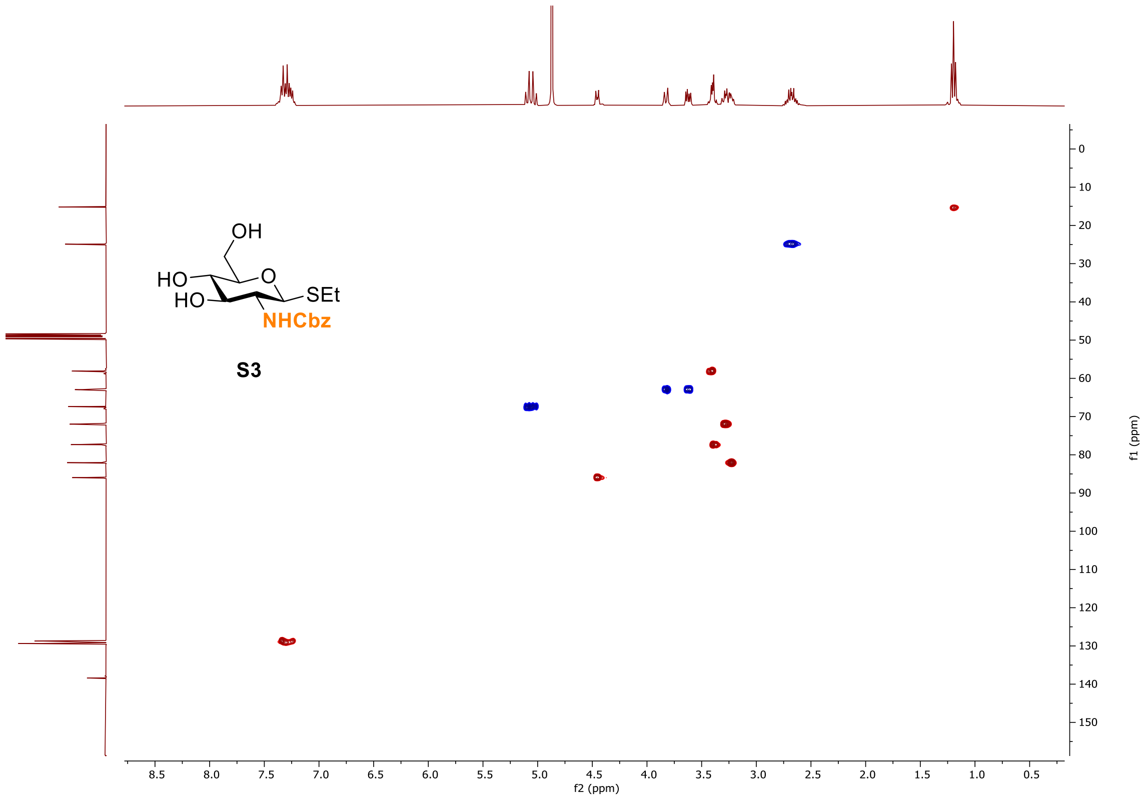

Ethyl 4,6-*O*-benzylidene-2-deoxy-2-(benzyloxycarbonyl)amino-1-thio-β-D-glucopyranoside, S4

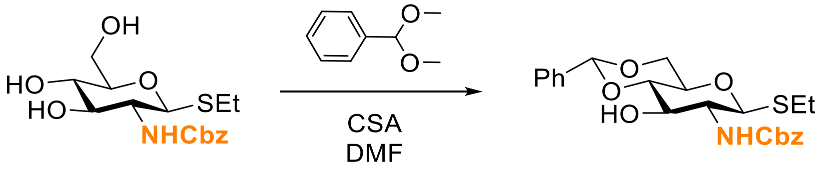

S3 **S4**

Ethyl 2-(benzyloxycarbonyl)amino-2-deoxy-1-thio-β-D-glucopyranoside **S3** (147 mg, 411 µmol) was dissolved in DMF (2 mL) under Argon atmosphere and a catalytic amount of DL-camphor-10-sulfonic acid (23.9 mg, 103 µmol) was added to the mixture. Benzaldehyde dimethyl acetal (68.1 µL, 452 µmol) was then added dropwise at room temperature to the stirred solution. After 3 h, the reaction was quenched with triethylamine (20 µL). The reaction mixture was diluted with DCM and washed three times with $NaHCO_3$ sat. aq. solution, once with brine and once with H_2O. The organic layer was dried over Na_2SO_4 and concentrated under reduced pressure. The crude mixture was purified by column chromatography (Ethyl acetate: Toluene = 3:7, R_f = 0.3) to yield **S4** as a white solid (172 mg, 93%).

^{1}H NMR (400 MHz, $CDCl_3$) δ 7.49 – 7.47 (m, 2H), 7.36 – 7.301 (m, 8H), 5.51 (s, 1H), 5.13 (s, 2H), 5.08 (d, *J* = 8.3 Hz, 1H), 4.61 (d, *J* = 10.4 Hz, 1H), 4.32 (dd, *J* = 10.4, 4.8 Hz, 1H), 3.98-3.93 (m, 1H), 3.73 (t, *J* = 10.1 Hz, 1H), 3.56 – 3.42 (m, 3H), 2.73 – 2.69 (m, 2H), 1.25 (t, *J* = 7.4 Hz, 4H); ^{13}C NMR (101 MHz, $CDCl_3$) δ 192.62, 156.57, 137.08, 136.19, 134.63, 129.90, 129.46, 129.14, 128.69, 128.50, 128.39, 128.24, 126.47, 101.99,

84.68, 81.38, 72.83, 70.45, 68.67, 67.39, 57.57, 29.84, 24.53, 14.99; $[\alpha]_D^{20}$ -51.14 (*c* g/100 mL, CH_2Cl_2); IR (neat) ν_{max} = 3470.01, 3305.68, 2884.29, 1688.07, 1546.17, 1290.12, 1262.54, 1104.45, 994.80 cm^{-1}; (ESI-HRMS) m/z 468.1461 $[M+Na]^+$ ($C_{23}H_{27}NO_6SNa$ requires 468.1457).

^{1}H NMR of S4 (400 MHz, $CDCl_3$)

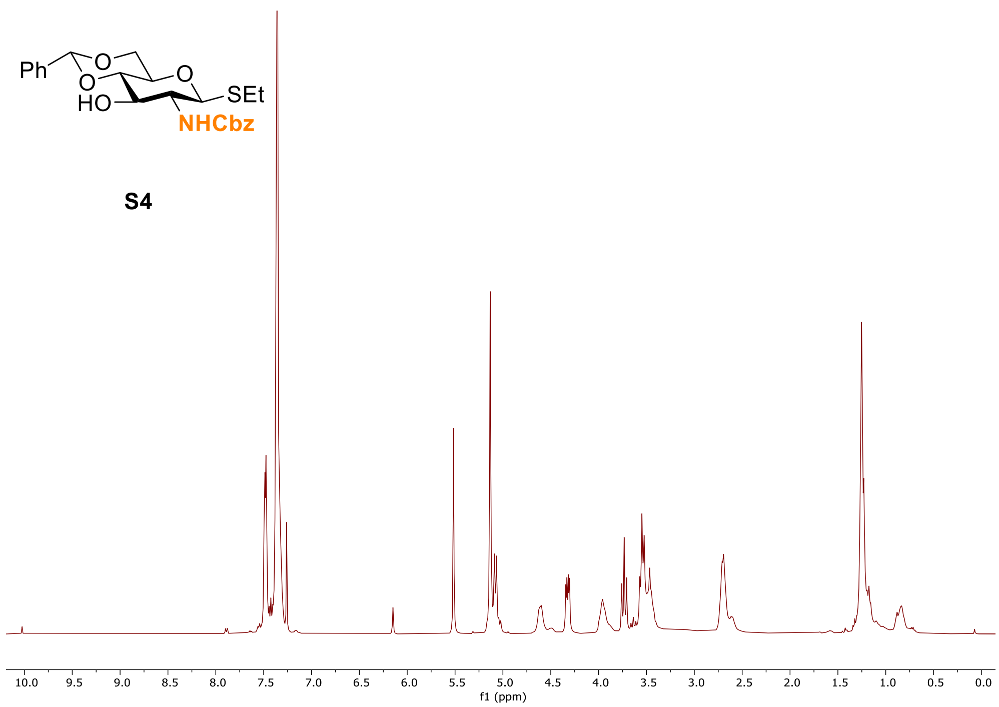

^{13}C NMR of S4 (101 MHz, $CDCl_3$)

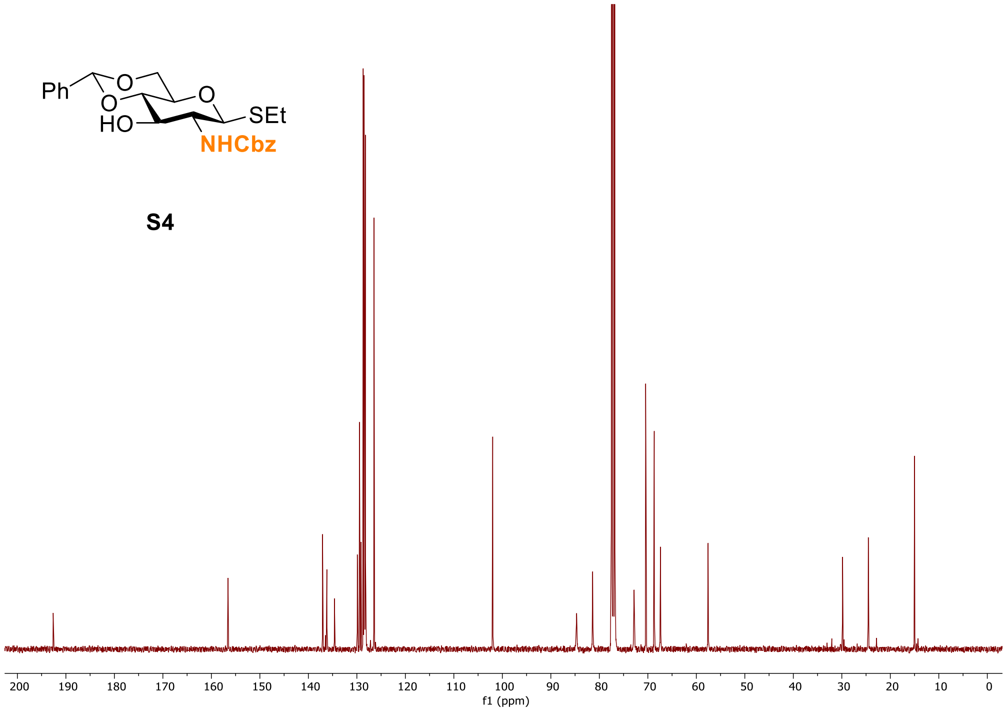

HSQC NMR of S4 ($CDCl_3$)

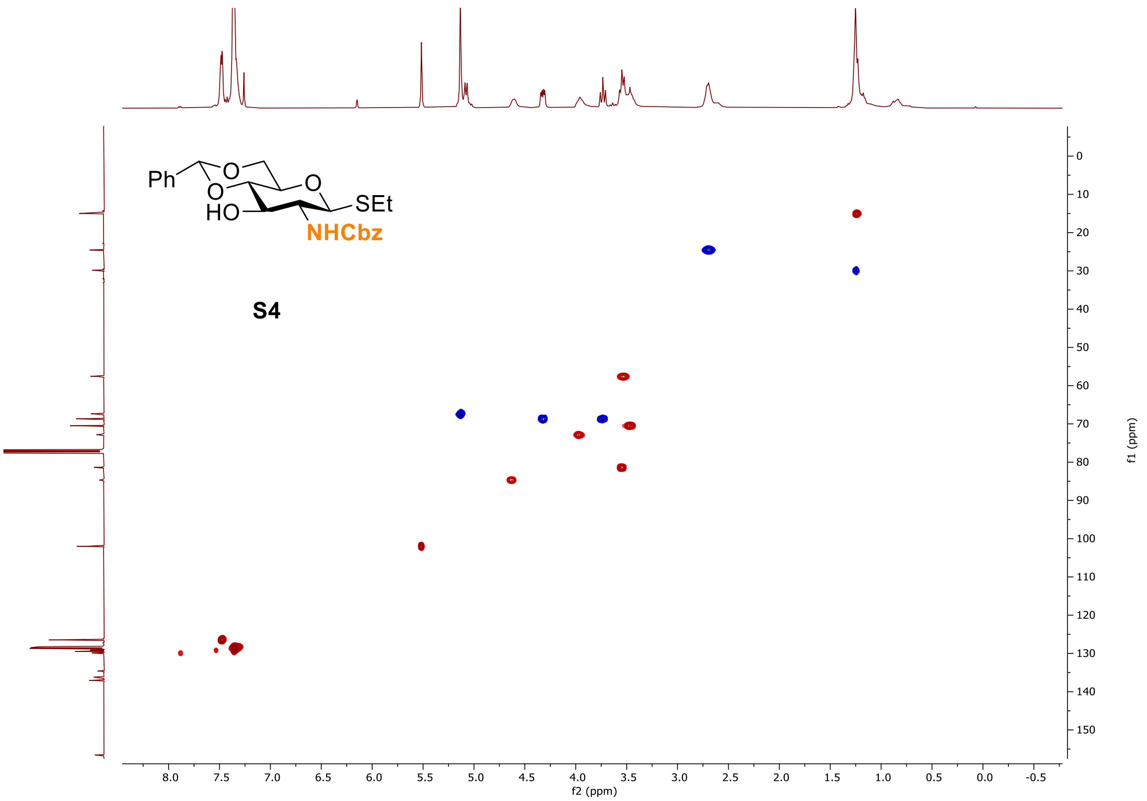

Ethyl 3-*O*-benzyl-4,6-*O*-benzylidene-2-deoxy-2-(benzyloxycarbonyl)amino-1-thio-β-D-glucopyranoside, S5

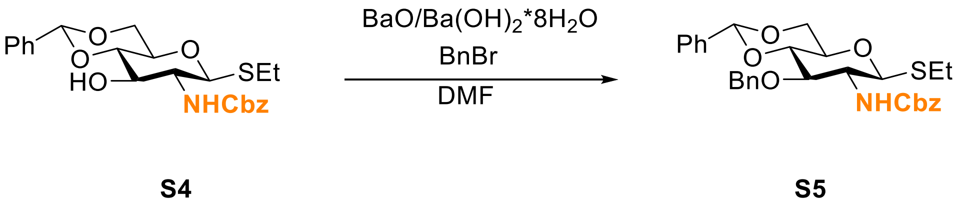

A solution of ethyl 4,6-*O*-benzylidene-2-(benzyloxycarbonyl)amino-2-deoxy-1-thio-β-D-glucopyranoside **S4** (11.0 g, 24.7 mmol) in anhydrous DMF (250 mL) was stirred overnight at room temperature in the presence of barium oxide (15.1 g, 98.8 mmol), barium hydroxide octahydrate (4.11 mg, 13.0 mmol) and benzyl bromide (3.82 mL, 32.1mmol) under Argon atmosphere. The reaction was filtered through a celite pad and washed with DCM (3 x 50 mL). The organic extracts were washed with water, $NaHCO_3$ sat. aq and H_2O, dried over Na_2SO_4 and concentrated under reduced pressure. The crude mixture was recrystallized from EtOAc – hexane (Ethyl acetate: Toluene = 1:4, R_f = 0.6) to yield **S5** as white solid (11.1 g, 84%).

^{1}H NMR (400 MHz, $CDCl_3$) δ 7.54 – 7.44 (m, 2H), 7.43 – 7.25 (m, 13H), 5.61 (s, 1H), 5.13 (d, J = 12.3 Hz, 1H), 5.09 (d, J = 12.3 Hz, 1H), 4.94 – 4.74 (m, 2H), 4.66 (d, J = 11.6 Hz, 1H), 4.38 (dd, J = 10.5, 5.0 Hz, 1H), 3.99 (bs, 1H), 3.81 (t, J = 10.3 Hz, 1H), 3.75 (t, J = 9.2 Hz, 1H), 3.56 – 3.38 (m, 2H), 2.78 – 2.61 (m, 2H), 1.26 (t, J = 7.4 Hz, 3H); ^{13}C NMR (101 MHz, $CDCl_3$) δ 155.78, 138.12, 137.40, 136.49, 129.17, 128.66, 128.50, 128.44, 128.41, 128.32, 128.21, 127.94, 126.14, 101.31, 84.71, 82.56, 78.04, 74.76, 70.48, 68.81, 67.00, 57.02, 24.65, 15.06; $[\alpha]_D^{20}$ -14.73 (c g/100 mL, CH_2Cl_2); IR (neat) ν_{max} = 3302.78, 2946.04, 2834.91, 1696.09, 1558.25, 1452.62, 1025.11 cm^{-1}; (ESI-HRMS) m/z 558.1937 $[M+Na]^+$ ($C_{30}H_{33}NO_6SNa$ requires 558.1926).

^{1}H NMR of S5 (400 MHz, $CDCl_3$)

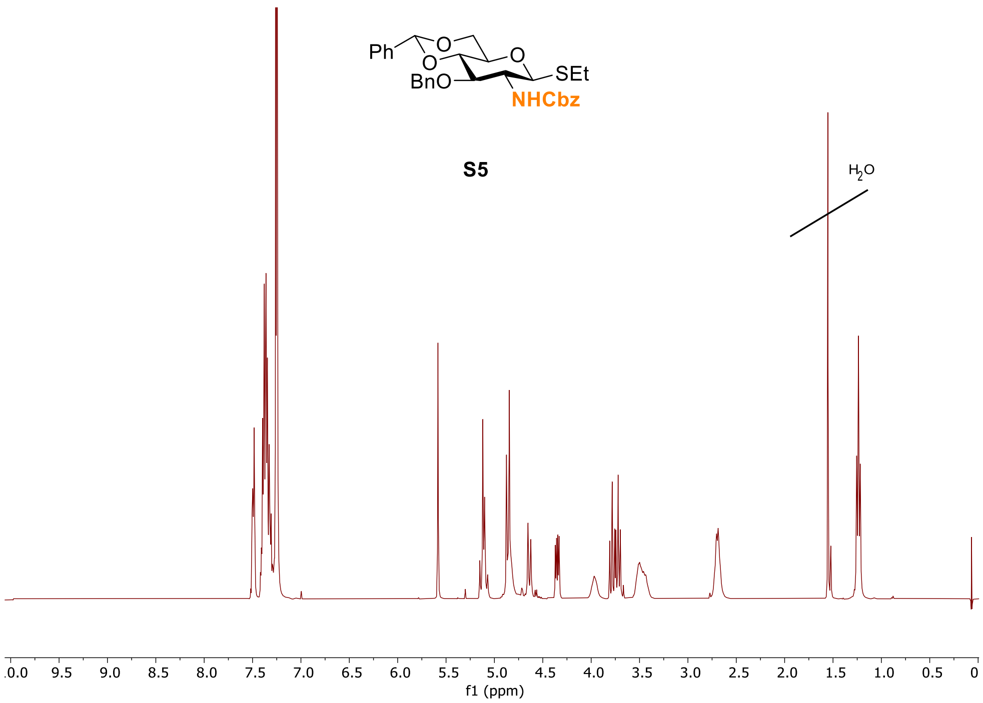

^{13}C NMR of S5 (101 MHz, $CDCl_3$)

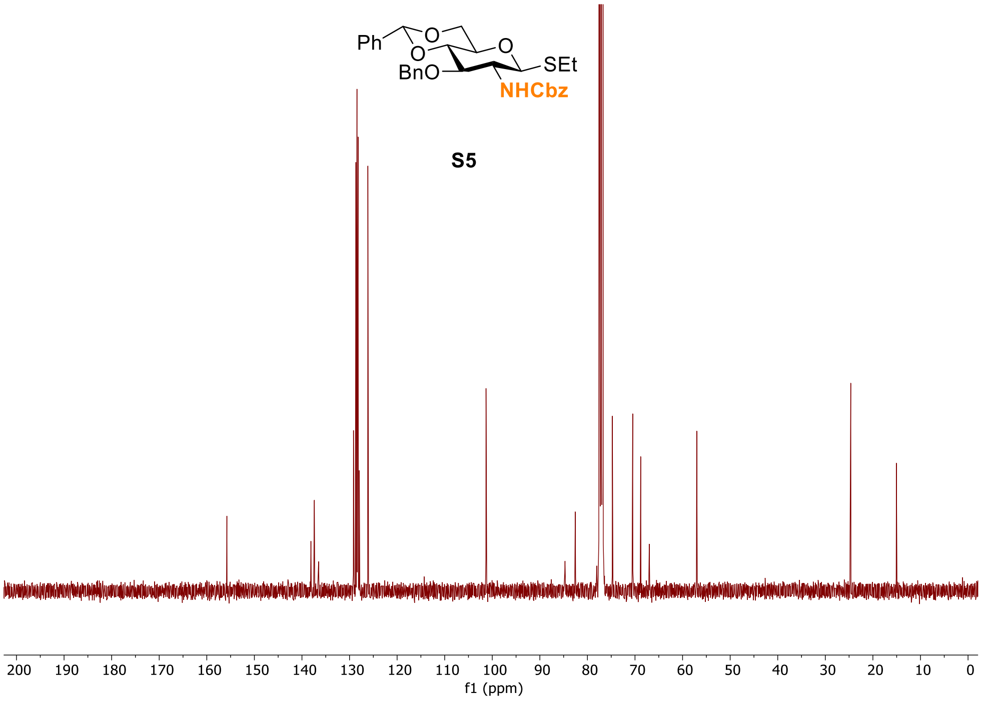

HSQC NMR of S5 ($CDCl_3$)

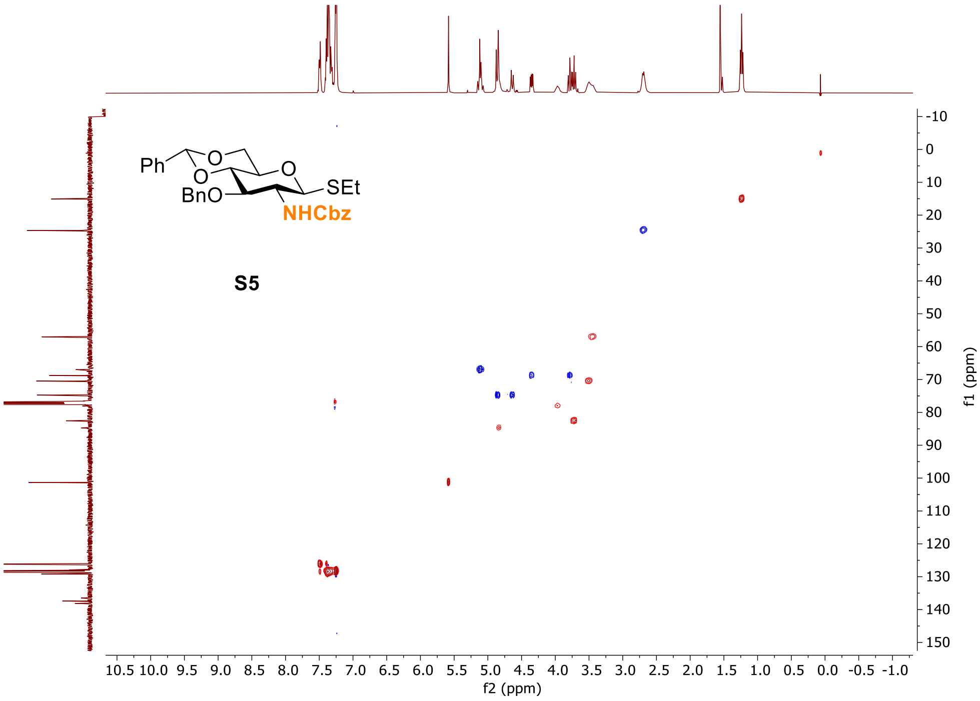

Ethyl 3,6-di-*O*-benzyl-2-deoxy-2-(benzyloxycarbonyl)amino-1-thio-β-D-glucopyranoside, S6

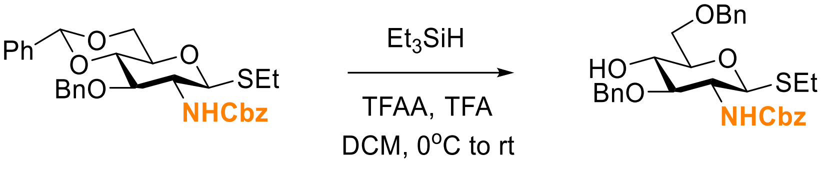

Ethyl 3-*O*-benzyl-4,6-*O*-benzylidene-2-deoxy-2-(benzyloxycarbonyl)amino-1-thio-β-D-glucopyranoside **S5** (146 mg, 273 µmol) was dissolved in anhydrous DCM (2 mL) under Argon atmosphere. $HSiEt_3$ (261 µL, 1.64 mmol) and TFAA (38.0 µL, 273 µmol) were sequentially added to the stirred solution at 0 °C. After 20 min, TFA (121 µL, 1.64 mmol) was added dropwise. The reaction was allowed to room temperature and, after 1 h, quenched with $NaHCO_3$ sat. aq. solution. The organic layer was washed twice with $NaHCO_3$ sat. aq. solution and once with brine. The crude mixture was purified by column chromatography (Ethyl acetate: Toluene = 1:3, R_f = 0.3) to yield **S6** as a white solid (115 mg, 78%).

^{1}H NMR (400 MHz, $CDCl_3$) δ 7.37 – 7.27 (m, 15H), 5.14 (d, *J* = 12.2 Hz, 1H), 5.10 (d, *J* = 12.3 Hz, 1H), 4.91 (d, *J* = 8.6 Hz, 1H), 4.72 (d, *J* = 11.4 Hz, 1H), 4.70 (d, *J* = 10.9 Hz, 1H), 4.59 (d, *J* = 11.9 Hz, 1H), 4.55 (d, *J* = 11.9 Hz, 1H), 3.76 – 3.73 (m, 2H), 3.70 – 3.66 (m, 2H), 3.51 – 3.42 (m, 2H), 2.71 – 2.66 (m, 2H), 1.24 (t, *J* = 7.4 Hz, 3H); ^{13}C NMR (101 MHz, $CDCl_3$) δ 155.86, 138.27, 137.79, 136.54, 129.16, 128.63, 128.62, 128.59, 128.46, 128.35, 128.26, 128.23, 128.18, 128.00, 127.96, 127.87, 125.42, 84.06, 82.23, 77.88, 74.53, 73.80, 73.14, 70.82, 66.96, 56.33, 29.83, 24.40, 15.06; $[\alpha]_D^{20}$ -15.83 (*c* g/100 mL, CH_2Cl_2); IR (neat) ν_{max} = 3293.90, 2928.09, 1686.20, 1547.61, 1455.37, 1216.27, 1059.09 cm^{-1}; (ESI-HRMS) m/z 576.1835 $[M+K]^+$ ($C_{30}H_{35}NO_6SK$ requires 576.1822)

^{1}H NMR of S6 (400 MHz, $CDCl_3$)

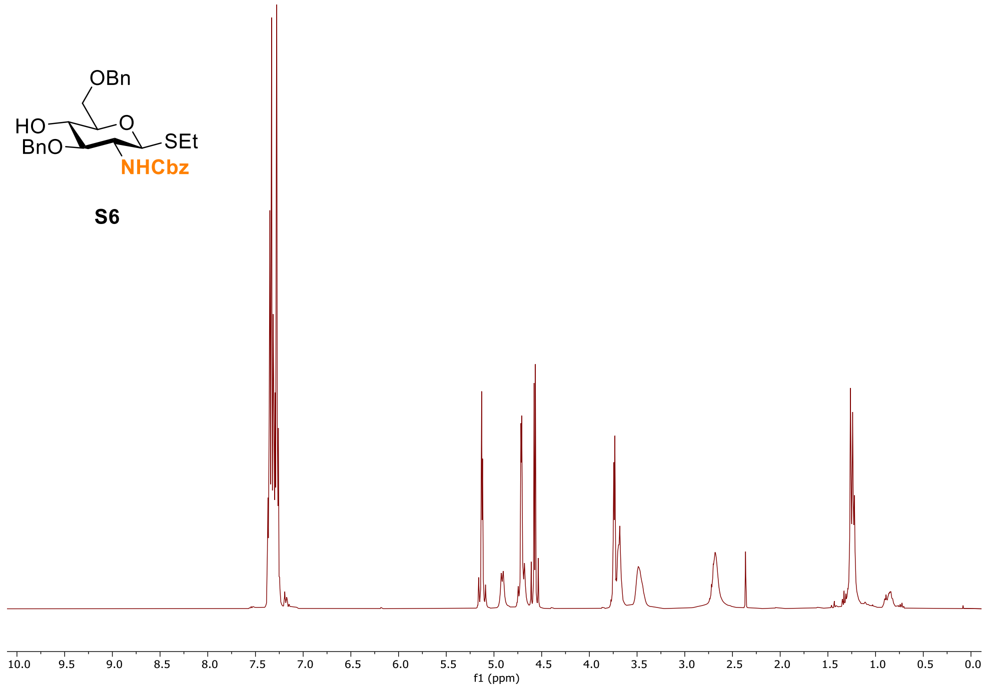

^{13}C NMR of S6 (101 MHz, $CDCl_3$)

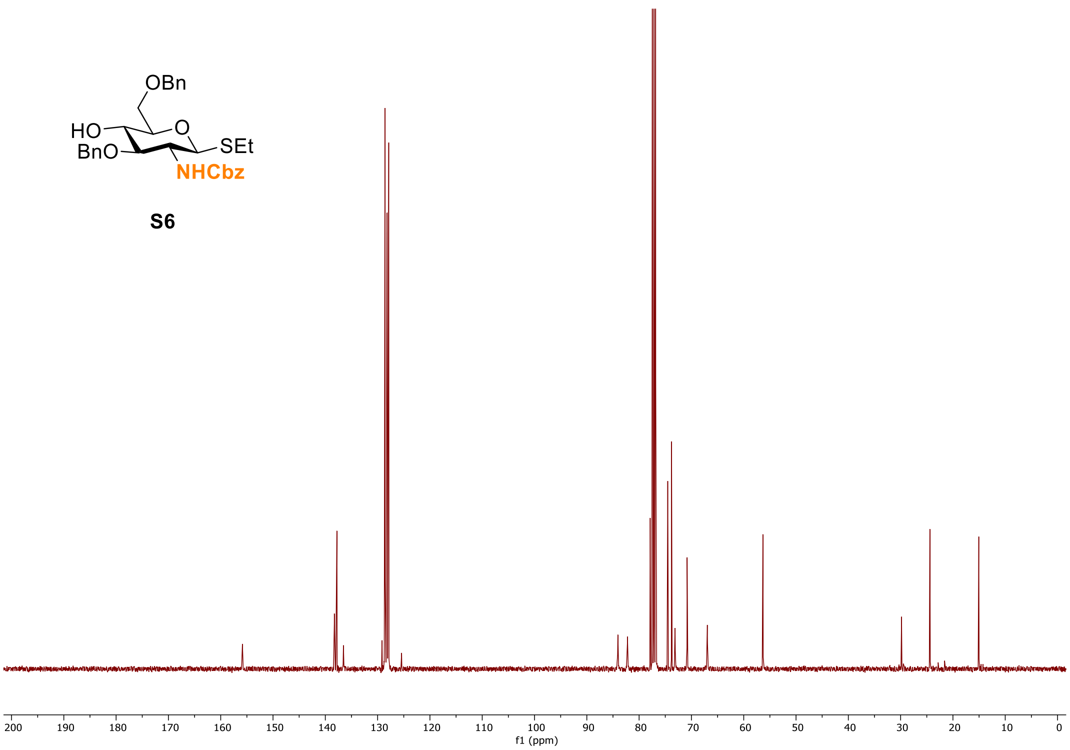

HSQC NMR of S6 ($CDCl_3$)

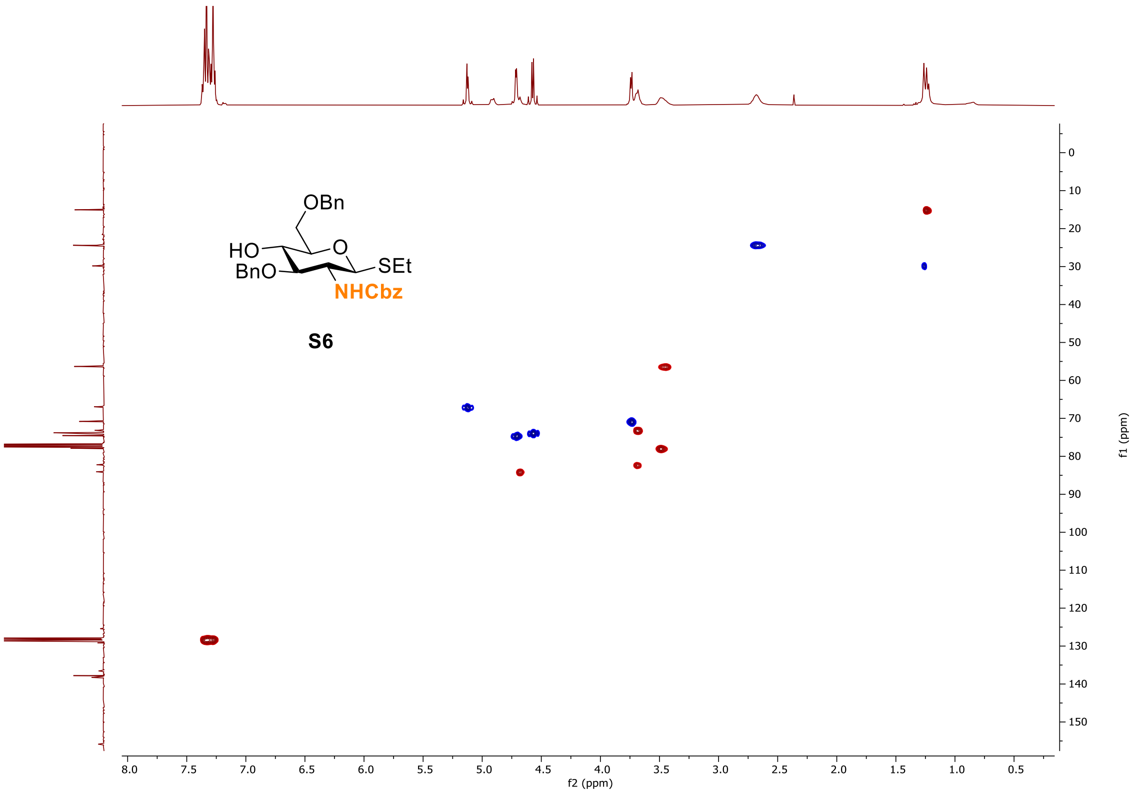

Ethyl 3,6-di-*O*-benzyl-4-*O*-(9-fluorenylmethoxycarbonyl)-2-deoxy-2-(benzyloxycarbonyl)amino-1-thio-β-D-glucopyranoside, S7

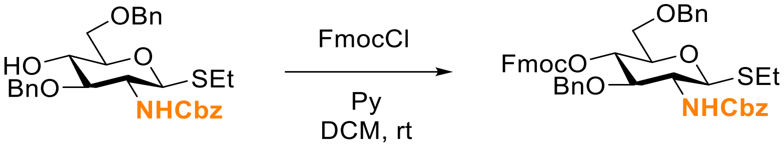

S6 **S7**

Ethyl 3,6-di-*O*-benzyl-2-deoxy-2-(benzyloxycarbonyl)amino-1-thio-β-D-glucopyranoside **S6** (1.9 g, 3.6 mmol) was dissolved in DCM (50 mL) and pyridine was added (1.2 mL, 14 mmol). FmocCl (2.3 mg, 9.0 mmol) was then added to the reaction mixture under Argon atmosphere. The yellow solution was stirred for 2 h, after which time it was quenched with a 1 M solution of HCl. The organic layer was washed one time with 1 M HCl, one time with sat. aq. solution of $NaHCO_3$ and one time with sat. aq. solution of NaCl. The crude mixture was recrystallized from EtOAc – hexane (Ethyl acetate: Hexane = 3:7, R_f = 0.3) to yield **S7** as a pale yellow solid (2.4 g, 87%).
^{1}H NMR (400 MHz, $CDCl_3$) δ 7.78 (dd, *J* = 7.5, 3.0 Hz, 2H), 7.60 (dd, *J* = 7.5, 0.6 Hz, 1H), 7.55 (dd, *J* = 7.5, 0.6 Hz, 1H), 7.43 – 7.11 (m, 19H), 5.14 (s, 2H), 5.00 (d, *J* = 7.2 Hz, 1H), 4.93 (d, *J* = 9.5 Hz, 1H), 4.88 (d, *J* = 10.2 Hz, 1H), 4.63 (d, *J* = 11.3 Hz, 1H), 4.59 – 4.48 (m, 3H), 4.33 (d, *J* = 7.2 Hz, 2H), 4.14 (t, *J* = 7.2 Hz, 1H), 4.08 (t, *J* = 8.7 Hz, 1H), 3.82 – 3.70 (m, 1H), 3.70 – 3.59 (m, 2H), 3.52 – 3.34 (m, 1H), 2.79 – 2.60 (m, 2H), 1.29 (t, *J* = 7.5 Hz, 30H); ^{13}C NMR (101 MHz, $CDCl_3$) δ 155.65, 154.44, 143.41, 143.21, 141.39, 141.35, 138.01, 137.71, 136.45, 128.64, 128.42, 128.30, 128.18, 128.02, 127.87, 127.71, 127.29, 127.28, 125.23, 125.13, 120.18, 120.17, 83.79, 79.53, 76.23, 74.47, 73.61, 70.09, 69.83, 66.96, 56.94, 46.74, 24.65, 15.13; $[\alpha]_D^{20}$ +45.87 (*c* g/100 mL, CH_2Cl_2); IR (neat) ν_{max} = 3332.60, 2963.02, 1748.00, 1688.443, 1540.85, 1452.39, 1261.18, 1064.96 cm^{-1}; (ESI-HRMS) m/z 782.2811 $[M+Na]^+$ ($C_{45}H_{45}NO_8SNa$ requires 782.2764).

^{1}H NMR of S7 (400 MHz, $CDCl_3$)

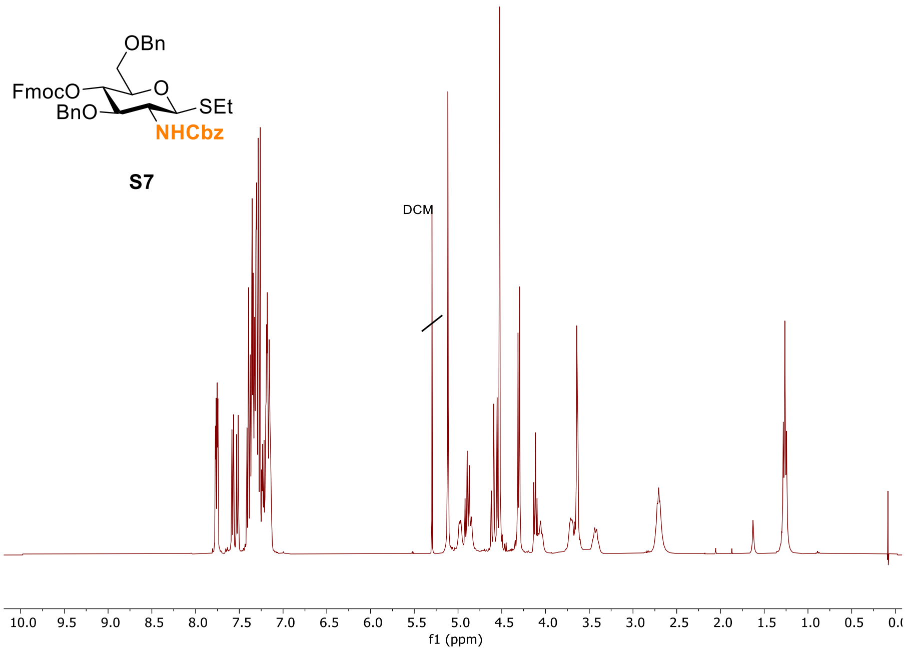

^{13}C NMR of S7 (101 MHz, $CDCl_3$)

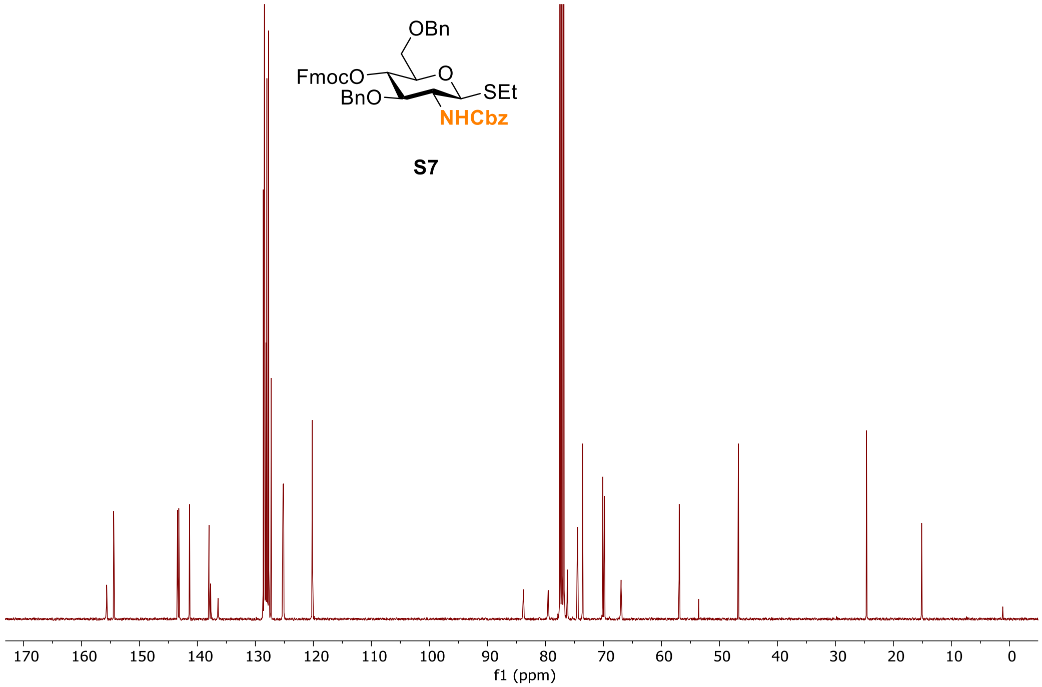

HSQC NMR of S7 ($CDCl_3$)

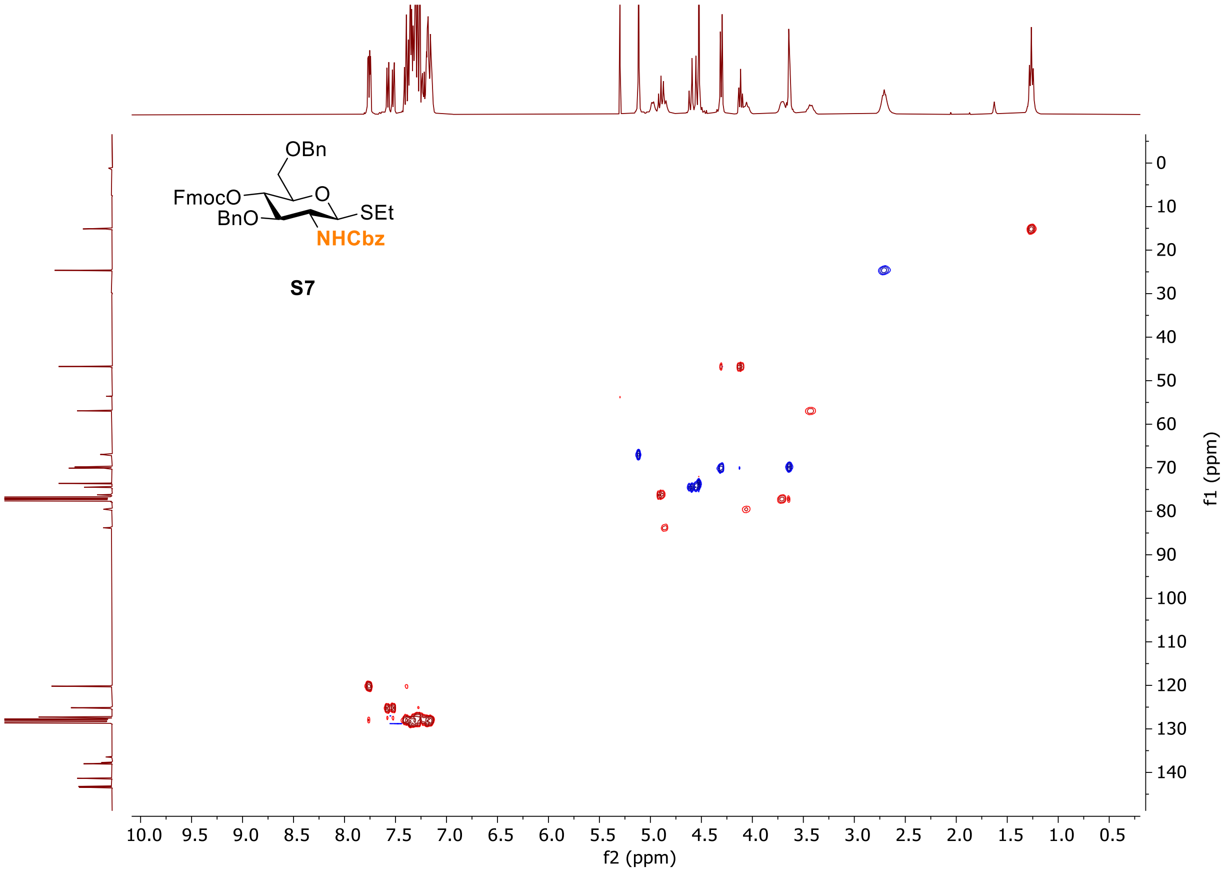

Dibutylphosphoryloxy 3,6-di-*O*-benzyl-4-*O*-fluorenylmethoxycarbonyl-2-deoxy-2-(benzyloxycarbonyl)amino-α/β-D-glucopyranoside, BB2

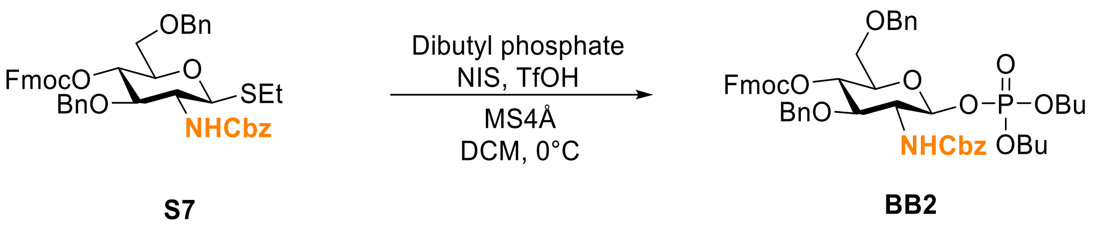

Compound **S7** (83.0 mg, 109 µmol) was coevaporated three times with toluene and dried in high vacuum overnight, after which time it was dissolved in DCM (2.4 mL) under Argon atmosphere. Dibutyl phosphate (64.8 µL, 327 µmol) and 4 Å molecular sieves were added to the solution. The mixture was stirred for 1 h and then cooled to 0 °C. NIS (30.2 mg, 134 µmol) and TfOH (0.68 µL, 7.63 µmol) were added. After 30 min the reaction was carefully quenched with NEt_3 (1 µL), diluted with DCM and filtered through a bed of Celite. The organic solution was washed once with 10% aqueous $Na_2S_2O_3$ and saturated aqueous $NaHCO_3$ and once with water. The organic phase was dried over Na_2SO_4, filtered and concentrated. The crude mixture was purified by column chromatography (EtOAc: Hexane = 1:2, R_f = 0.25) to obtain **BB2** as a sticky colorless solid (71.6 mg, 72%, β anomer).

^{1}H NMR (400 MHz, $CDCl_3$, β anomer) δ 7.84 (dd, *J* = 7.6, 4.3 Hz, 2H), 7.64 (dd, *J* = 14.3, 7.5 Hz, 2H), 7.49 – 7.45 (m, 2H), 7.40 – 7.27 (m, 17H), 5.77 (dd, *J* = 6.2, 3.2 Hz, 1H), 5.24 – 5.09 (m, 3H), 4.88 (d, *J* = 9.4 Hz, 1H), 4.71 (d, *J* = 11.4 Hz, 1H), 4.65 – 4.53 (m, 3H), 4.46 – 4.37 (m, 2H), 4.26 – 4.18 (m, 3H), 4.15 – 4.03 (m, 4H), 3.86 (t, *J* = 9.9 Hz, 1H), 3.71 – 3.64 (m, 2H), 1.69 – 1.61 (m, 4H), 1.45 – 1.37 (m, 4H), 0.98 – 0.94 (m, 6H). ^{13}C NMR (101 MHz, $CDCl_3$, β anomer) δ 155.82, 154.24, 143.34, 143.22, 141.42, 141.39, 137.74, 137.62, 136.23, 128.64, 128.47, 128.42, 128.35, 128.30, 128.04, 127.99, 127.91, 127.85, 127.79, 127.30, 125.19, 125.13, 120.20, 96.77, 96.70, 74.86, 73.93, 73.72, 70.92, 70.14, 68.80, 68.24, 68.18, 68.14, 68.08, 67.23, 54.28, 54.20, 46.77, 32.30, 32.23, 29.83, 18.69, 13.69; ^{31}P NMR (162 MHz, $CDCl_3$) δ -2.41; $[\alpha]_D^{20}$ +30.26 (*c* g/100 mL, CH_2Cl_2); IR (neat) ν_{max} = 3277.92, 2961.53, 1754.34, 1726.49, 1542.89, 1452.75, 1385.81, 1253.78, 1027.33 cm^{-1}; (ESI-HRMS) m/z 930.3658 $[M+Na]^+$ ($C_{51}H_{58}NO_{12}PNa$ requires 930.3594).

^{1}H NMR of BB2 (400 MHz, $CDCl_3$)

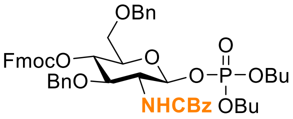

BB2

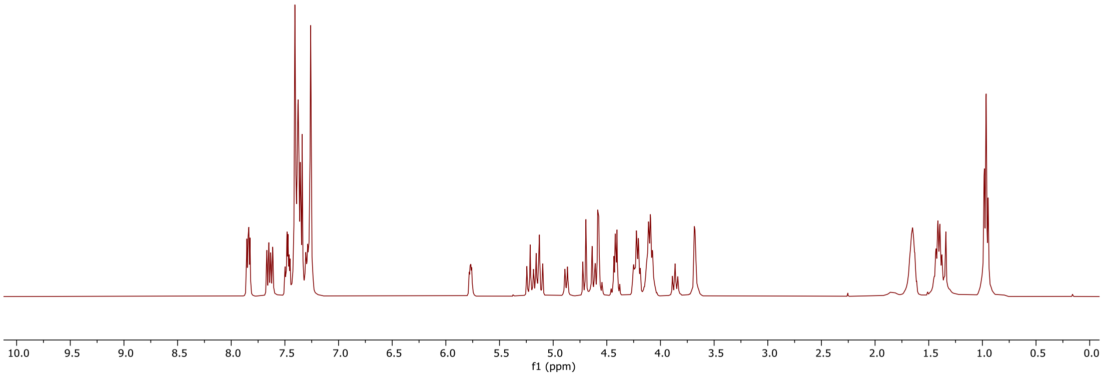

^{13}C NMR of BB2 (101 MHz, $CDCl_3$)

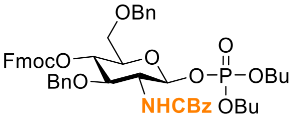

BB2

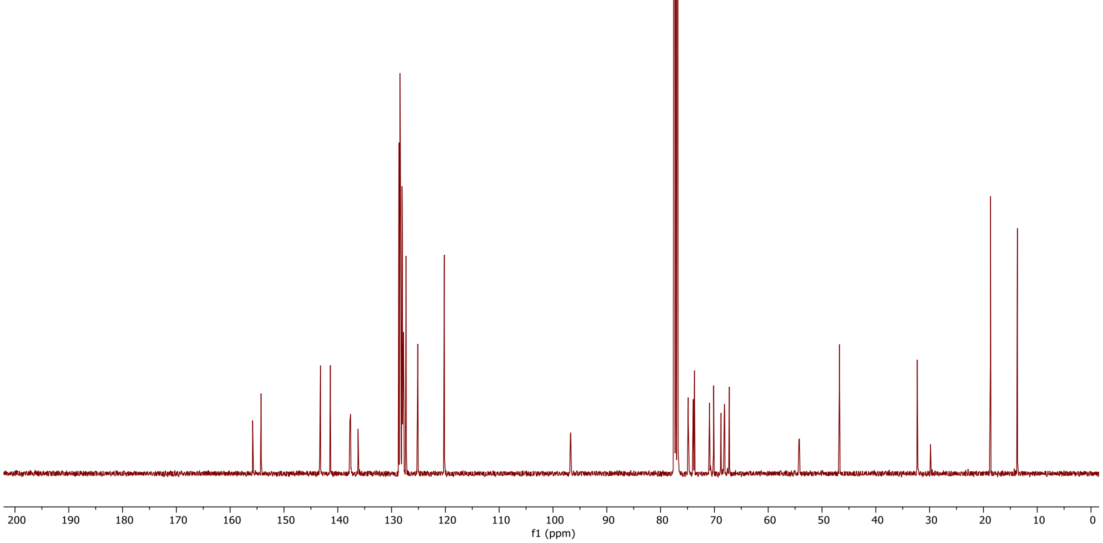

^{31}P NMR of BB2 (162 MHz, $CDCl_3$)

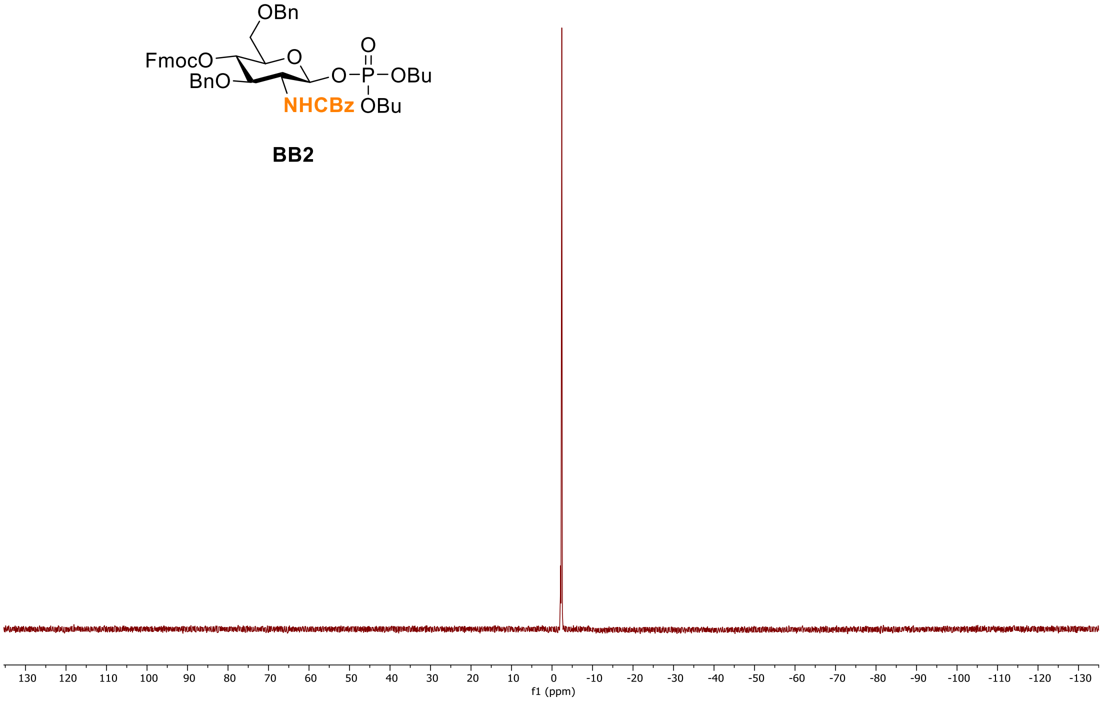

HSQC NMR of BB2 ($CDCl_3$)

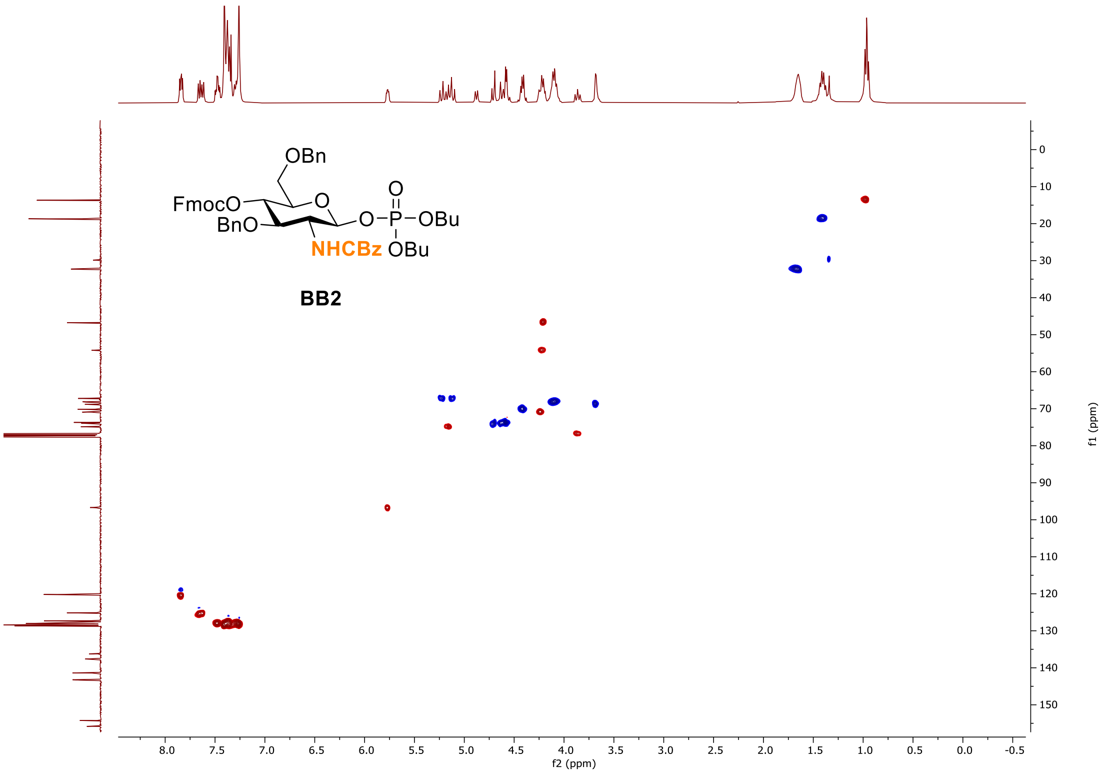

3. Automated Glycan Assembly

3.1 General Materials and Methods for AGA

All solvents used were HPLC-grade. The solvents used for the building block, activator, TMSOTf and capping solutions were taken from an anhydrous solvent system (jcmeyer-solvent systems). The building blocks were co-evaporated three times with toluene and dried for 1 h under high vacuum before use. Activator, capping, deprotection, acidic wash and building block solutions were freshly prepared and kept under argon during the automation run. All yields of products obtained by AGA were calculated on the basis of resin loading. Resin loading was determined following previously established procedures.[2]

3.2 Preparation of stock solutions

- **Building Block**: 0.06 mmol (5 equiv.) of glycosyl phosphate or 0.08 mmol (6.5 equiv.) of thioglycoside donor was dissolved in DCM (1 mL).
- **Activator solution**: 1.35 g of recrystallized NIS was dissolved in 40 mL of a 2:1 mixture of anhydrous DCM and anhydrous dioxane. Then triflic acid (55 µL) was added. The solution is kept at 0°C for the duration of the automation run.
- **Fmoc deprotection solution**: A solution of 20% piperidine in DMF (v/v) was prepared.
- **TMSOTf solution**: TMSOTf (0.45 mL) was added to DCM (40 mL).
- **Capping solution**: A solution of 10% acetic anhydride and 2% methanesulfunic acid in DCM (v/v) was prepared.

3.3 Modules for automated synthesis

Module A: Resin Preparation for Synthesis (20 min)

All automated syntheses were performed on 0.0125 mmol scale. Resin was placed in the reaction vessel and swollen in DCM for 20 min at room temperature prior to synthesis. During this time, all reagent lines needed for the synthesis were washed and primed. Before the first glycosylation, the resin was washed with the DMF, THF, and DCM (three times each with 2 mL for 25 s).

Module B: Acidic Wash with TMSOTf Solution (20 min)

The resin was swollen in 2 mL DCM and the temperature of the reaction vessel was adjusted to -20 °C. Upon reaching the low temperature, TMSOTf solution (1 mL) was added drop wise to the reaction vessel. After bubbling for 3 min, the acidic solution was drained and the resin was washed with 2 mL DCM for 25 s.

Action	Cycles	Solution	Amount	T (°C)	Incubation time
Cooling	-	-	-	-20	(15 min)*
Deliver	1	DCM	2 mL	-20	-

Deliver	1	TMSOTf solution	1 mL	-20	3 min
Wash	1	DCM	2 mL	-20	25 sec

*Time required to reach the desired temperature.

Module C: Thioglycoside Glycosylation (35 min)

The building block solution (0.08 mmol of BB in 1 mL of DCM per glycosylation) was delivered to the reaction vessel. After the set temperature was reached, the reaction was started by drop wise addition of the activator solution (1.0 mL, excess). The glycosylation conditions are building block dependent (we report the most common set of conditions). After completion of the reaction, the solution is drained and the resin washed with DCM (2 mL), DCM:dioxane (1:2, 3 mL for 20 s) and DCM (two times, each with 2 mL for 25 s). The temperature of the reaction vessel is increased to 25 °C for the next module.

Action	Cycles	Solution	Amount	T (°C)	Incubation time
Cooling	-	-	-	-20	-
Deliver	1	BB solution	1 mL	-20	-
Deliver	1	Activator solution	1 mL	-20	-
Reaction time (BB dependent)	1			-20 to 0	5 min 20 min
Wash	1	DCM	2 mL	0	5 sec
Wash	1	DCM : Dioxane (1:2)	2 mL	0	20 sec
Heating	-	-	-	25	-
Wash	2	DCM	2 mL	> 0	25 sec

Module C*: Glycosyl Phosphate Glycosylation (70 min)

The building block solution (0.06 mmol of BB in 1 mL of DCM per glycosylation) was delivered to the reaction vessel. After the set temperature was reached, the reaction was started by drop wise addition of the TMSOTf solution (1.0 mL, excess). The glycosylation conditions are building block dependent (we report the most common set of conditions). After completion of the reaction, the solution is drained and the resin washed with 2 mL DCE (2 mL) and with DCM (2 x 2 mL for 20 s). The temperature of the reaction vessel is increased to 25 °C for the next module.

Action	Cycles	Solution	Amount	T (°C)	Incubation time
Cooling	-	-	-	-35	(20 min)*
Deliver	1	BB solution	1 mL	-35	-
Deliver	1	TMSOTf solution	1 mL	-35	-
Reaction time (BB dependent)	1			-35 to -10	5 min 40 min

Wash	1	DCE	2 mL	0	1 min
Wash	2	DCM	2 mL	>0	22sec
Heating	-	-	-	25	3min

*Time required to reach the desired temperature.

Module D: Capping (30 min)

The resin was washed with DMF (two times with 2 mL for 25 s) and the temperature of the reaction vessel was adjusted to 25 °C. 2 mL of Pyridine solution (10% in DMF) was delivered into the reaction vessel. After 1 min, the reaction solution was drained and the resin washed with DCM (three times with 3 mL for 25 s). 4 mL of capping solution was delivered into the reaction vessel. After 20 min, the reaction solution was drained and the resin washed with DCM (three times with 3 mL for 25 s).

Action	Cycles	Solution	Amount	T (°C)	Incubation time
Heating	-	-	-	25	(5 min)*
Wash	2	DMF	2 mL	25	25 sec
Deliver	1	10% Pyridine in DMF	2 mL	25	1 min
Wash	3	DCM	2 mL	25	25 sec
Deliver	1	Capping Solution	4 mL	25	20 min
Wash	3	DCM	2 mL	25	25 sec

*Time required to reach the desired temperature.

Module E: Fmoc Deprotection (9 min)

The resin was washed with DMF (three times with 2 mL for 25 s) and the temperature of the reaction vessel was adjusted to 25 °C. 2 mL of Fmoc deprotection solution 1 was delivered to the reaction vessel. After 5 min, the reaction solution was drained and the resin washed with DMF (three times with 3 mL for 25 s) and DCM (five times each with 2 mL for 25 s). The temperature of the reaction vessel is decreased to -20 °C for the next module.

Action	Cycles	Solution	Amount	T (°C)	Incubation time
Wash	3	DMF	2 mL	25	25 sec
Deliver	1	Fmoc depr. Solution 1	2 mL	25	5 min
Wash	1	DMF	2 mL		
Cooling	-	-	-	-20	-
Wash	3	DMF	2 mL	< 25	25 sec
Wash	5	DCM	2 mL	< 25	25 sec

3.4 Post-synthesizer manipulations

Module F: On-resin Methanolysis

The resin was suspended THF (5 mL). 0.5 mL of NaOMe in MeOH (0.5 M) was added and the suspension was gently shaken at room temperature. After micro-cleavage (see **Module G1**) indicated the complete removal of benzoyl groups (generally around 4 hours), the resin was repeatedly washed with MeOH (2mL x 3) and DCM (2mL x 3).

Module G: Cleavage from Solid Support

The oligosaccharides were cleaved from the solid support using a continuous-flow photoreactor as described previously.[1b]

Module G*: Micro-cleavage from Solid Support

Trace amount of resin (around 20 beads) was dispersed in DCM (0.1 mL) and irradiated with a UV lamp (6 watt, 356 nm) for 10 minutes. ACN (10 µL) was then added to the resin and the resulting solution analyzed by MALDI.

Module H: Hydrogenolysis at ambient pressure

The crude compound obtained from *Module H* was dissolved in 2 mL of EA:*t*BuOH:H_2O (1:0.5:0.5). 100% by weight Pd-C (10%) was added and the reaction was stirred under H_2–atmosphere overnight. The reaction was filtered through celite and washed with EA, *t*BuOH and H_2O with a 0.1% of formic acid. The filtrates were concentrated *in vacuo.*

Module I: Purification

Purification was conducted at different stage of the synthesis as reported for the individual procedures. The crude products were analyzed using analytical HPLC (Agilent 1200 Series spectrometer, **Method A** and **Method C**). The purification was conducted using preparative HPLC (Agilent 1200 Series spectrometer) or C_{18} reverse phase silica gel column chromatography.

- **Method A:** (YMC-Diol-300 column, 150 x 4.6 mm) flow rate of 1.0 mL / min with Hex – 20% EtOAc as eluents [isocratic 20% EtOAc (5 min), linear gradient to 55% EtOAc (35 min), linear gradient to 100% EtOAc (5 min)].

- **Method B:** (YMC-Diol-300 column, 150 x 20 mm) flow rate of 15 mL / min with Hex – 20% EtOAc as eluents [isocratic 20% EtOAc (5 min), linear gradient to 55% EtOAc (35 min), linear gradient to 100% EtOAc (5 min)].

- **Method C:** (Hypercarb column, 150 x 4.6 mm) flow rate of 0.7 mL / min with H_2O (0.1% formic acid) as eluents [isocratic (5 min), linear gradient to 30% ACN (30 min), linear gradient to 100% ACN (5 min)].

- **Method D:** (Hypercarb column, 150 x 10 mm) flow rate of 1.3 mL / min with H_2O (0.1% formic acid) as eluents [isocratic (5 min), linear gradient to 30% ACN (30 min), linear gradient to 100% ACN (5 min)].

- **Method E:** (Manual size-exclusion reverse phase C_{18} column chromatography): isocratic H_2O – 50% MeOH.

- **Method F:** (Manual reverse phase C_{18} silica gel column chromatography): H_2O (10 mL), 5% MeOH (10 mL), 7.5% MeOH (10 mL), 10% MeOH (10 mL), 15% MeOH (10 mL), 20% MeOH (10 mL).

- **Method G:** (Hypercarb column, 150 x 4.6 mm) flow rate of 0.7 mL / min with H_2O (0.1% formic acid) as eluents [isocratic (5 min), linear gradient to 40% ACN (10 min), linear gradient to 100% ACN (10 min)].

Following final purification, all deprotected products were lyophilized on a Christ Alpha 2-4 LD plus freeze dryer prior to characterization.

3.5 Oligosaccharides synthesis

3.5.1 Synthesis of NNNNNN-OH

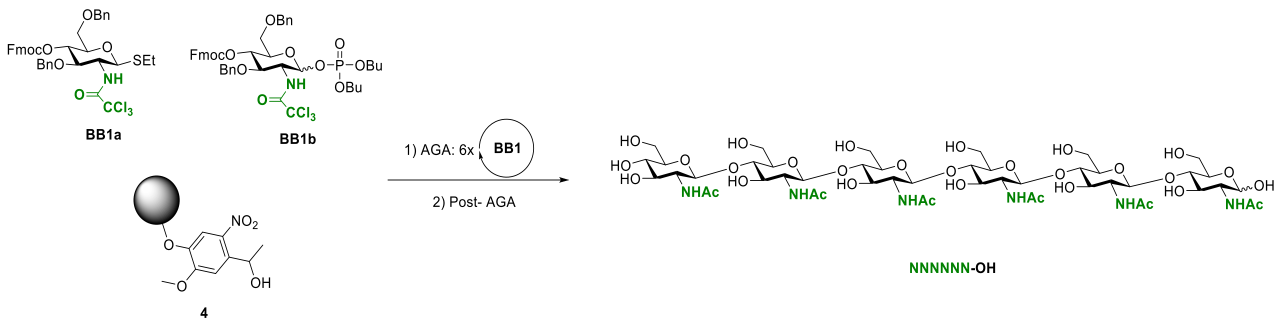

AGA was initially performed using **BB1a** (*Conditions1)*, affording **NNNNNN-OH** in a relatively low yield (1.3 mg, 8% overall yield). The use of the glycosyl phosphate donor **BB1b** (*Conditions2*) improved the results, affording **NNNNNN-OH** as white solid (5.3 mg, 34% overall yield). Both results are reported for comparison. Overall yields are considering AGA, global deprotection and purification.

Conditions1:

Step	Modules		Notes
AGA	6 x BB1a	**A**	
AGA		**B**, **C**, **D**, **E**	**C**:(**BB1a**, -20° for 5 min, 0° for 20 min)
Post-AGA		**G**, **I1**, **H**, **I2**	**I1**: (Method B, t_R = 32.0 and 34.0 min)<br>**I2**: (Method D, t_R = 29.0 and 30.1 min)

Conditions2:

Step	Modules		Notes
AGA	6 x BB1b	**A**	
AGA		**B**, **C***, **D**, **E**	**C***:(**BB1b**, -35° for 5 min, -10° for 40 min)
Post-AGA		**G**, **I1**, **H**, **I2**	**I1**: (Method B, t_R = 32.0 and 34.0 min)<br>**I2**: (Method D, t_R = 29.0 and 30.1 min)

Analytical data for **NNNNNN-OH**: ^{1}H NMR (600 MHz, D_2O) δ 5.40 – 5.38 (m, 1H), 5.21 (d, *J* = 2.8 Hz, 0.55H, α-H1), 4.71 (d, *J* = 8.2 Hz, 0.45H, β-H1), 4.60 (dd, *J* = 8.1, 5.4 Hz, 5H), 3.99 – 3.45 (m, 39H), 2.13 – 2.03 (m, 18H); ^{13}C NMR (151 MHz, D_2O) δ 174.53, 174.50, 174.37, 170.96, 101.37, 101.15, 94.74 (β-C1), 90.36 (α-C1), 79.54, 79.06, 78.83, 75.82, 74.49, 74.45, 74.42, 73.35, 72.04, 71.97, 69.91, 69.61, 69.15, 60.45, 59.92, 59.89, 59.85, 55.49, 54.98, 54.96, 54.96, 54.92, 54.92, 53.56, 22.08, 22.02; m/z (HRMS+) 1259.476 [M + Na]$^+$ ($C_{48}H_{81}N_6O_{31}Na$ requires 1259.477).

RP-HPLC of NNNNNN-OH (ELSD trace, Method D, t_R = 29.0 and 30.1 min)

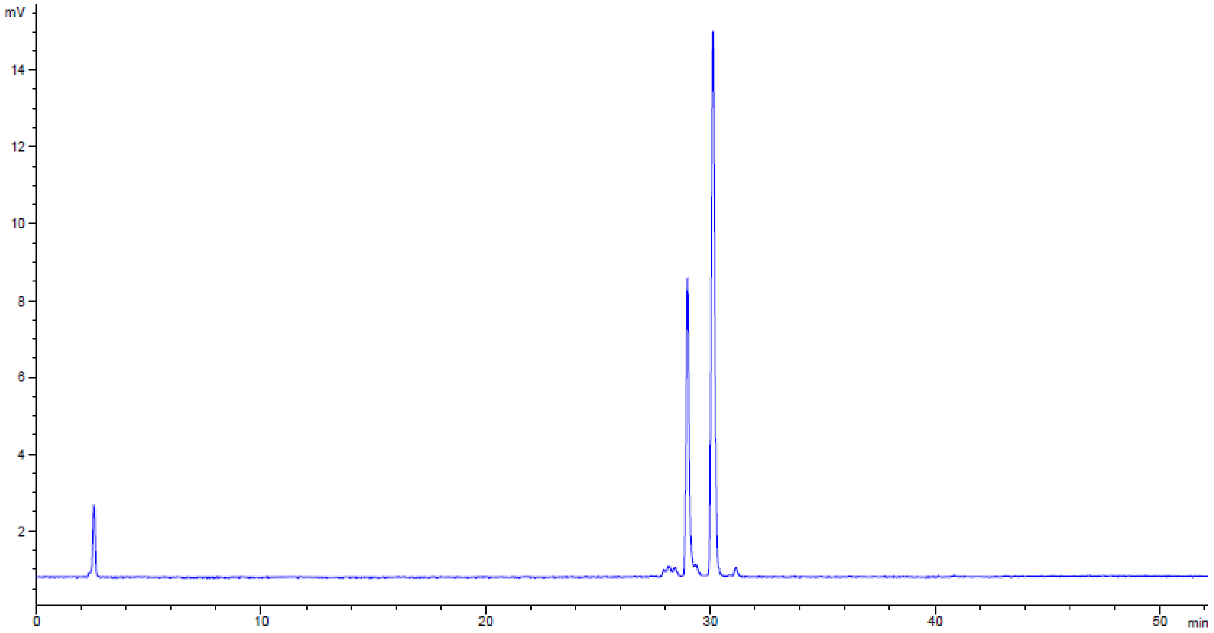

^{1}H NMR of NNNNNN-OH (600 MHz, D_2O)

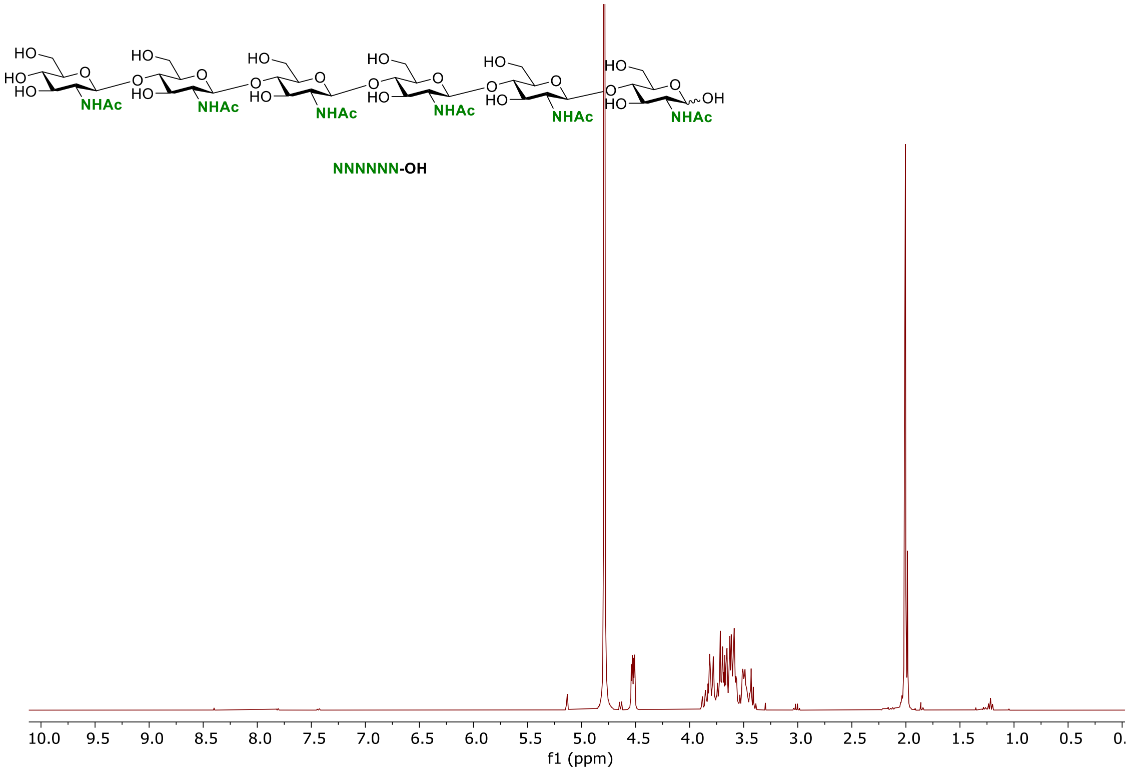

^{13}C NMR of NNNNNN-OH (151 MHz, D_2O)

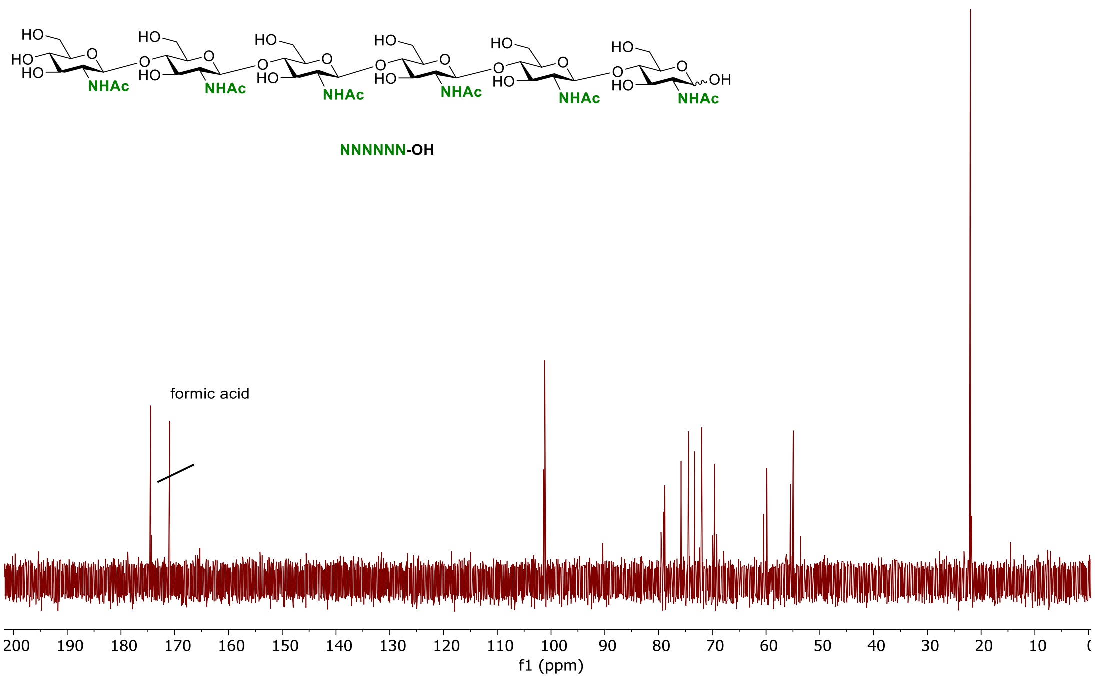

HSQC NMR of NNNNNN-OH (D_2O)

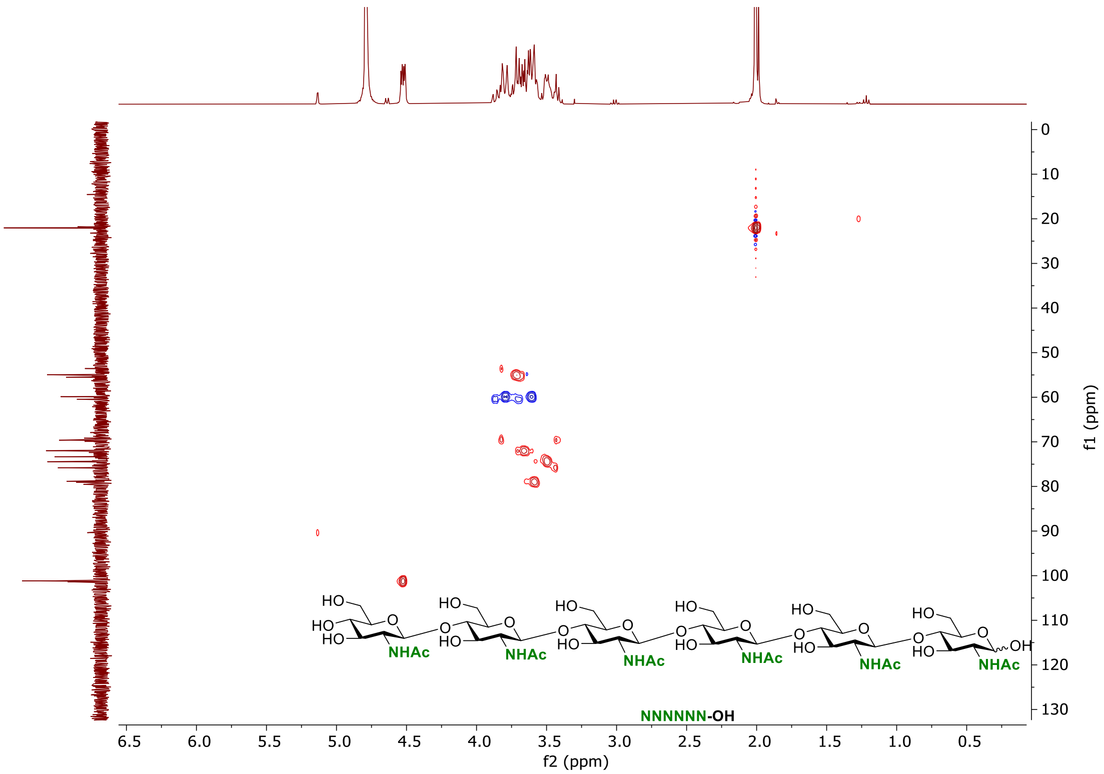

3.5.2 *Synthesis of ANAANA-OH*

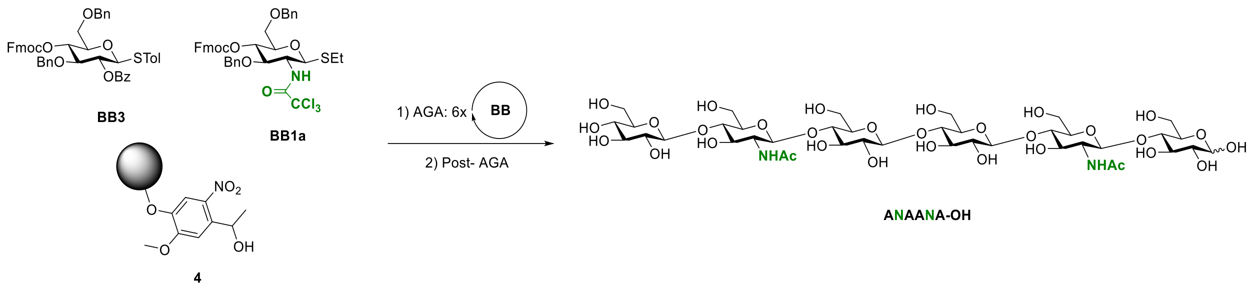

Step		Modules	Notes
		**A**	
AGA	**BB3**	**B**, **C**, **D**, **E**	**C**:(**BB3**, -20° for 5 min, 0° for 20 min)
	**BB1a**	**B**, **C**, **D**, **E**	**C**:(**BB1a**, -20° for 5 min, 0° for 20 min)
	**2 x BB3**	**B**, **C**, **D**, **E**	**C**:(**BB3**, -20° for 5 min, 0° for 20 min)
	**BB1a**	**B**, **C**, **D**, **E**	**C**:(**BB1a**, -20° for 5 min, 0° for 20 min)
	**BB3**	**B**, **C**, **D**, **E**	**C**:(**BB3**, -20° for 5 min, 0° for 20 min)
Post-AGA		**F**, **G**, **H**, **I**	**I**: (Method D, t_R = 35.8 and 36.2 min)

Automated synthesis, global deprotection, and purification afforded **ANAANA-OH** as white solid (3.8 mg, 28% overall yield).

Analytical data for **ANAANA-OH**: ^{1}H NMR (600 MHz, D_2O) δ 5.23 (d, *J* = 3.7 Hz, 0.38H), 4.67 (d, *J* = 8.0 Hz, 0.62H, β-H1), 4.63 – 4.50 (m, 5H), 4.00 (ddd, *J* = 20.1, 12.3, 2.1 Hz, 3H), 3.93 (dd, *J* = 12.4, 2.2 Hz, 1H), 3.90 – 3.49 (m, 28H), 3.43 (dd, *J* = 9.9, 9.1 Hz, 1H), 3.39 – 3.31 (m, 3H), 3.28 (dd, *J* = 9.4, 8.0 Hz, 0.6H), 2.08 (s, 6H); ^{13}C NMR (151 MHz, D_2O) δ 174.50, 102.49, 102.29, 102.17, 101.23, 101.22, 101.18, 95.60 (β-C1), 91.55 (α-C1), 79.29, 79.08, 78.88, 78.46, 78.41, 78.38, 78.08, 75.93, 75.40, 74.78, 74.70, 74.49, 74.46, 74.28, 74.05, 74.02, 73.91, 73.66, 73.08, 72.87, 72.70, 71.93, 71.91, 71.34, 69.36, 61.51, 60.68, 60.50, 60.05, 59.93, 59.91, 59.81, 59.78, 59.76, 55.26, 55.21, 22.04; m/z (HRMS+) 1095.368 $[M + Na]^+$ ($C_{40}H_{68}N_2O_{31}Na$ requires 1095.368).

RP-HPLC of ANAANA-OH (ELSD trace, Method D, t_R = 35.8 and 36.2 min)

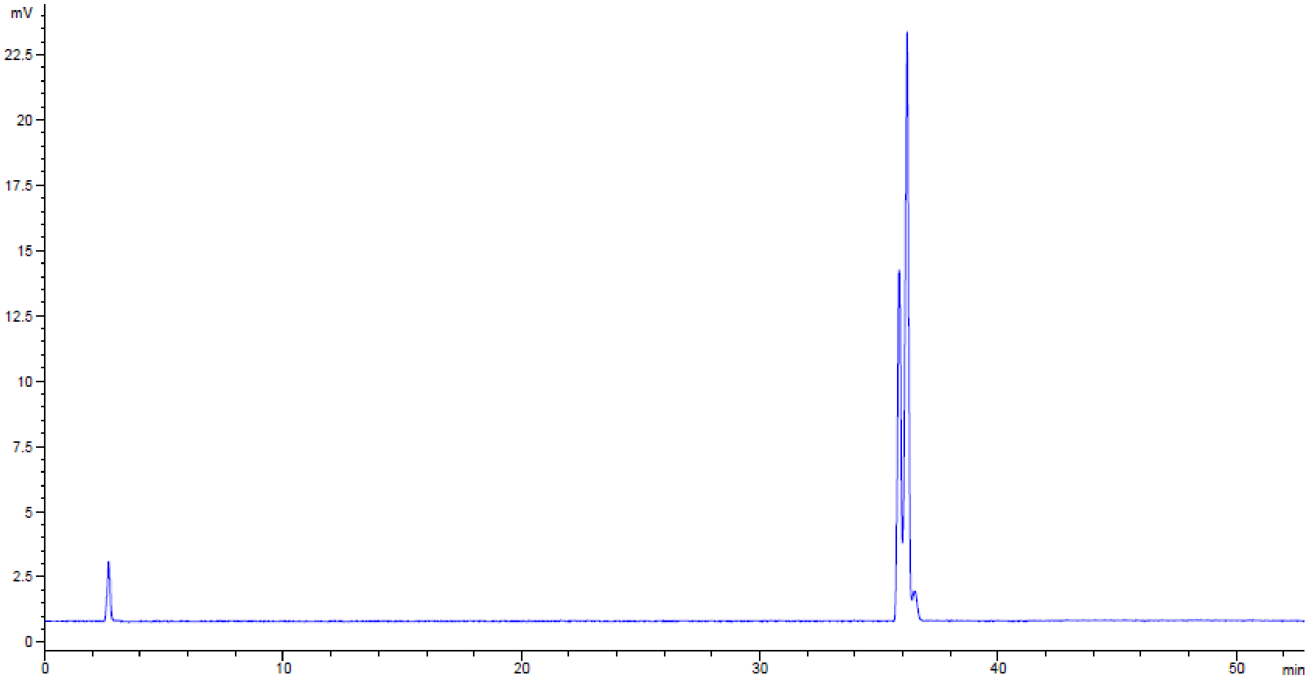

^{1}H NMR of ANAANA-OH (600 MHz, D_2O)

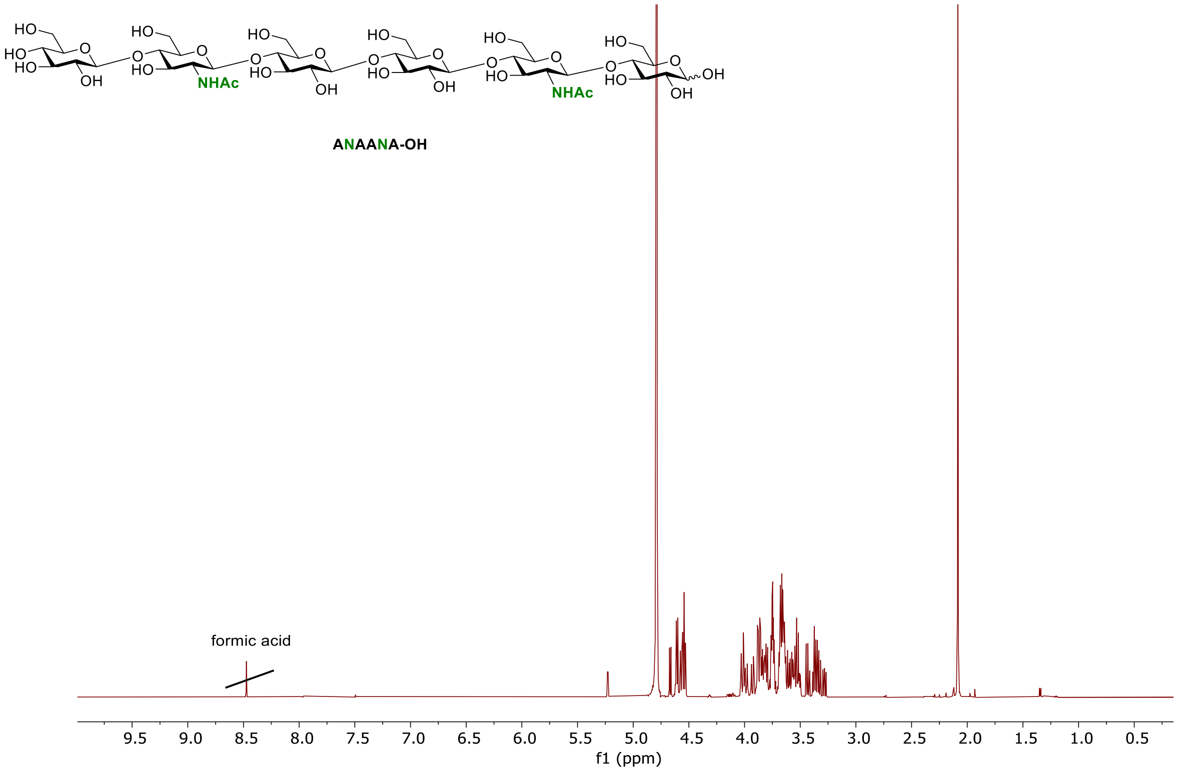

^{13}C NMR of ANAANA-OH (151 MHz, D_2O)

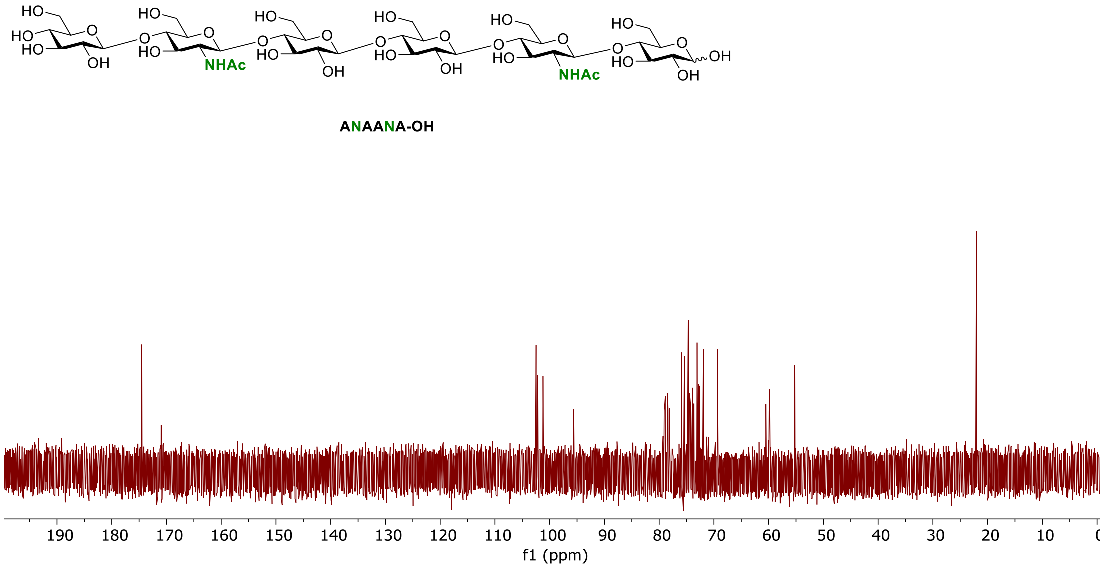

HSQC NMR of ANAANA-OH (D_2O)

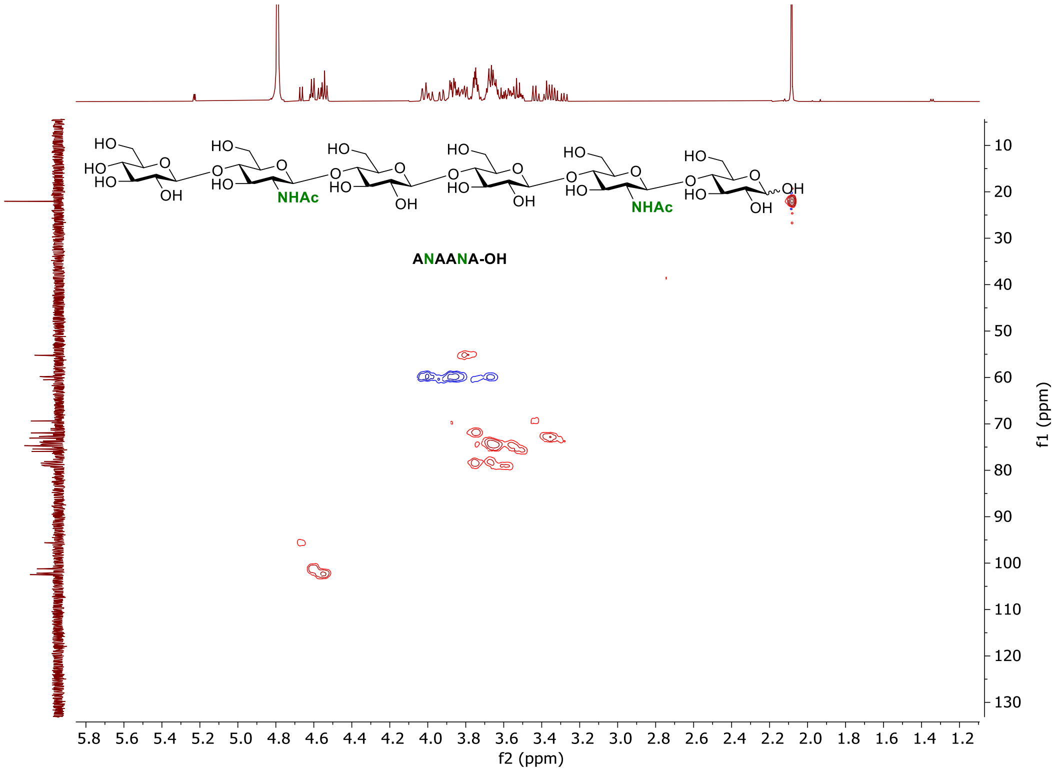

3.5.3 *Synthesis of AAANNN-OH*

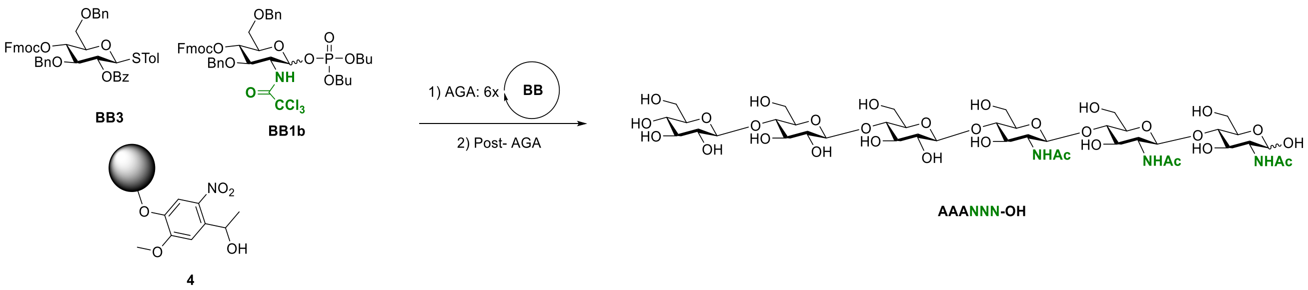

Step		Modules	Notes
AGA		A	
	3 x BB1b	B, C*, D, E	C*:(BB1b, -35° for 5 min, -10° for 40 min)
	3 x BB3	B, C, D, E	C:(BB3, -20° for 5 min, 0° for 20 min)
Post-AGA		F, G, H, I	I: (Method D, t_R = 34.3 and 35.9 min)

Automated synthesis, global deprotection, and purification afforded **AAANNN-OH** as white solid (1.92 mg, 7% overall yield).

Analytical data for **AAANNN-OH**: ^{1}H NMR (700 MHz, D_2O) δ 5.21 – 5.18 (m, 1H), 4.70 (d, *J* = 7.9 Hz, 1H), 4.64 – 4.57 (m, 2H), 4.57 – 4.49 (m, 3H), 4.02 – 3.60 (m, 30H), 3.60 – 3.28 (m, 7H), 2.07 (s, 6H), 2.05 (s, 3H).; ^{13}C NMR (176 MHz, D_2O) δ 175.04, 102.56, 102.34, 94.36 (β-C1), 90.67 (α-C1), 78.39, 78.25, 75.98, 75.48, 74.82, 74.51, 74.04, 73.99, 73.15, 72.93, 71.96, 69.45, 69.24, 59.90, 55.27, 53.63, 22.10, 21.87.; m/z (HRMS+) 1136.419 [M + Na]$^+$ ($C_{42}H_{71}N_3O_{31}Na$ requires 1136.397).

RP-HPLC of AAANNN-OH (ELSD trace, Method D, t_R = 34.3 and 35.9 min)

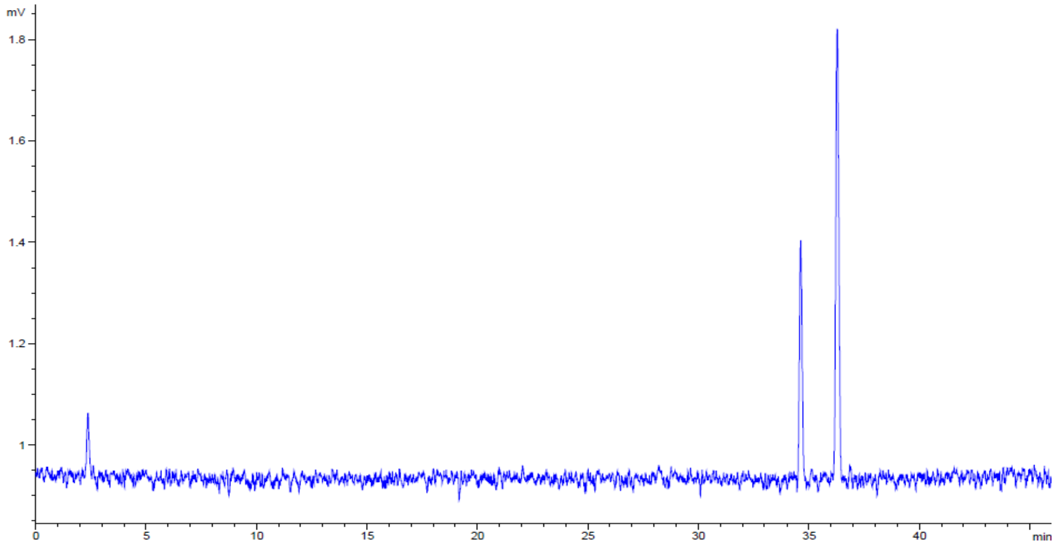

^{1}H NMR of AAANNN-OH (700 MHz, D_2O)

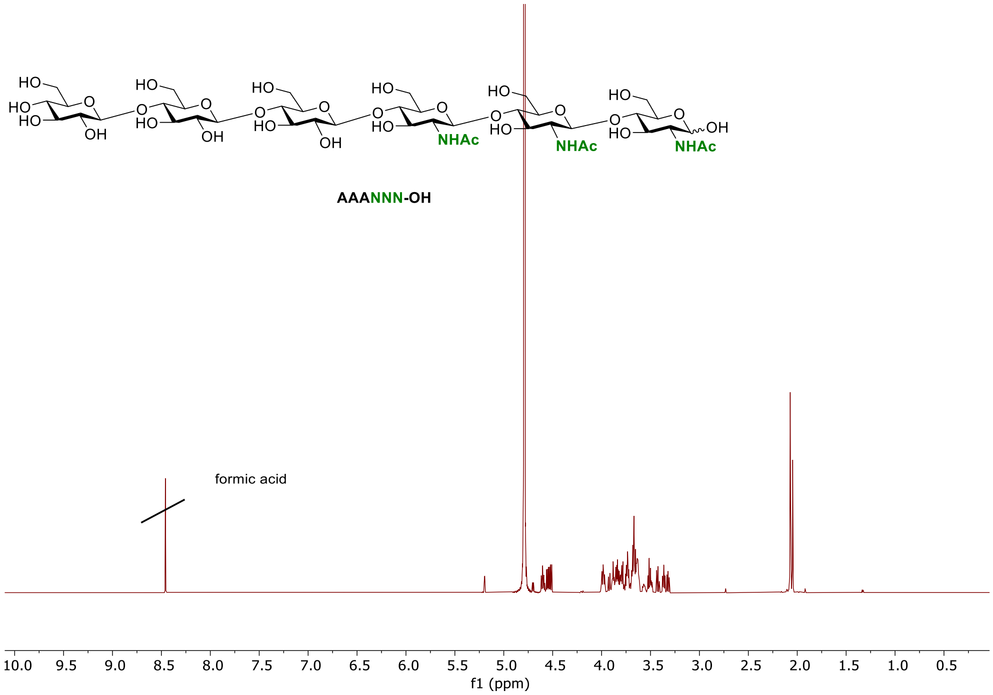

^{13}C NMR of AAANNN-OH (176 MHz, D_2O)

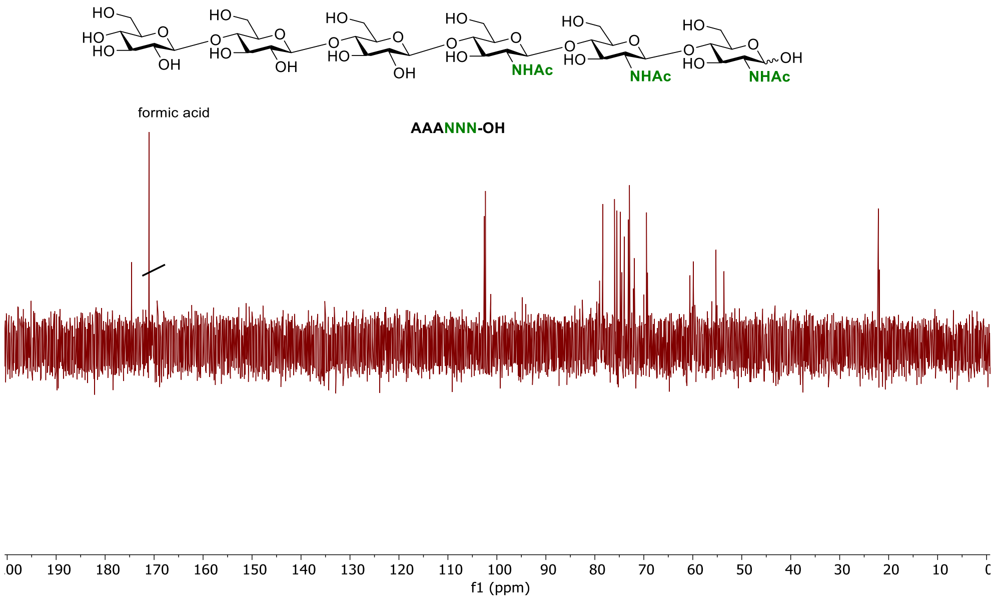

HSQC NMR of AAANNN-OH (D_2O)

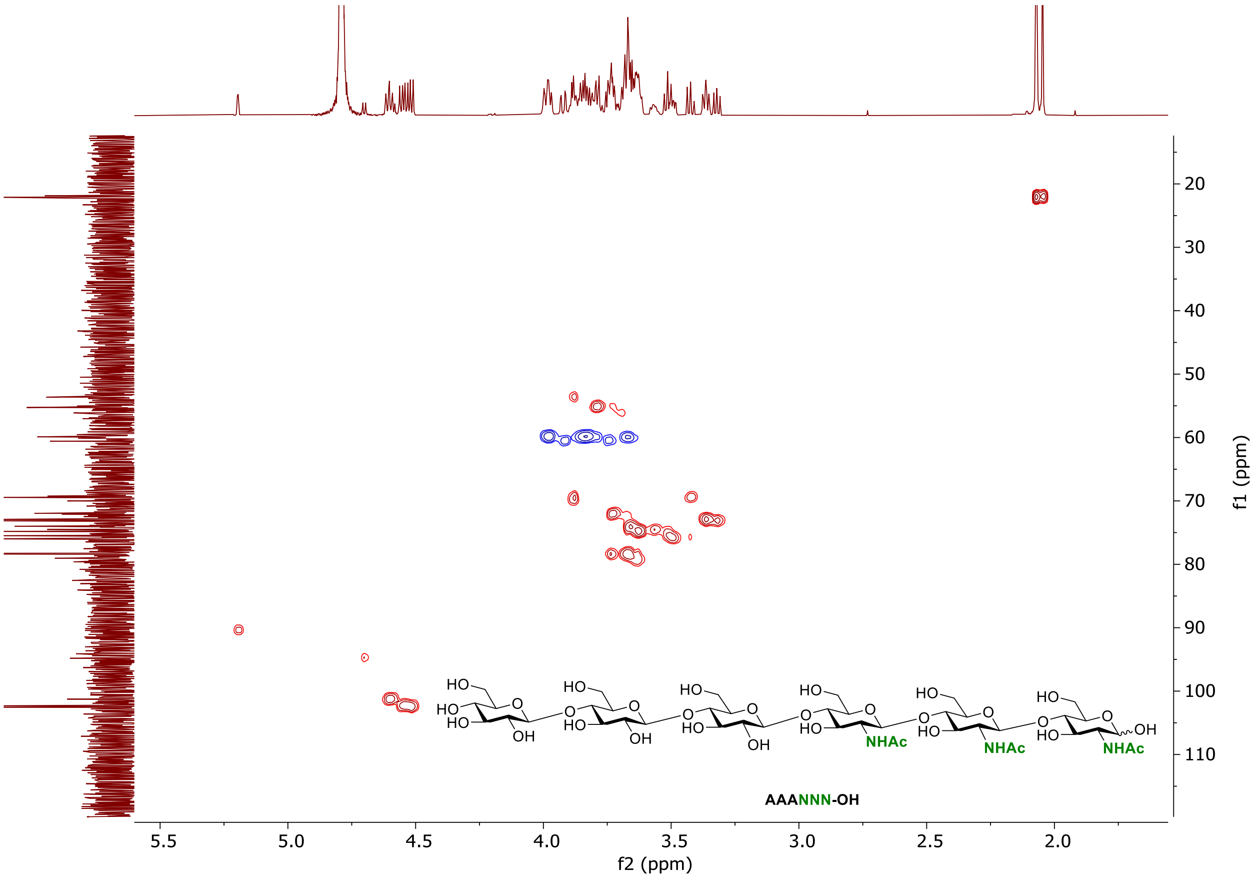

3.5.4 *Synthesis of NANNAN-OH*

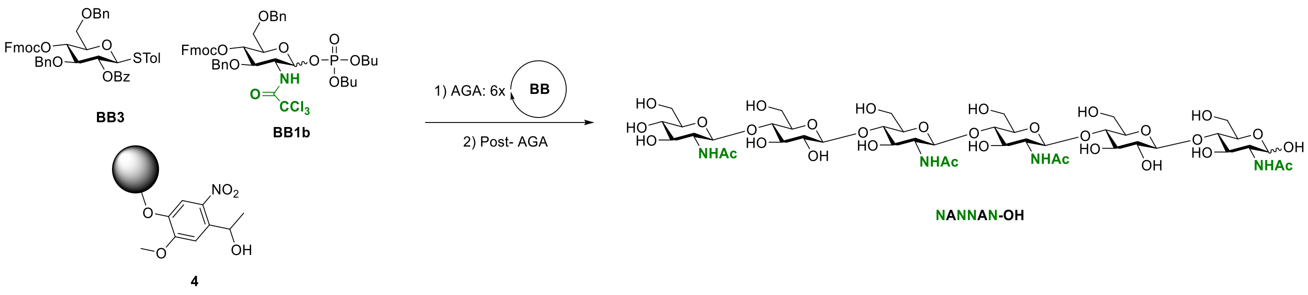

Step		Modules	Notes
AGA		**A**	
	**BB1b**	**B**, **C***, **D**, **E**	**C***:(**BB1b**, -35° for 5 min, -10° for 40 min)
	**BB3**	**B**, **C**, **D**, **E**	**C**:(**BB3**, -20° for 5 min, 0° for 20 min)
	**2 x BB1b**	**B**, **C***, **D**, **E**	**C***:(**BB1b**, -35° for 5 min, -10° for 40 min)
	**BB3**	**B**, **C**, **D**, **E**	**C**:(**BB3**, -20° for 5 min, 0° for 20 min)
	**BB1b**	**B**, **C***, **D**, **E**	**C***:(**BB1b**, -35° for 5 min, -10° for 40 min)
Post-AGA		**F**, **G**, **H**, **I**	**I**: (Method D, t_R = 33.2 and 35.3 min)

Automated synthesis, global deprotection, and purification afforded **NANNAN-OH** as white solid (1.87 mg, 13% overall yield).

Analytical data for **NANNAN-OH**: ^{1}H NMR (600 MHz, D_2O) δ 5.22 (d, *J* = 3.1 Hz, 0.59H, α-H1), 4.73 (d, *J* = 8.0 Hz, 0.41H, β-H1), 4.65 – 4.51 (m, 5H), 4.03 – 3.45 (m, 38H), 3.39 – 3.31 (m, 2H), 2.08 (s, 3H), 2.08 (s, 3H), 2.07 (s, 3H), 2.06 (s, 3H); ^{13}C NMR (151 MHz, D_2O) δ 174.52, 174.40, 102.14, 101.36, 101.26, 101.15, 94.78 (β-C1), 90.47 (α-C1), 84.26, 79.05, 78.97, 78.83, 78.74, 78.42, 78.17, 75.81, 74.73, 74.51, 74.42, 74.07, 74.01, 73.37, 72.73, 72.69, 72.27, 72.02, 71.87, 70.22, 69.66, 69.09, 60.47, 59.93, 59.80, 56.30, 55.53, 55.22, 54.97, 53.75, 22.10, 22.05, 22.03, 21.81; m/z (HRMS+) 1177.421 $[M + Na]^+$ ($C_{44}H_{74}N_4O_{31}Na$ requires 1177.424).

RP-HPLC of NANNAN-OH (ELSD trace, Method D, t_R = 33.2 and 35.3 min)

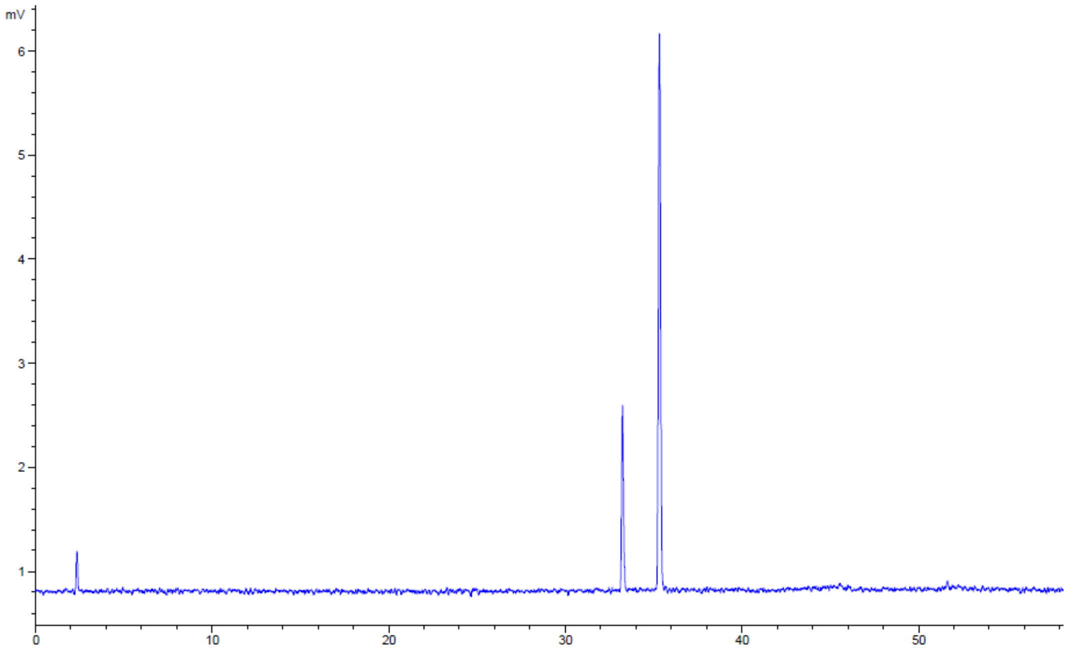

^{1}H NMR of NANNAN-OH (600 MHz, D_2O)

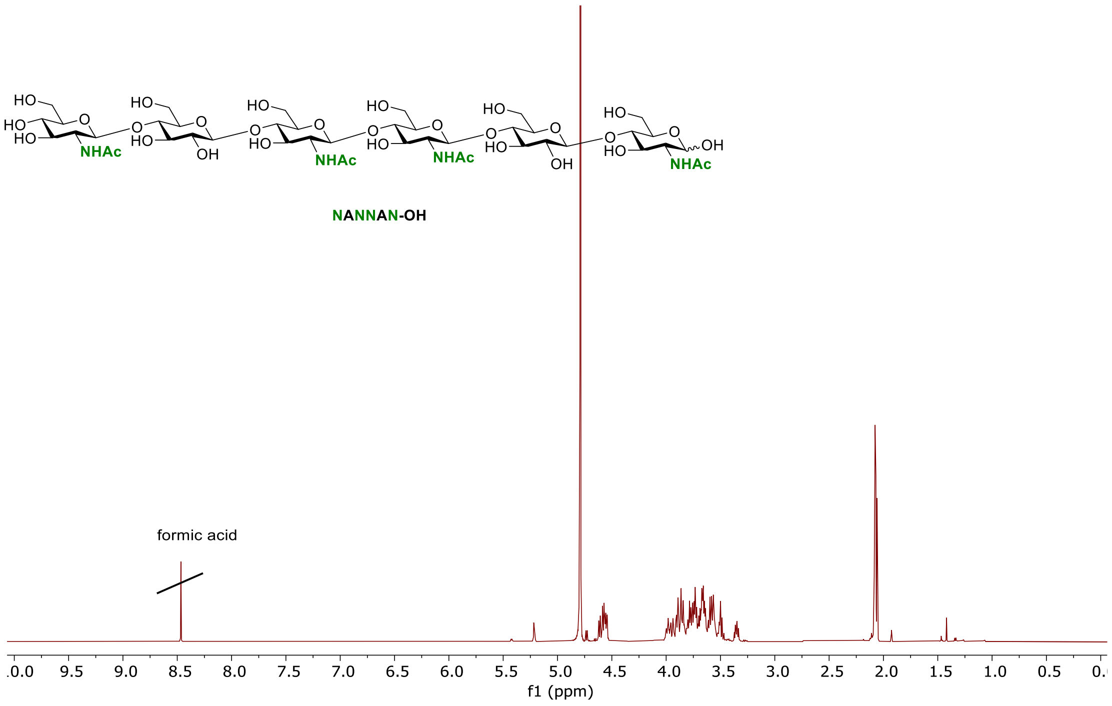

^{13}C NMR of NANNAN-OH (151 MHz, D_2O)

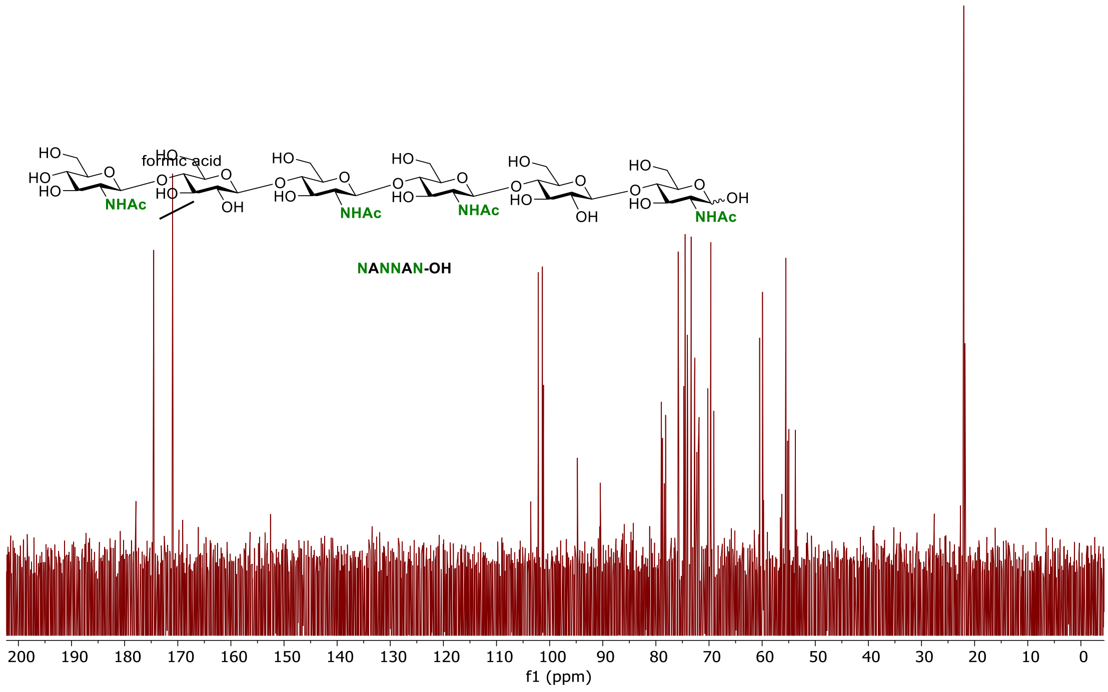

HSQC NMR of NANNAN-OH (D_2O)

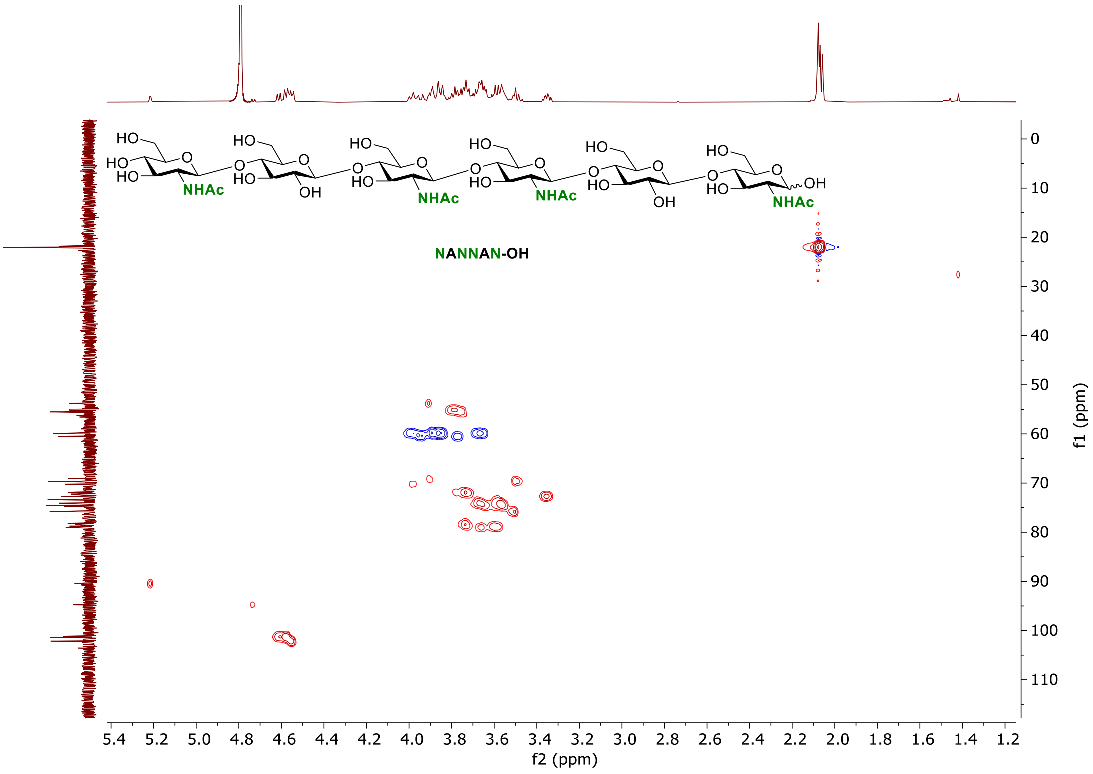

3.5.5 Synthesis of NNAANN-OH

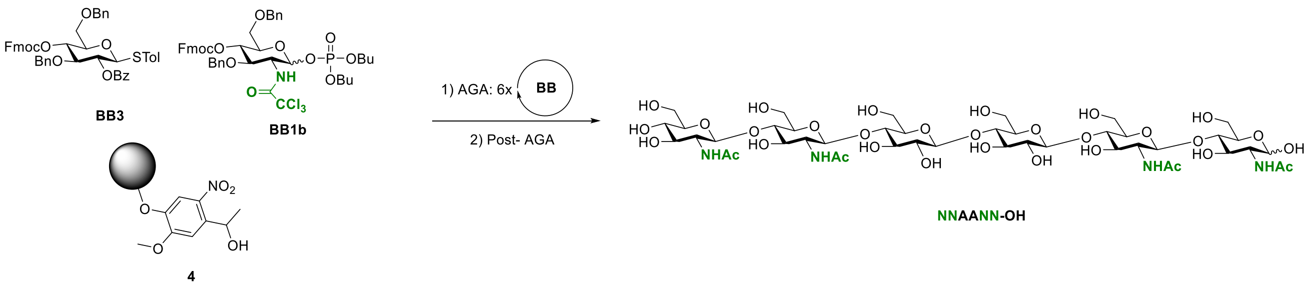

Step		Modules	Notes
AGA		**A**	
	**2 x BB1b**	**B, C*, D, E**	**C***:(**BB1b**, -35° for 5 min, -10° for 40 min)
	**2 x BB3**	**B, C, D, E**	**C**:(**BB3**, -20° for 5 min, 0° for 20 min)
	**2 x BB1b**	**B, C*, D, E**	**C***:(**BB1b**, -35° for 5 min, -10° for 40 min)
Post-AGA		**F, G, H, I**	**I**: (Method D, t_R = 32.8 and 34.4 min)

Automated synthesis, global deprotection, and purification afforded **NNAANN-OH** as white solid (3.25 mg, 23% overall yield).

Analytical data for **NNAANN-OH**: ^{1}H NMR (600 MHz, D_2O) δ 5.21 (d, *J* = 2.7 Hz, 0.57H, α-H1), 4.71 (d, *J* = 7.5 Hz, 0.43H, β-H1), 4.65 – 4.50 (m, 5H), 4.05 – 3.44 (m, 37H), 3.37 (d, *J* = 9.7 Hz, 1H), 3.34 (d, *J* = 9.7 Hz, 1H), 2.08 (s, 5H), 2.07 (s, 3H), 2.05 (s, 3H); ^{13}C NMR (151 MHz, D_2O) δ 174.66, 174.55, 174.51, 174.40, 170.97, 102.28, 102.17, 101.41, 101.27, 101.17, 94.74 (β-C1), 90.36 (α-C1), 79.65, 79.20, 79.14, 78.80, 78.34, 78.08, 75.83, 74.77, 74.70, 74.50, 74.41, 74.00, 73.90, 73.36, 72.86, 72.69, 72.42, 72.07, 71.91, 69.91, 69.62, 69.18, 60.45, 60.06, 59.91, 59.76, 56.02, 55.50, 55.21, 54.95, 53.56, 22.09, 22.03, 21.80; m/z (HRMS+) 1177.429 $[M + Na]^+$ ($C_{44}H_{74}N_4O_{31}Na$ requires 1177.424).

RP-HPLC of NNAANN-OH (ELSD trace, Method D, t_R = 32.8 and 34.4 min)

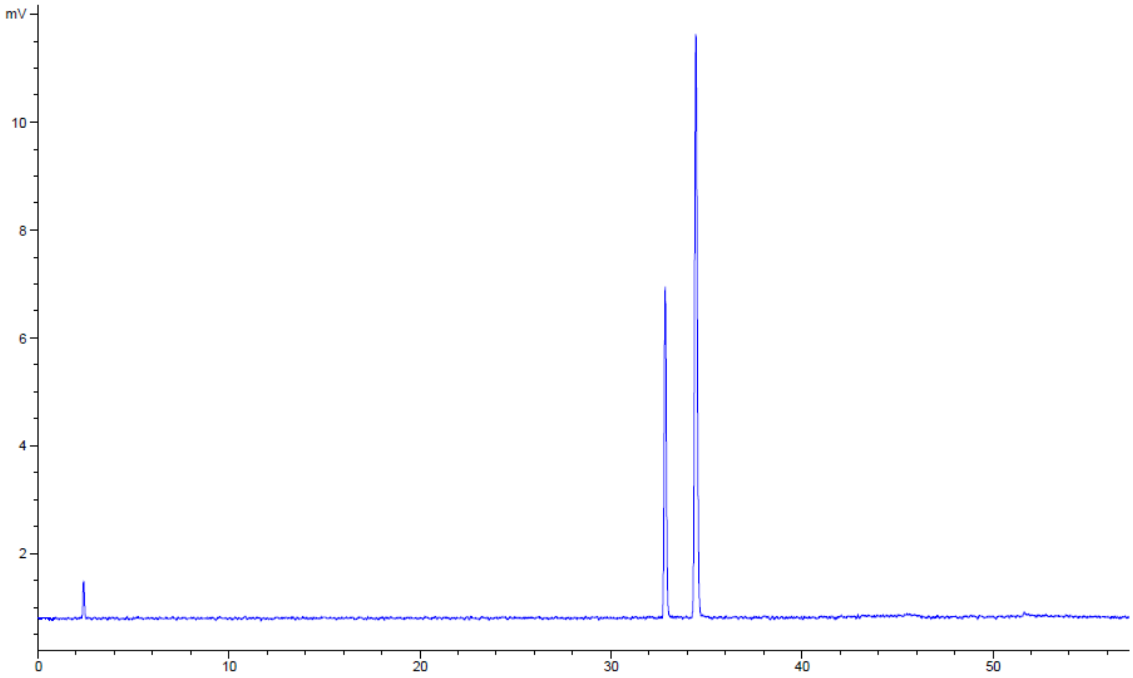

^{1}H NMR of NNAANN-OH (600 MHz, D_2O)

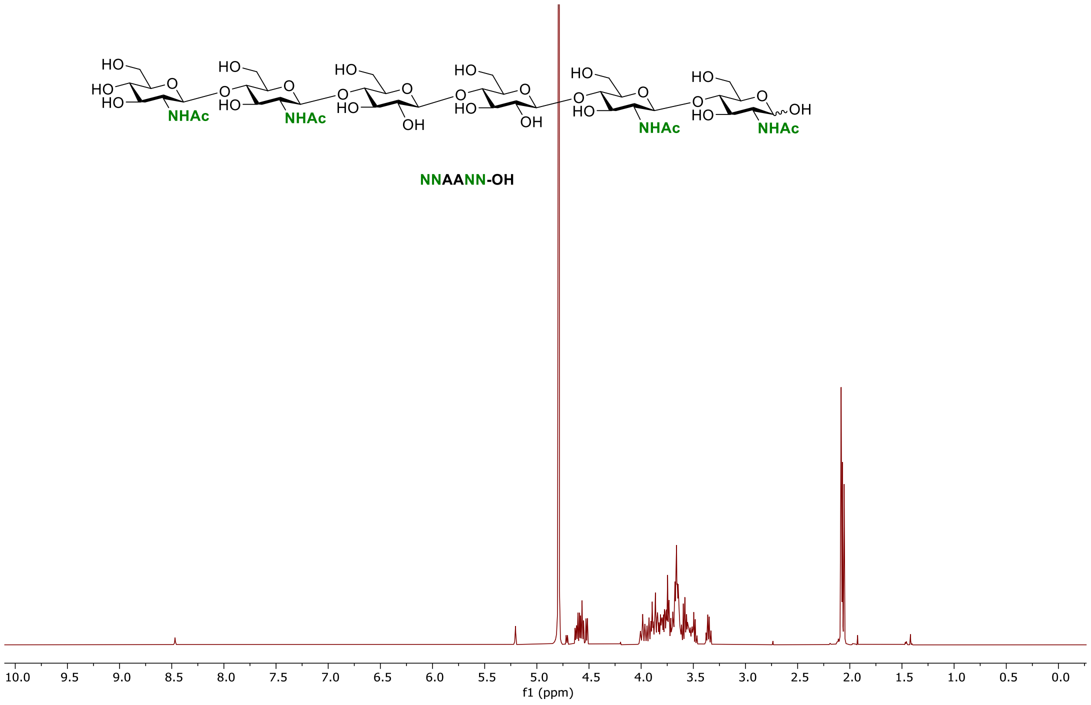

^{13}C NMR of NNAANN-OH (151 MHz, D_2O)

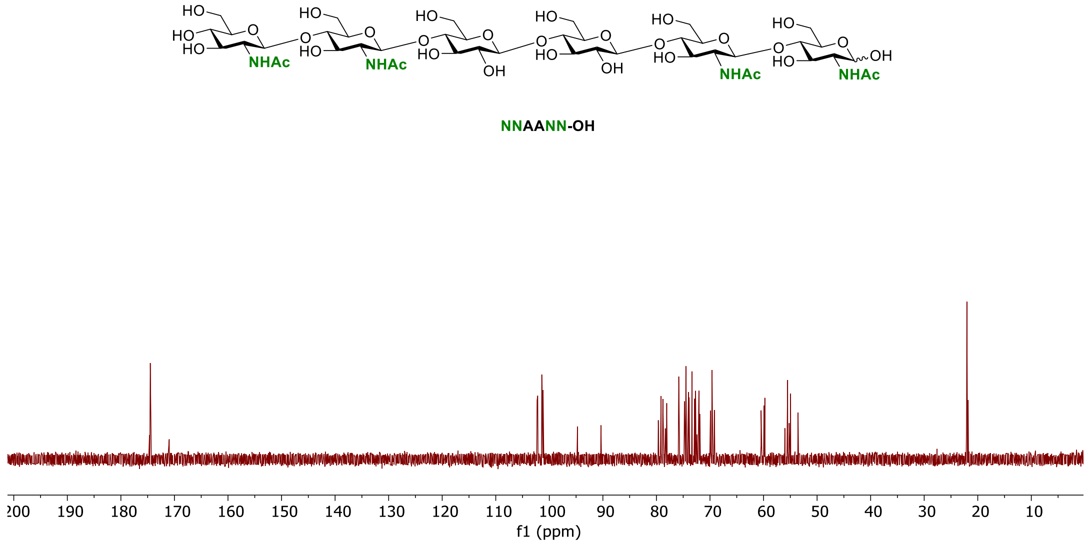

HSQC NMR of NNAANN-OH (D_2O)

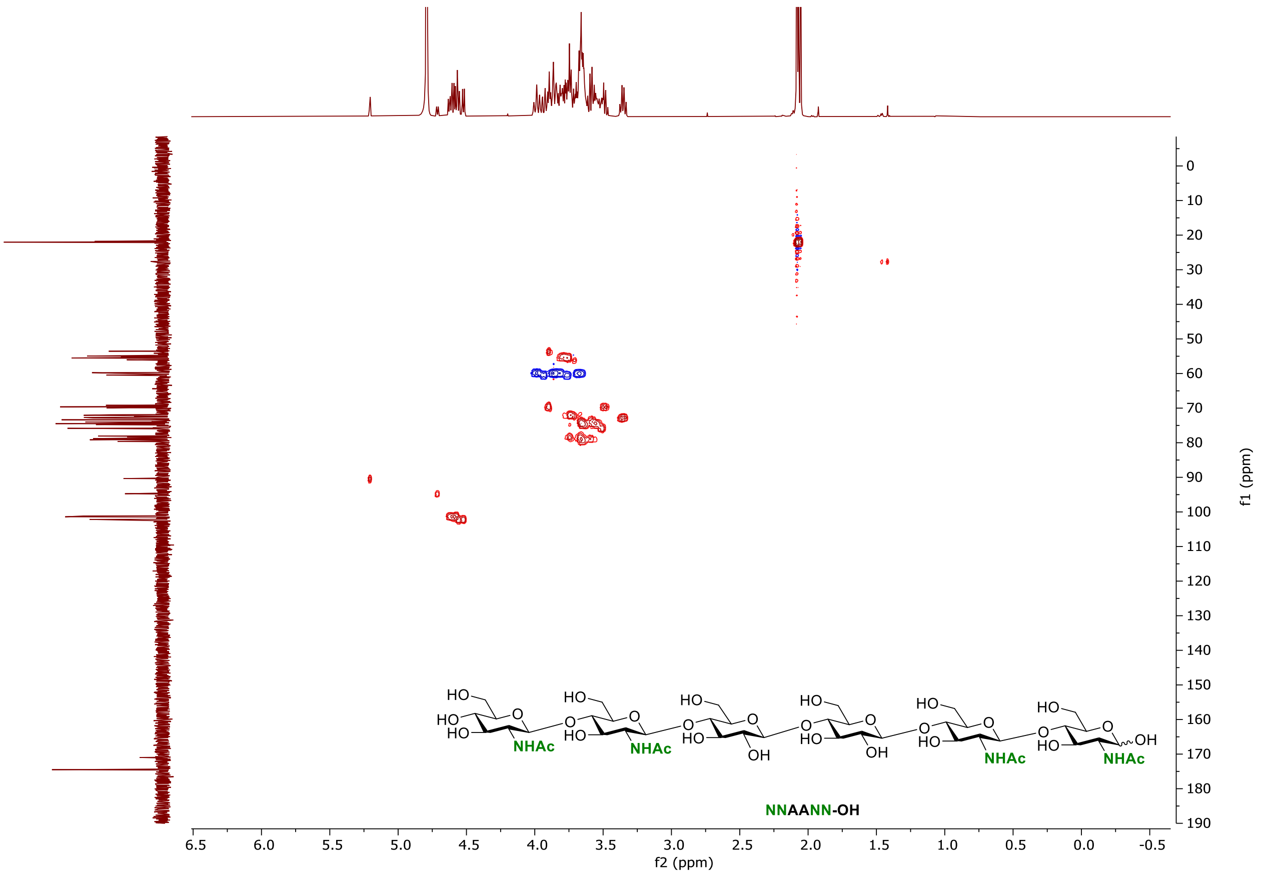

3.5.6 Synthesis of KKKNNN-OH

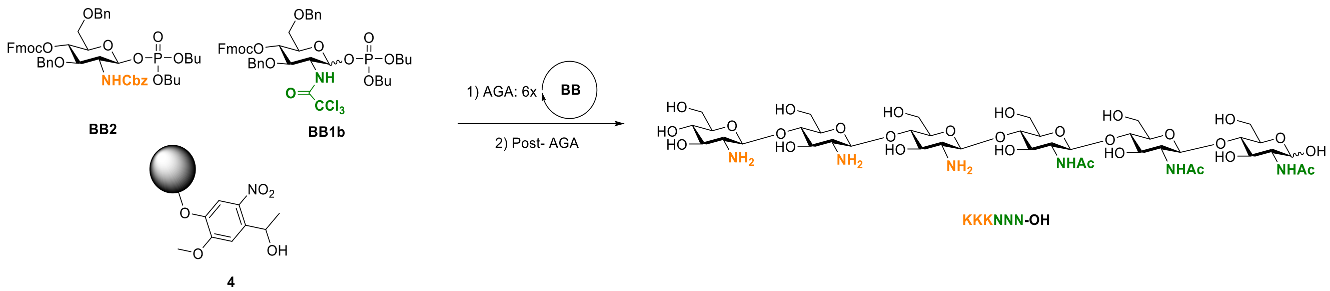

Step		Modules	Notes
AGA		**A**	
	**3 x BB1b**	**B**, **C***, **D**, **E**	**C***:(**BB1b**, -35° for 5 min, -10° for 40 min)
	**3 x BB2**	**B**, **C***, **D**, **E**	**C***:(**BB2**, -35° for 5 min, -10° for 40 min)
Post-AGA		**G**, **I1**, **H**, **I2**	**I1**: (Method B, t_R = 41.2 min),<br>**I2**: (Method D, t_R = 14.4 and 14.6 min)

Automated synthesis, global deprotection, and purification afforded **KKKNNN-OH** as white solid (2.3 mg, 19 % overall yield).

Analytical data for **KKKNNN-OH**: ^{1}H NMR (600 MHz, D_2O) δ 5.20 (d, *J* = 2.4 Hz, 0.58H, α-H1), 4.92 – 4.86 (m, 3H), 4.70 (d, *J* = 7.8 Hz, 0.42H, β -H1), 4.61 (m, 2H), 4.06 – 3.45 (m, 39H), 3.24 – 3.12 (m, 3H), 2.24 (s, 1H), 2.09 – 2.06 (m, 6H), 2.05 (d, *J* = 1.5 Hz, 3H).; ^{13}C NMR (151 MHz, D_2O) δ 174.59, 174.52, 101.15, 97.69, 97.47, 97.21, 94.71, 90.38, 79.50, 78.79, 76.34, 76.26, 76.18, 74.73, 74.69, 74.51, 74.22, 72.40, 72.02, 71.68, 71.34, 70.05, 69.93, 69.51, 69.20, 60.21, 60.12, 59.91, 59.85, 56.06, 55.75, 55.73, 55.61, 55.47, 55.04, 53.56, 30.16, 22.09, 22.05, 22.03, 21.80. m/z (HRMS+) 556.2319 $[M+2H]^{+2}$ ($C_{42}H_{72}N_6O_{28}{}^{+2}$ requires 556.2307).

RP-HPLC of KKKNNN-OH (ELSD trace, Method G, t_R = 14.4 and 14.6 min)

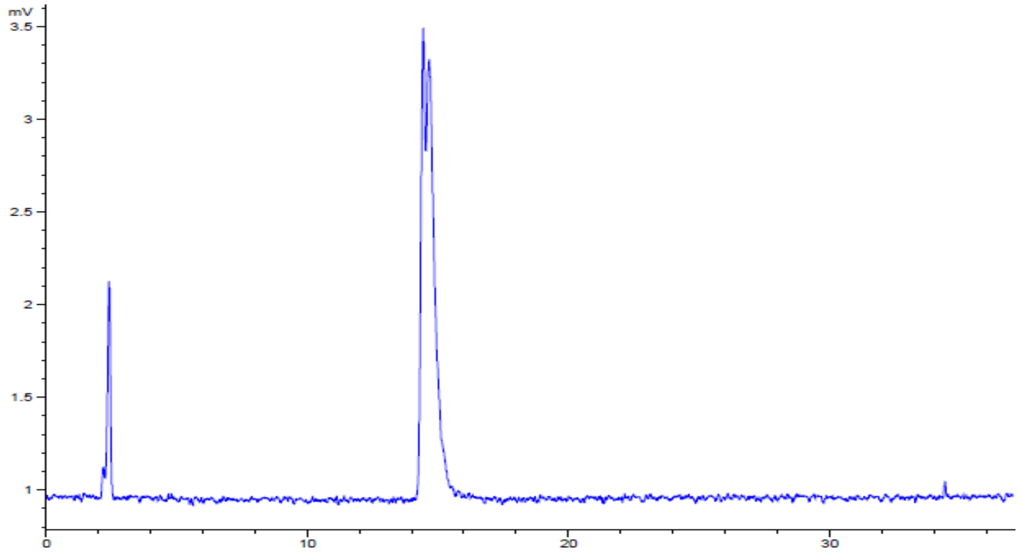

^{1}H NMR of KKKNNN-OH (600 MHz, D_2O)

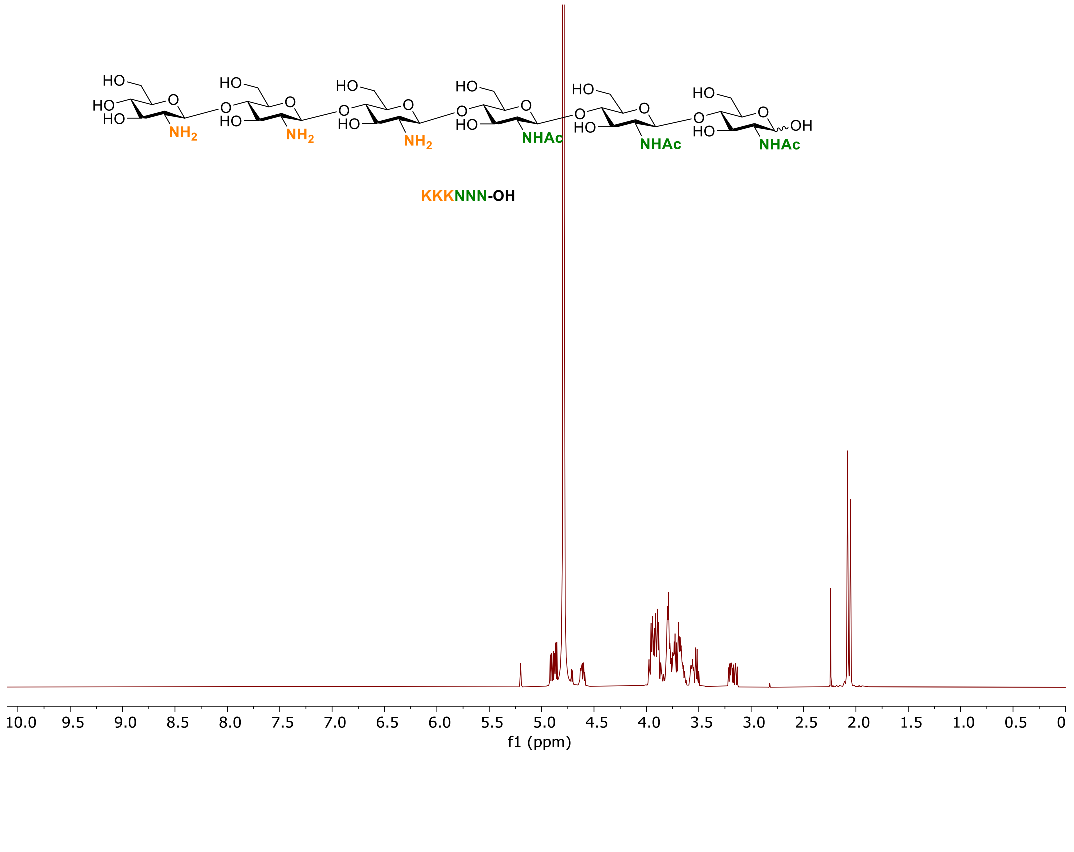

^{13}C NMR of KKKNNN-OH (151 MHz, D_2O)

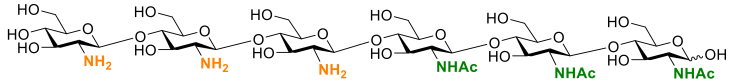

KKKNNN-OH

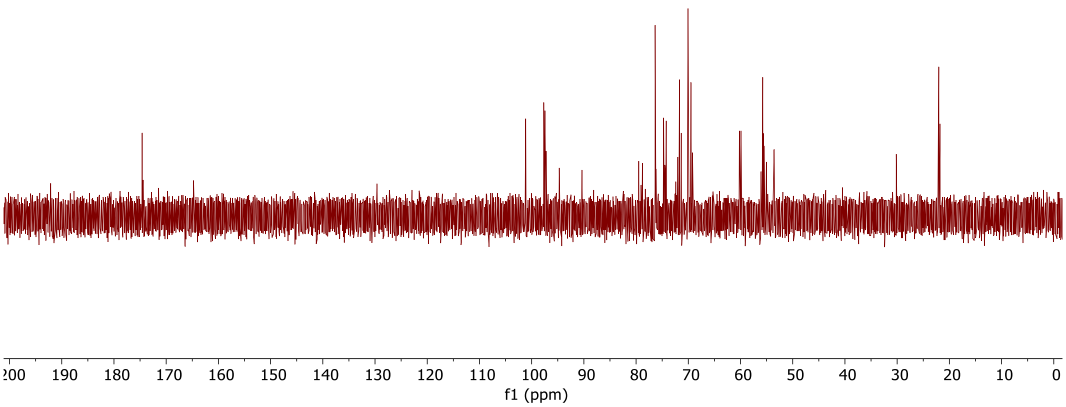

HSQC NMR of KKKNNN-OH (D_2O)

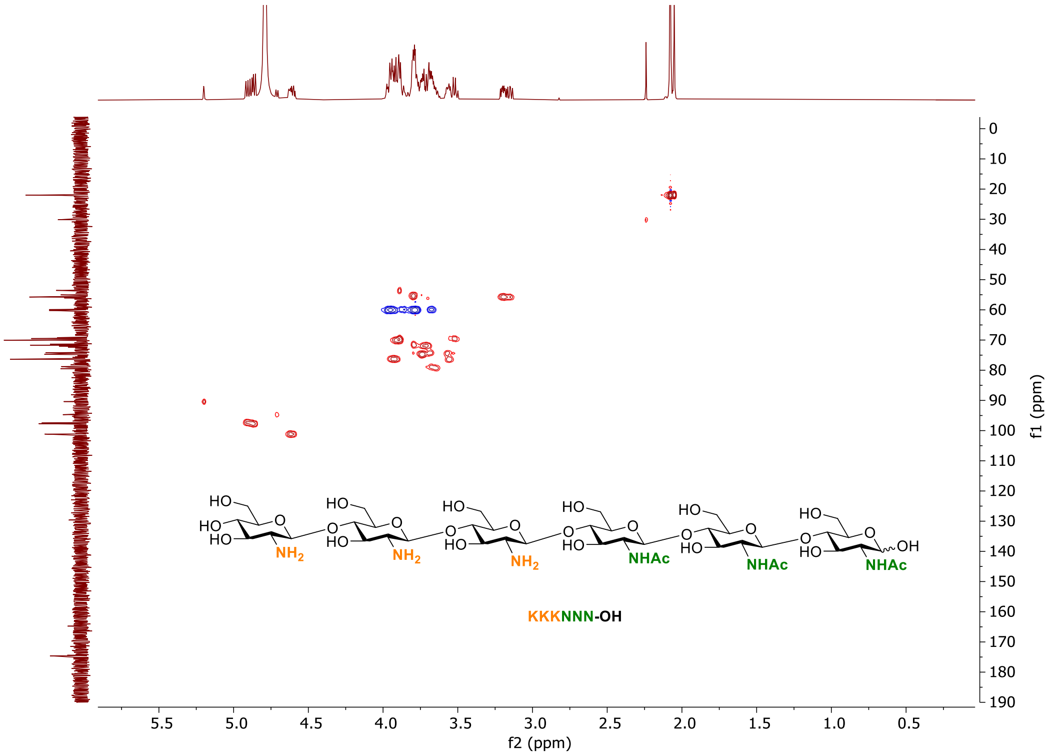

3.5.7 *Synthesis of KNKNKN-OH*

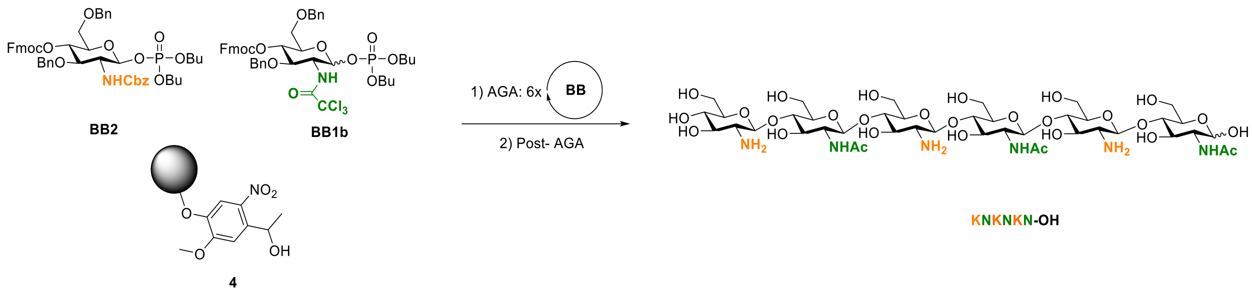

Step		Modules	Notes
		**A**	
AGA	**3 x BB1b**	**B, C*, D, E**	**C***:(**BB1b**, -35° for 5 min, -10° for 40 min)
	**3 x BB2**	**B, C*, D, E**	**C***:(**BB2**, -35° for 5 min, -10° for 40 min)
Post-AGA		**G, I1, H, I2**	**I1**: (Method B, t_R = 43.6 and 44.9 min),<br>**I2**: (Method D, t_R = 15.0 and 15.3 min)

Automated synthesis, global deprotection, and purification afforded **KNKNKN-OH** as white solid (1.3 mg, 11 % overall yield).

Analytical data for **KNKNKN-OH**: ^{1}H NMR (600 MHz, D_2O) δ 5.06 (d, *J* = 2.6 Hz, 0.56H,i α-H1), 4.77 – 4.72 (m, 3H), 4.70 (d, *J* = 12 Hz, 0.44H, β-H1), 4.47 – 4.41 (m, 2H), 3.91 – 3.31 (m, 39H), 3.01 (dt, *J* = 11.0, 8.4 Hz, 3H), 1.93 (s, 6H), 1.91 (s, *J* = 1.6 Hz, 3H).; ^{13}C NMR (151 MHz, d_2o) δ 174.61, 101.24, 97.33, 97.07, 90.52, 76.34, 76.19, 75.02, 74.27, 71.74, 71.25, 70.45, 69.84, 69.44, 60.21, 59.57, 55.65, 55.47, 22.03, 21.82.; m/z (HRMS+) 556.2307 $[M+2H]^{+2}$ ($C_{42}H_{72}N_6O_{28}^{+2}$ requires 556.2307).

RP-HPLC of KNKNKN-OH (ELSD trace, Method G, t_R = 14.4 and 14.6 min)

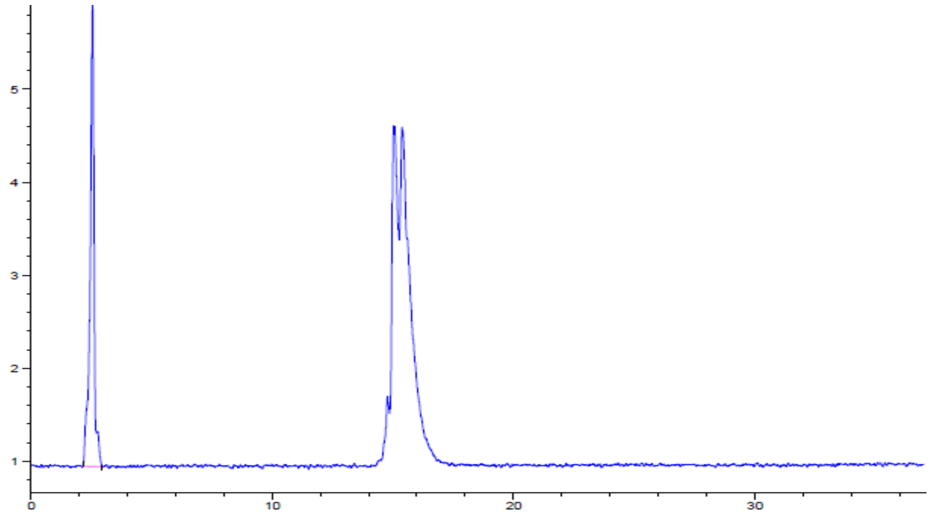

^{1}H NMR of KNKNKN-OH (600 MHz, D_2O)

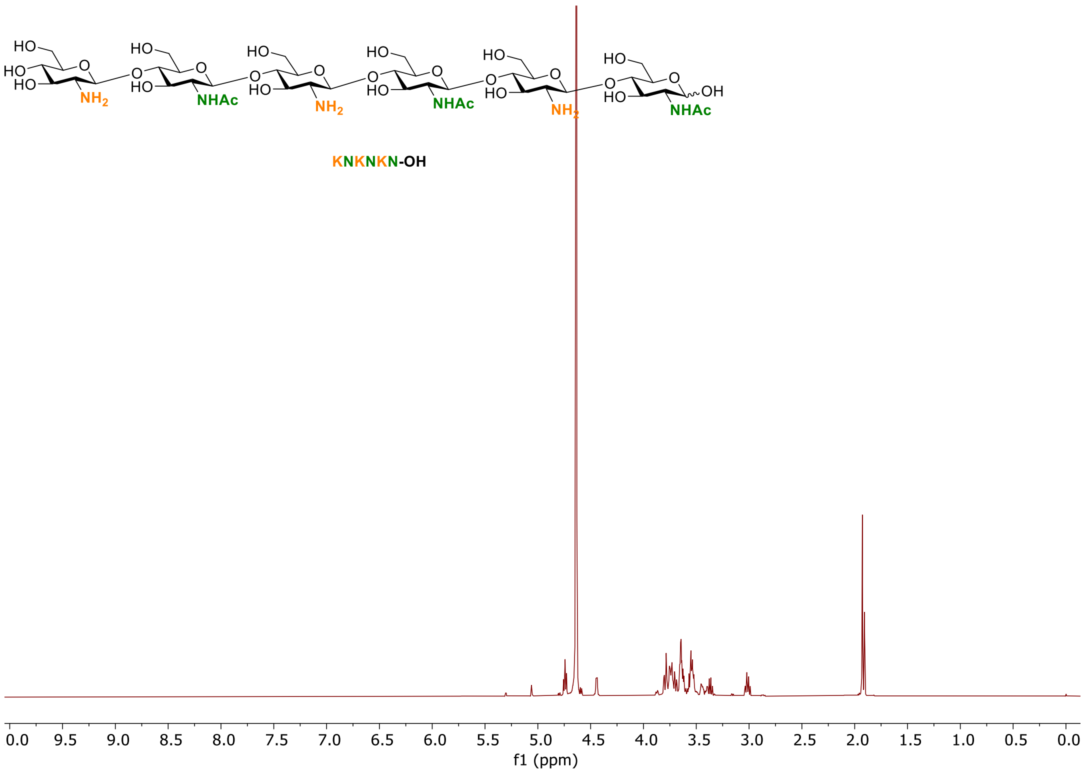

^{13}C NMR of KNKNKN-OH OH (151 MHz, D_2O)

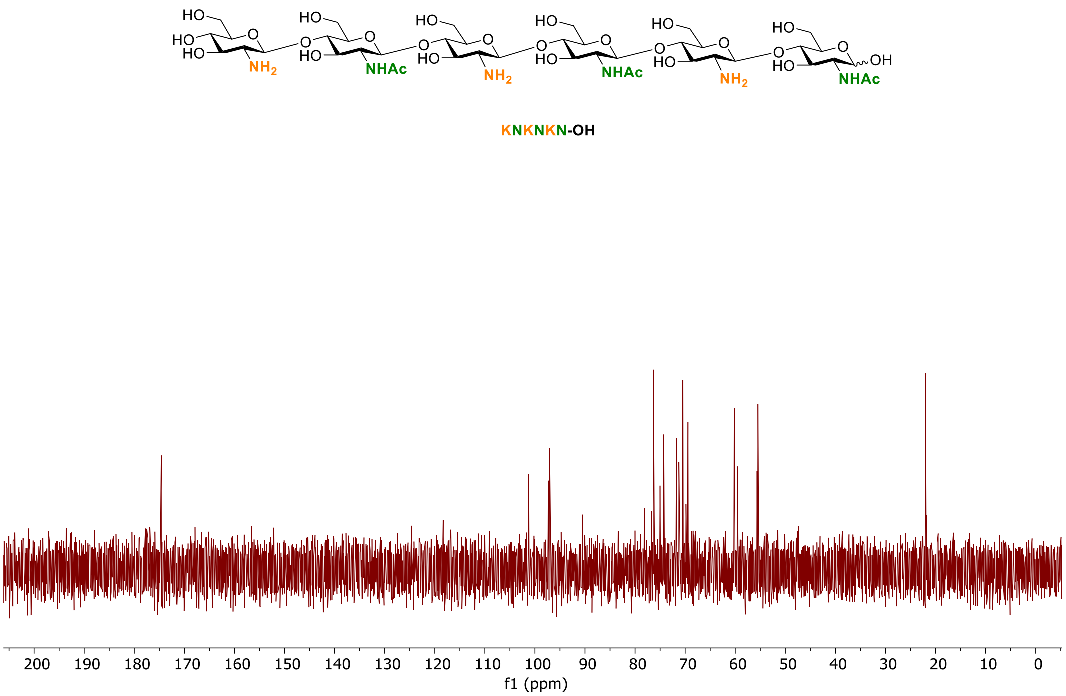

HSQC NMR of KNKNKN-OH (D_2O)

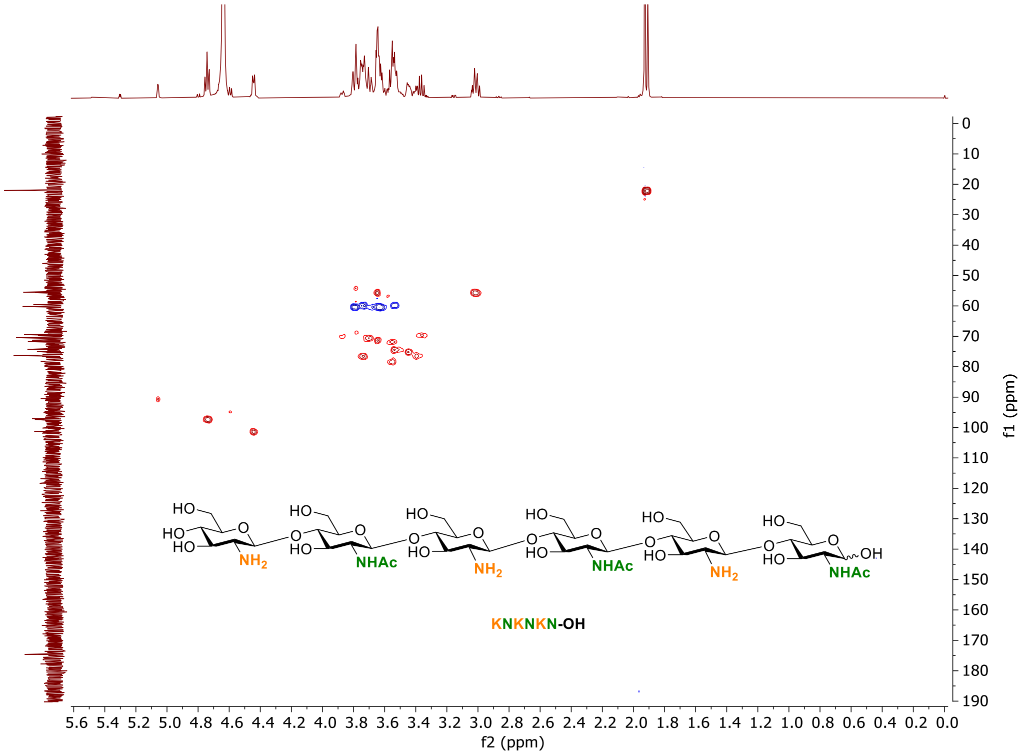

3.5.8 Synthesis of NKNNKN-OH

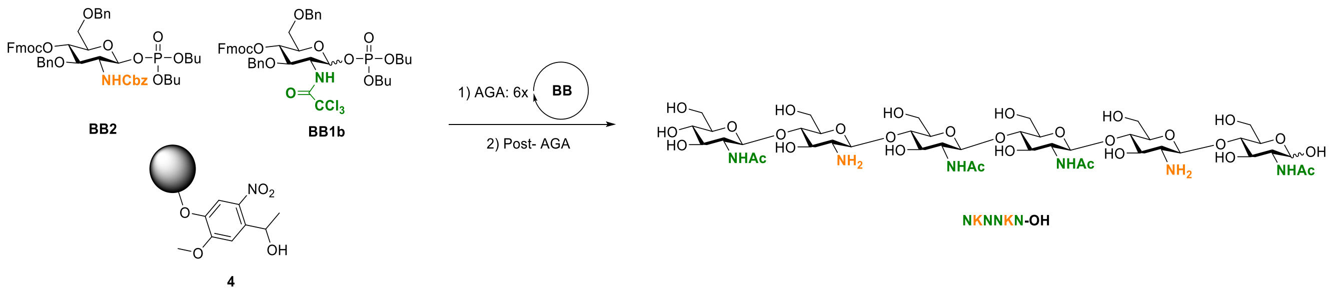

Step		Modules	Notes
AGA		A	
	**BB1b**	**B**, **C***, **D**, **E**	**C***:(**BB1b**, -35° for 5 min, -10° for 40 min)
	**BB2**	**B**, **C***, **D**, **E**	**C***:(**BB2**, -35° for 5 min, -10° for 40 min)
	**2 x BB1b**	**B**, **C***, **D**, **E**	**C***:(**BB1b**, -35° for 5 min, -10° for 40 min)
	**BB2**	**B**, **C***, **D**, **E**	**C***:(**BB2**, -35° for 5 min, -10° for 40 min)
	**BB1b**	**B**, **C***, **D**, **E**	**C***:(**BB1b**, -35° for 5 min, -10° for 40 min)
Post-AGA		**G**, **I1**, **H**, **I2**	**I1**: (Method B, t_R = 41.8 min),<br>**I2**: (Method C, t_R = 22.6 and 23.6 min)

Automated synthesis, global deprotection, and purification afforded **NKNNKN-OH** as white solid (3.7 mg, 27 % overall yield).

Analytical data for **NKNNKN-OH**: ^{1}H NMR (600 MHz, D_2O) δ 5.21 (d, *J* = 2.5 Hz, 0.55H, α-H1), 4.90 (d, *J* = 2.3 Hz, 1H), 4.89 (d, *J* = 2.3 Hz, 1H), 4.75 (d, *J* = 9.4, 0.45H, β-H1), 4.63 – 4.60 (m, 1H), 4.60 – 4.54 (m, 2H), 4.14 – 3.35 (m, 38H), 3.23 – 3.11 (m, 2H), 2.07 (d, *J* = 4.4 Hz, 9H), 2.05 (d, *J* = 1.6 Hz, 3H).; ^{13}C NMR (151 MHz, D_2O) δ 174.58, 174.55, 174.45, 101.45, 101.21, 97.07, 94.70, 90.51, 79.07, 78.48, 78.27, 76.80, 76.26, 75.81, 74.99, 74.37, 74.26, 73.26, 71.95, 71.71, 71.29, 70.52, 70.46, 69.83, 69.71, 68.64, 60.49, 60.28, 60.14, 59.89, 59.64, 59.60, 55.49, 55.38, 54.97, 53.90, 22.10, 22.05, 22.01, 21.81.; m/z (HRMS+) 577.2422 $[M+2H]^{+2}$ ($C_{42}H_{72}N_6O_{29}^{+2}$ requires 556.2401).

RP-HPLC of NKNNKN-OH (ELSD trace, Method D, t_R = 23.2 and 24.1 min)

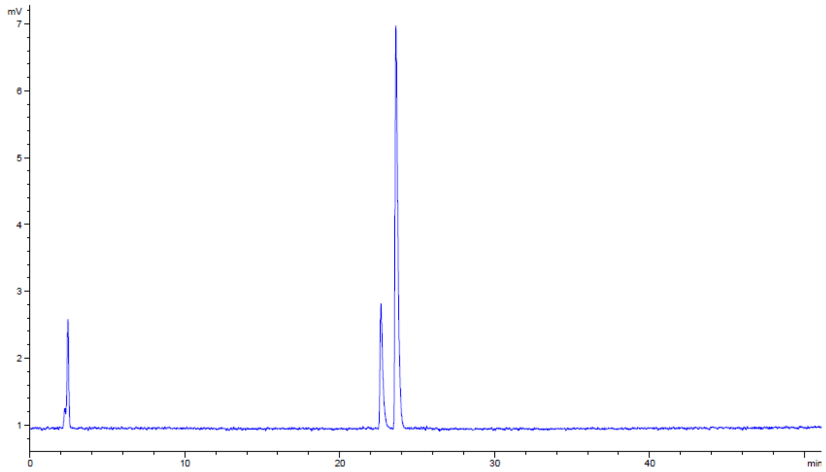

^{1}H NMR of NKNNKN-OH (600 MHz, D_2O)

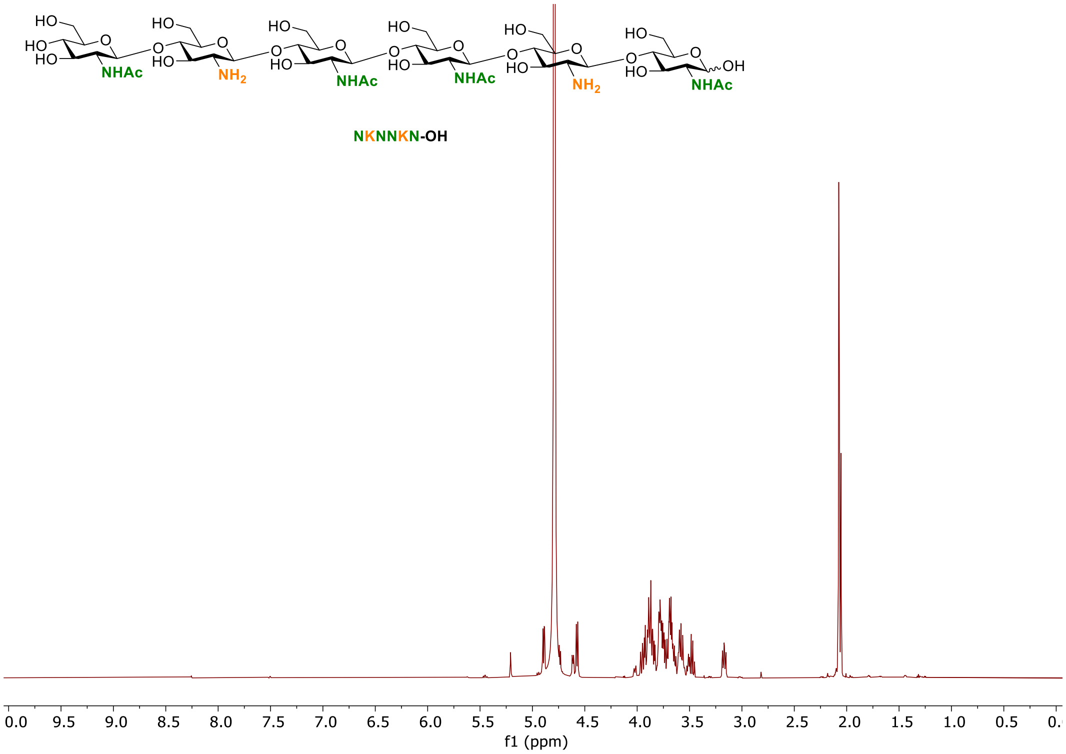

^{13}C NMR of NKNNKN-OH (151 MHz, D_2O)

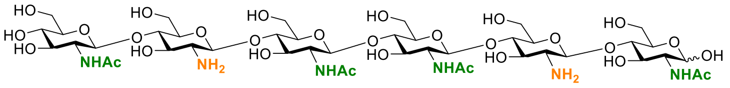

NKNNKN-OH

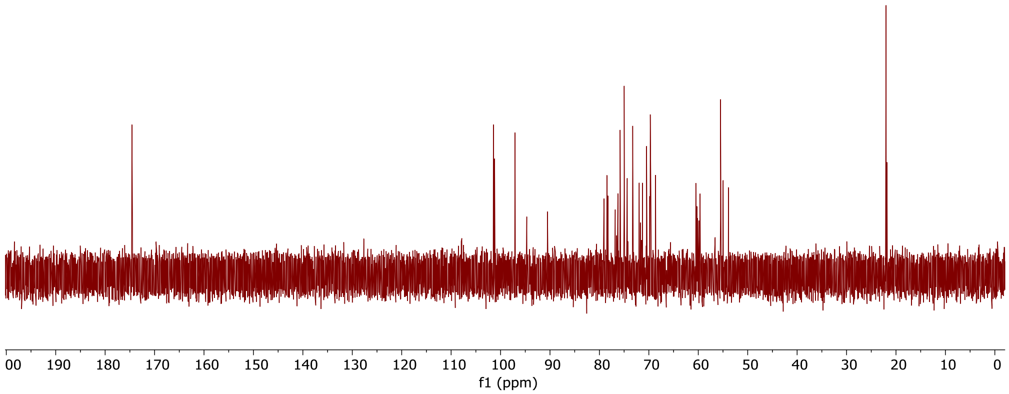

HSQC NMR of NKNNKN-OH (D_2O)

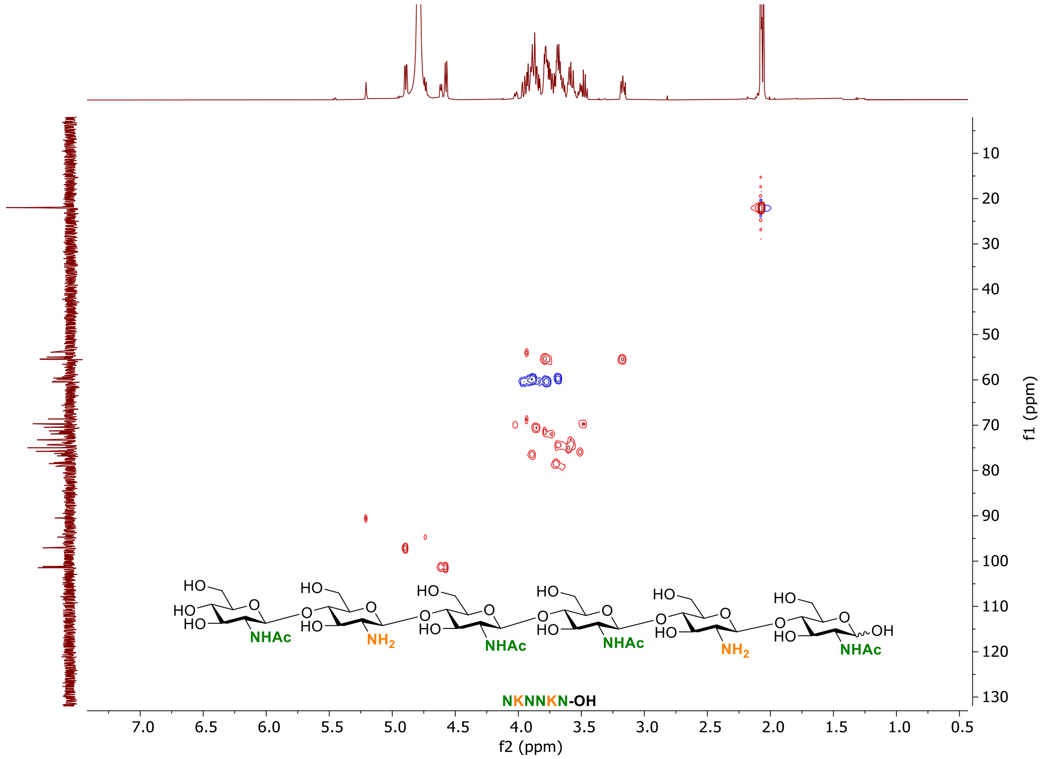

3.5.9 *Synthesis of NNKKNN-OH*

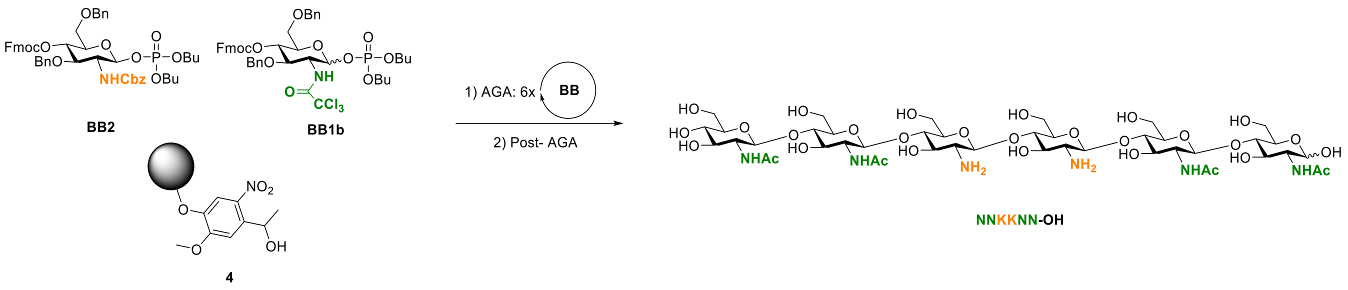

Step		Modules	Notes
AGA		**A**	
	**2 x BB1b**	**B**, **C***, **D**, **E**	**C***:(**BB1b**, -35° for 5 min, -10° for 40 min)
	**2 x BB2**	**B**, **C***, **D**, **E**	**C***:(**BB2**, -35° for 5 min, -10° for 40 min)
	**2 x BB1b**	**B**, **C***, **D**, **E**	**C***:(**BB1b**, -35° for 5 min, -10° for 40 min)
Post-AGA		**G**, **I1**, **H**, **I2**	**I1**: (Method B, t_R = 42.3 min), **I2**: (Method D, t_R = 22.3 and 23.3 min)

Automated synthesis, global deprotection, and purification afforded **NNKKNN-OH** as white solid (1.56 mg, 11% overall yield).

Analytical data for **NNKKNN-OH**: ^{1}H NMR (600 MHz, D_2O) δ 5.21 (d, *J* = 2.5 Hz, 0.53H, α-H1), 4.71 (d, *J* = 7.8 Hz, 0.47H, β-H1), 4.67 – 4.53 (m, 5H), 3.99 – 3.44 (m, 39H), 2.83 (bs, 2H), 2.17 – 2.07 (m, 9H), 2.06 (s, 3H); ^{13}C NMR (151 MHz, D_2O) δ 174.51, 101.41, 101.26, 101.24, 101.22, 94.73 (β-C1), 90.37 (α-C1), 79.63, 79.21, 78.94 75.83, 74.80, 74.72, 74.50, 74.41, 73.36, 72.42, 72.07, 71.78, 69.91, 69.62, 69.18, 60.45, 59.92, 56.16, 56.03, 55.50, 55.27, 54.97, 53.56, 22.04, 21.79; m/z (HRMS+) 1175.460 $[M + Na]^+$ ($C_{44}H_{76}N_6O_{29}Na$ requires 1175.455).

RP-HPLC of NNKKNN-OH (ELSD trace, Method D, t_R = 22.3 and 23.3 min)

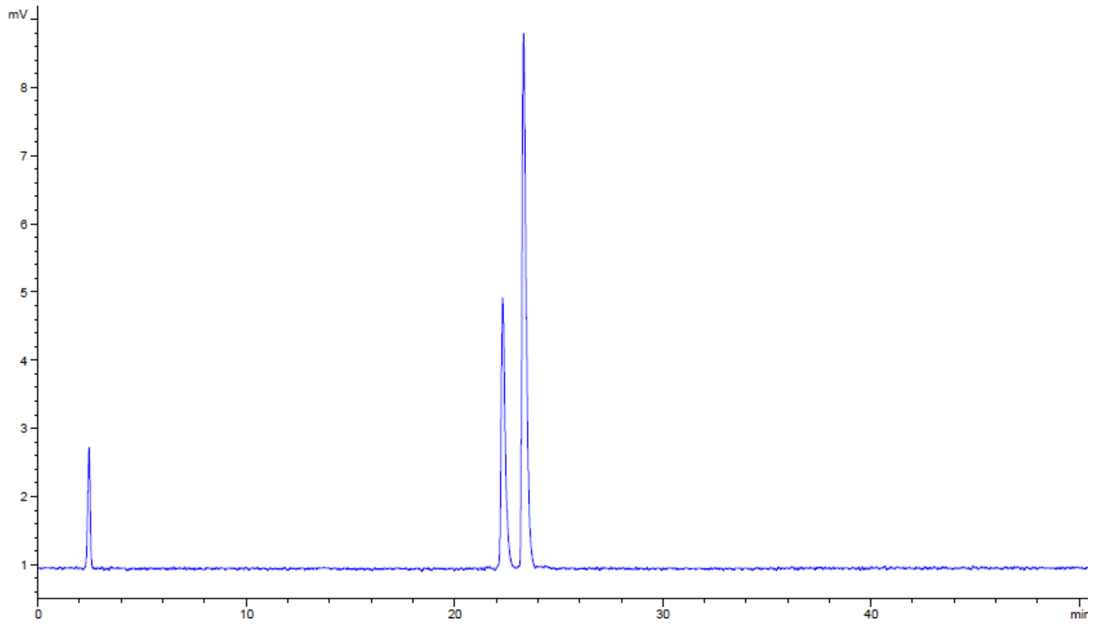

^{1}H NMR of NNKKNN-OH (600 MHz, D_2O)

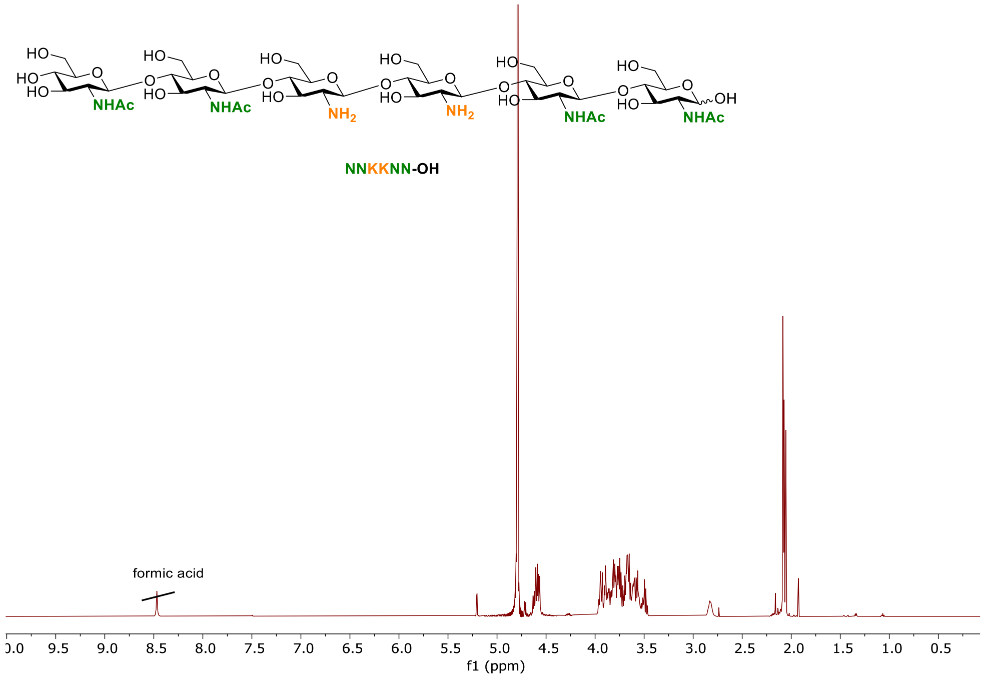

^{13}C NMR of NNKKNN-OH (151 MHz, D_2O)

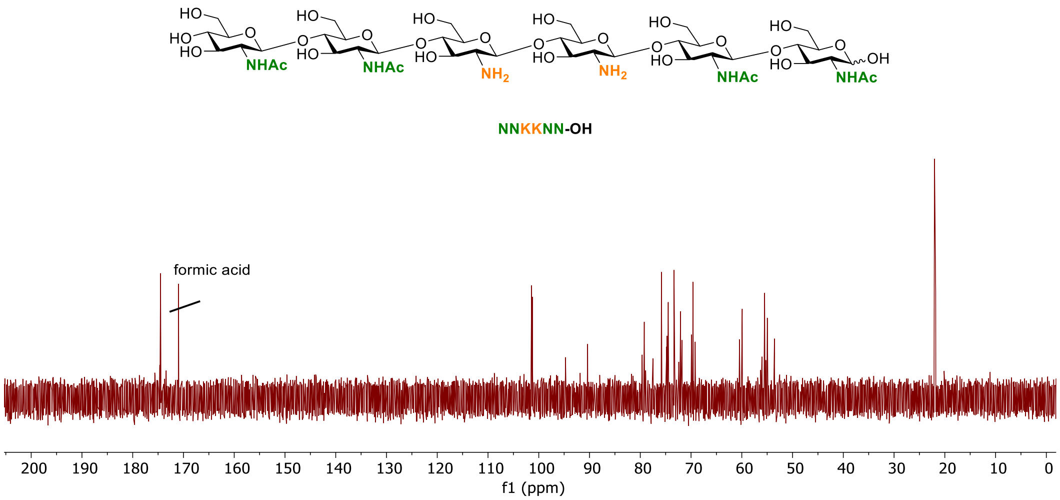

HSQC NMR of NNKKNN-OH (D_2O)

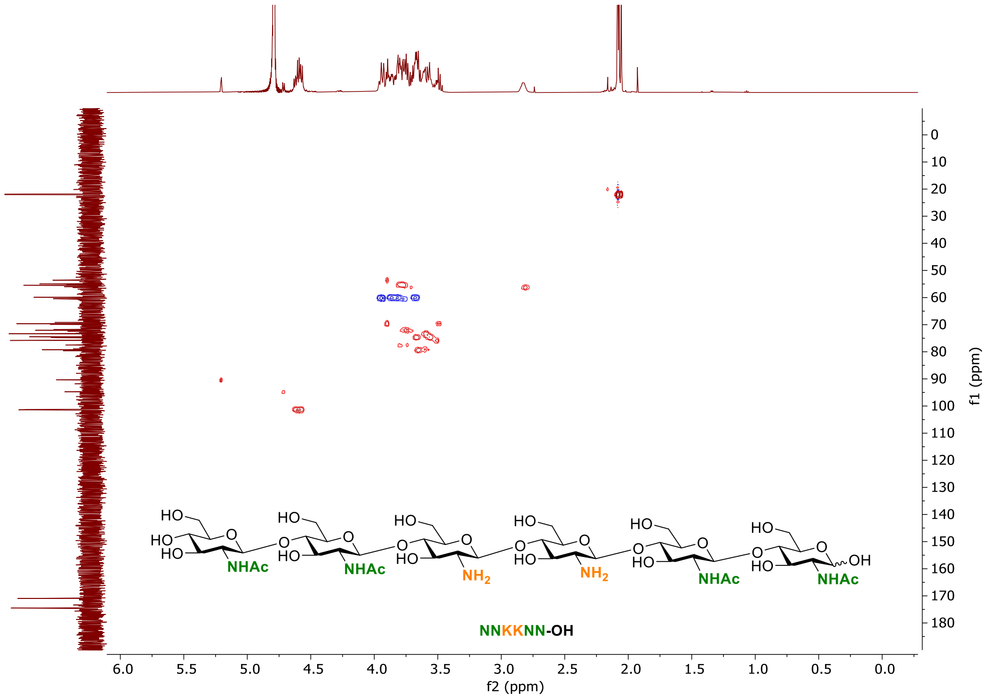

4. Synthesis and NMR analysis of dimer 5 (KN-NH_2)

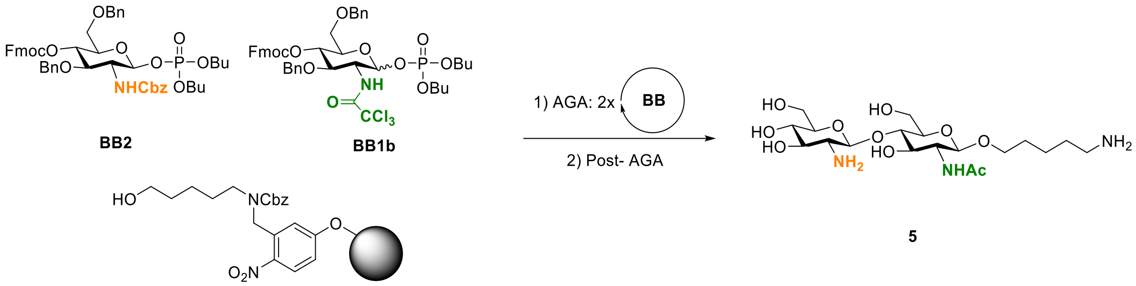

Step		Modules	Notes
AGA		**A**	
	**BB1b**	**B**, **C***, **D**, **E**	**C***:(**BB1b**, -35° for 5 min, -10° for 40 min)
	**BB2**	**B**, **C***, **D**, **E**	**C***:(**BB2**, -35° for 5 min, -10° for 40 min)
Post-AGA		**G**, **I1**, **H**, **I2**	**I1**: (Method B, t_R = 24.9 min),<br>**I2**: (Method D, t_R =18.2 min)

Automated synthesis, global deprotection, and purification afforded KN-NH_2 as white solid (0.9 mg, 15% overall yield).

Analytical data for **KN-NH_2**: ^{1}H NMR (600 MHz, D_2O) δ 4.56 – 4.52 (m, 1H), 4.50 (d, *J* = 8.0 Hz, 1H), 3.95 (dd, *J* = 22.7, 12.2 Hz, 3H), 3.88 – 3.80 (m, 1H), 3.75 (dd, *J* = 19.1, 7.9 Hz, 4H), 3.67 – 3.58 (m, 2H), 3.51 (t, *J* = 7.6 Hz, 1H), 3.41 (m, 2H), 3.06 – 2.97 (m, 2H), 2.69 (t, *J* = 8.6 Hz, 1H), 2.05 (s, 3H), 1.69 (m, 2H), 1.62 (m, 2H), 1.46 – 1.37 (m, 2H). ^{13}C NMR (151 MHz, D_2O) δ 174.1, 102.7, 100.99, 78.40, 76.11, 74.56, 72.24, 70.04, 69.47, 60.54, 60.17, 56.61, 55.17, 39.27, 28.01, 26.35, 22.08, 22.05. m/z (HRMS+) 468.2537 $[M + H]^+$ ($C_{19}H_{38}N_3O_{10}$ requires 468.2557).

RP-HPLC of KN-NH2 (ELSD trace, Method D, t_R = 18.2 min)

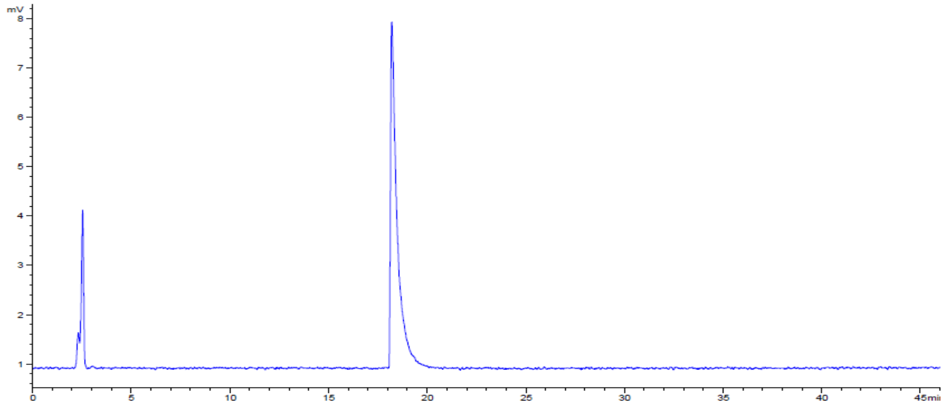

^{1}H NMR of KN-NH_2 (600 MHz, D_2O)

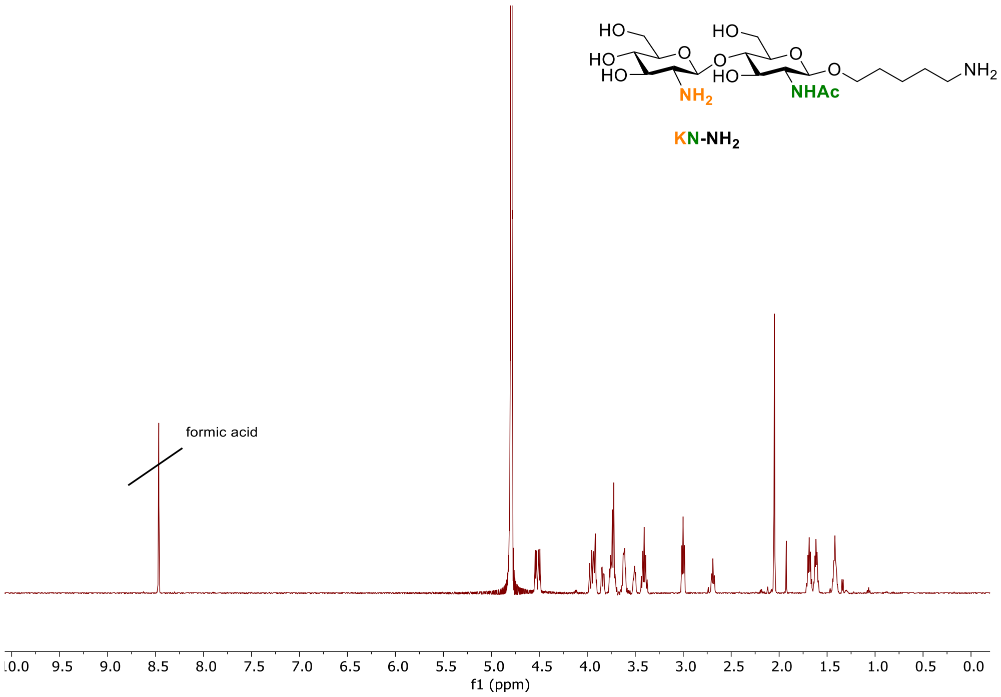

^{13}C NMR of KN-NH_2 (151 MHz, D_2O)

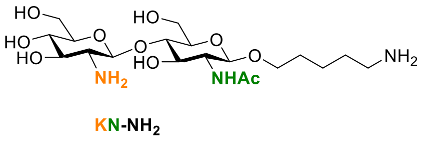

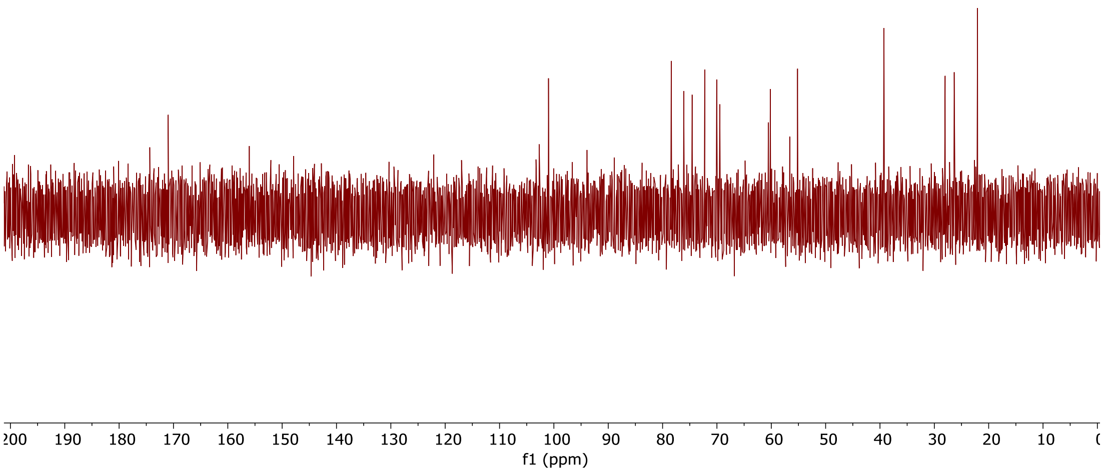

HSQC NMR of KN-NH_2 (D_2O)

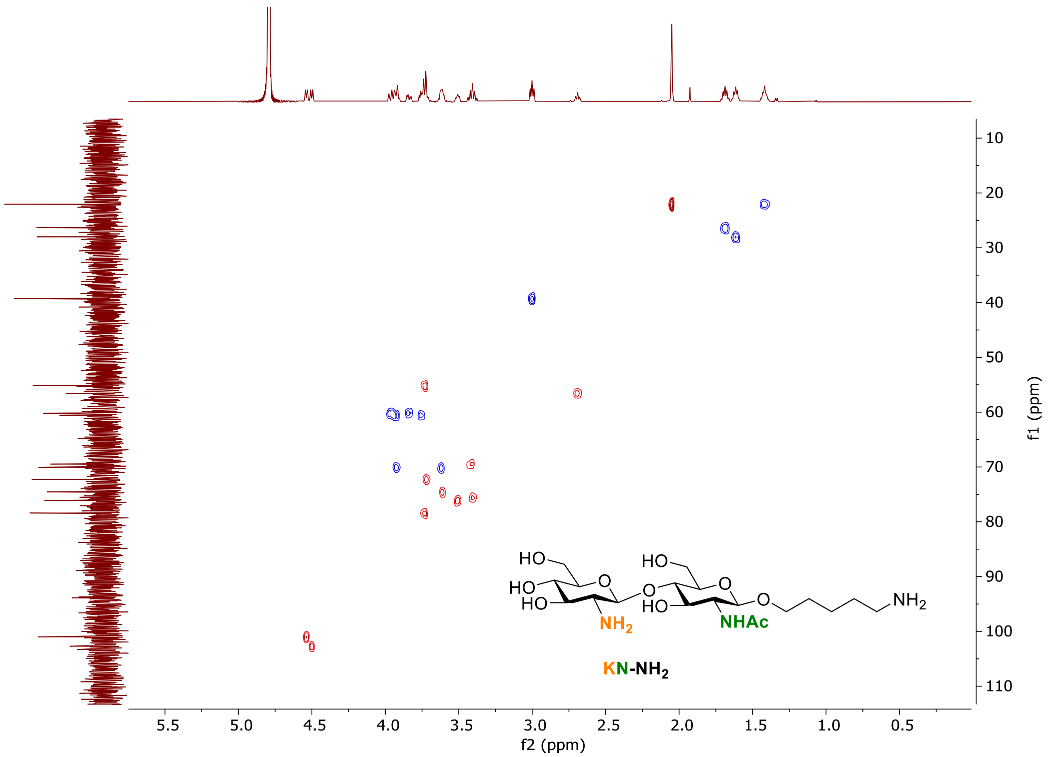

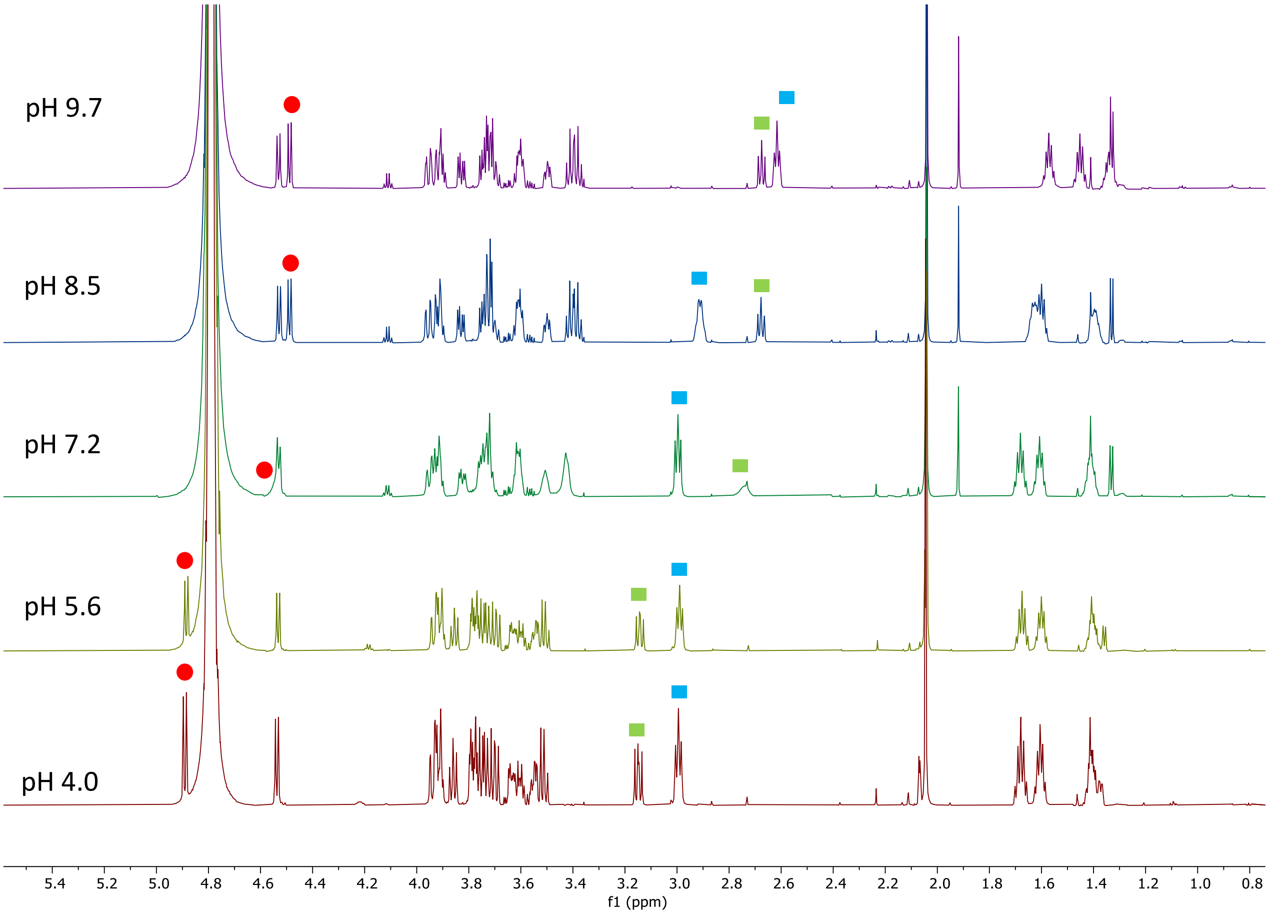

Figure S1: ^{1}H-NMR titration of **KN**-NH_2. pH was adjusted using solutions of HCl (0.1% in D_2O) and NaOH (0.1% in D_2O). The shift of selected signals during the titration is highlighted with: ● H1 (GlcN), ■ H2 (GlcN), ■ CH_2-N (Linker).

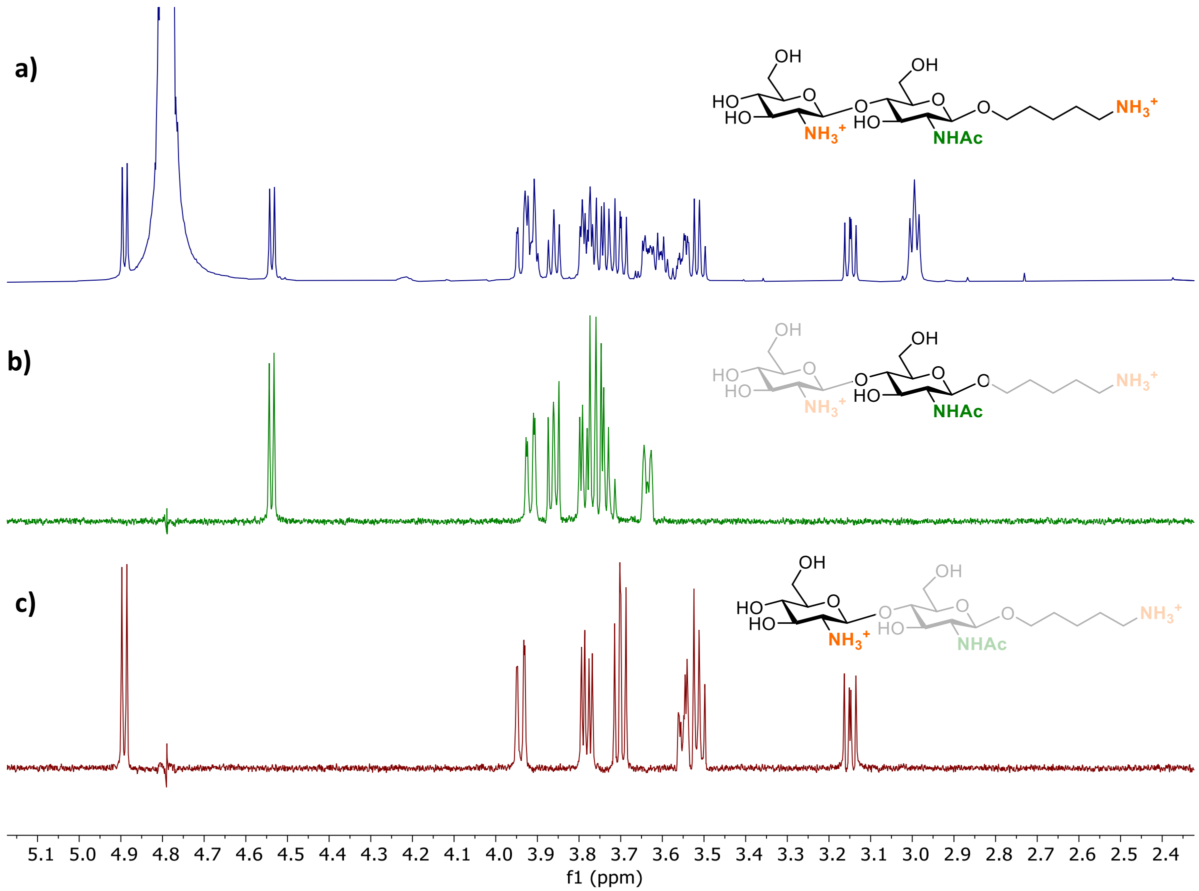

Figure S2: a) Representative ^{1}H-NMR spectrum of **KN**-NH_2 recorded at pH 4.0. b) Selective 1D HOHAHA-NMR spectrum with selective excitation of the GlcNAc (N) anomeric proton at 4.5 ppm. c) Selective 1D HOHAHA-NMR spectrum with selective excitation of GlcN (K) anomeric proton at 4.9 ppm.

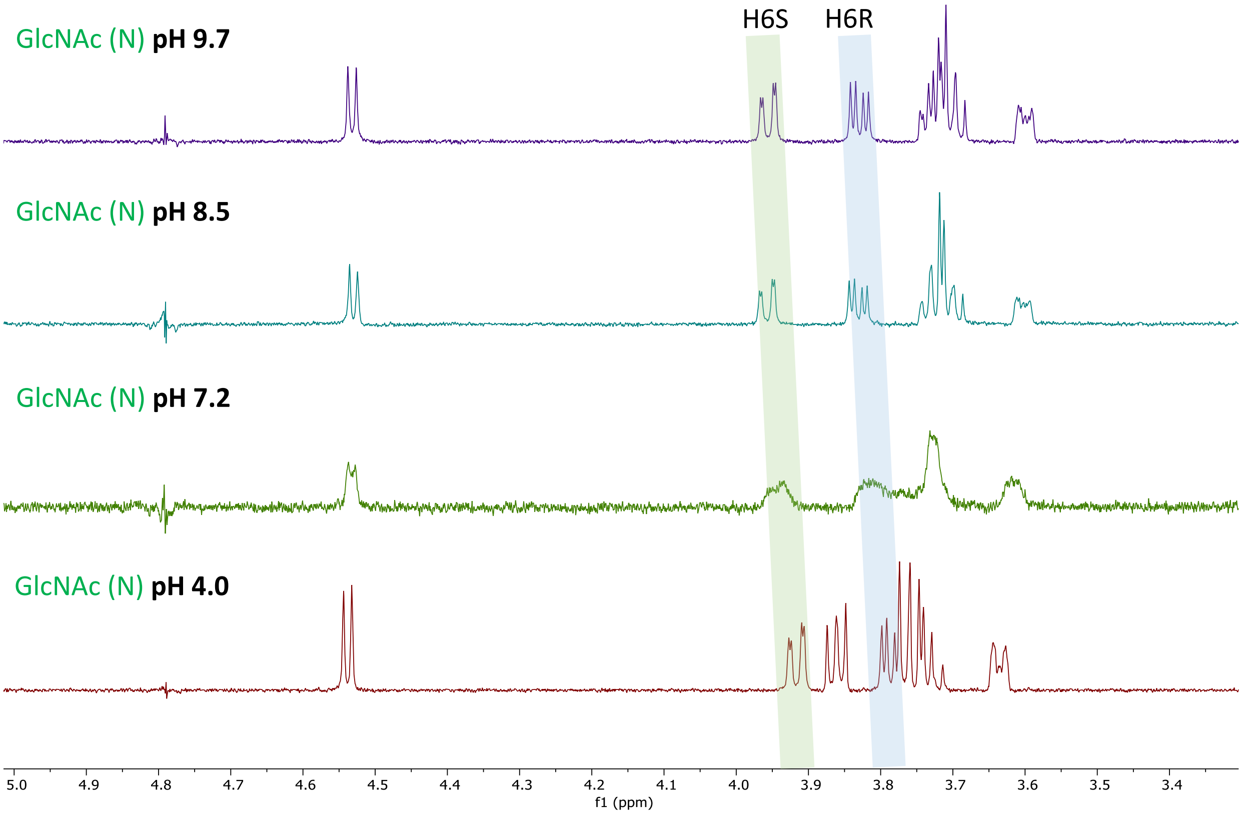

Figure S3: Selective 1D HOHAHA-NMR spectra of **KN**-NH_2 with selective excitation of the GlcNAc anomeric proton at 4.5 ppm obtained at different pH.

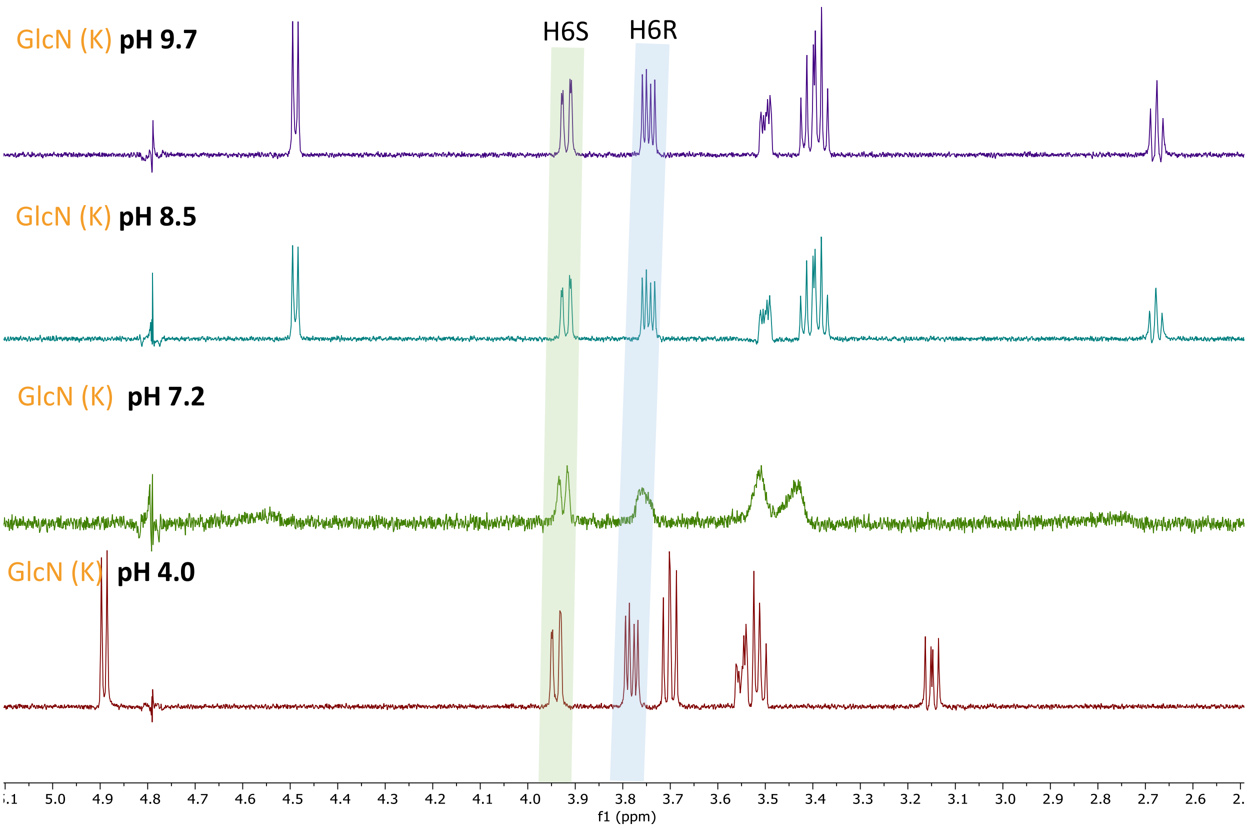

Figure S4: Selective 1D HOHAHA-NMR spectra of **KN**-NH_2 with selective excitation of the GlcN anomeric proton at 4.9-4.5 ppm obtained at different pH.

Table S1: Experimentally calculated coupling constants $^3J_{H5H6R}$ and $^3J_{H5H6S}$ at different protonation stages and rotamers population (f) calculated using equations [1-3].[3]

GlcNAc (N)	pH 4.0	pH 7.2	pH 8.5	pH 9.7
$^3J_{H5H6R}$	4.9 Hz	-	5.1 Hz	5.1 Hz
$^3J_{H5H6S}$	2.6 Hz	-	2.3 Hz	2.4 Hz
f_{gt}	38 %	-	41 %	41 %
f_{gg}	59 %	-	59 %	58 %
f_{tg}	**4 %**	-	0 %	1 %
**GlcN (K)**				
$^3J_{H5H6R}$	5.6 Hz	-	6.0 Hz	6.1 Hz
$^3J_{H5H6S}$	2.1 Hz	-	2.3 Hz	2.3 Hz
f_{gt}	47 %	-	50 %	51 %
f_{gg}	56 %	-	50 %	49 %
f_{tg}	-3 %	-	-1 %	-1 %

$$^3J_{H5H6R} = {}^3J_{R,gg}f_{gg} + {}^3J_{R,gt}f_{gt} + {}^3J_{R,tg}f_{tg} \qquad [1]$$

$$^3J_{H5H6s} = {}^3J_{S,gg}f_{gg} + {}^3J_{S,gt}f_{gt} + {}^3J_{S,tg}f_{tg} \qquad [2]$$

$$1 = f_{gg} + f_{gt} + f_{tg} \qquad [3]$$

Several limiting coupling constants ($^3J_{R,gg}$, $^3J_{R,gt}$, $^3J_{R,tg}$ and $^3J_{S,gg}$, $^3J_{S,gt}$, $^3J_{S,tg}$) are available in the literature. We report the results obtained with the values reported in *Table S2*.

Table S2: Limiting coupling constants reported in Ref [3].

	$^3J_{H5H6R}$			$^3J_{H5H6S}$		
	gg	*gt*	*tg*	*gg*	*gt*	*tg*
**Ref. [3]**	1.0	11.0	4.8	2.2	2.5	10.2

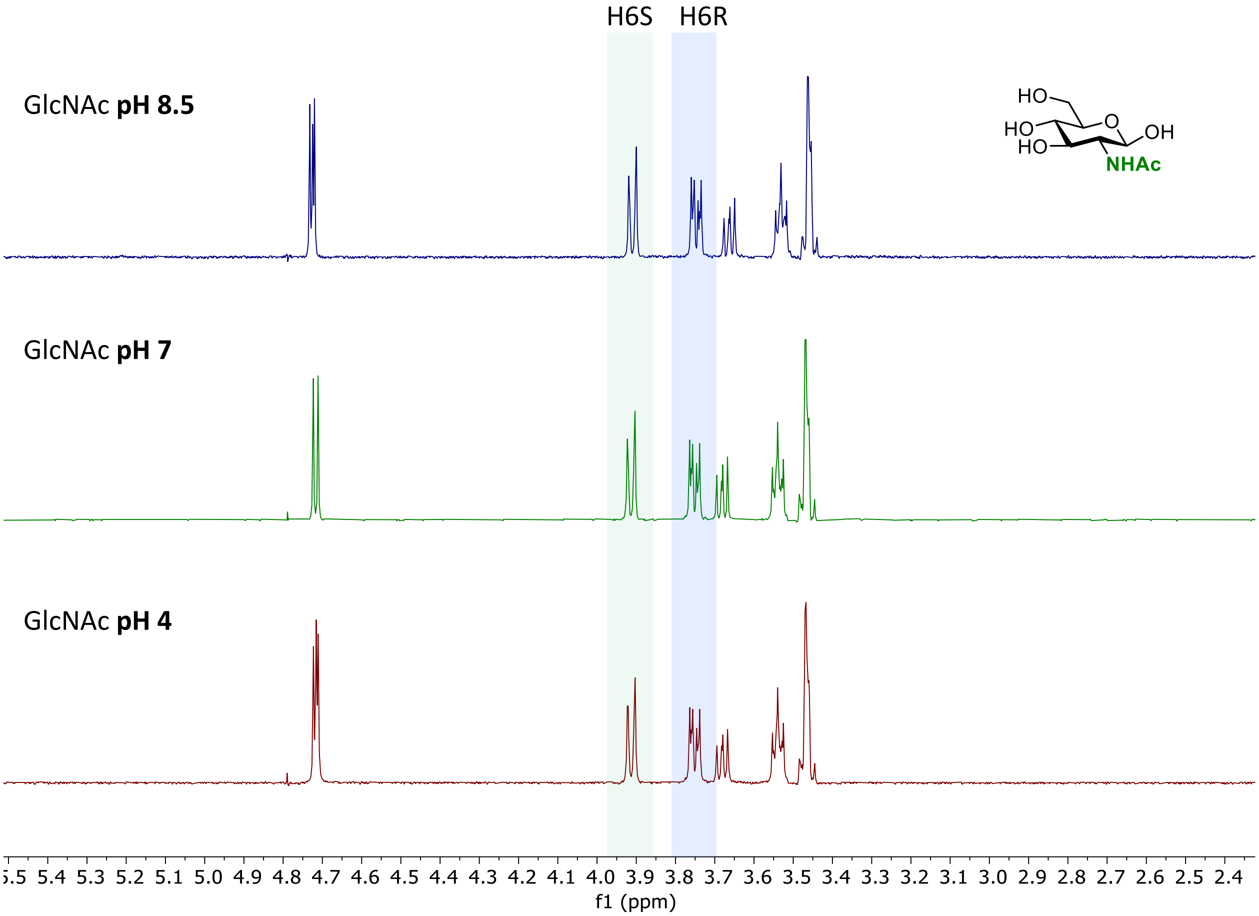

Figure S5: Control experiment showing that $^{3}J_{H5H6R}$ and $^{3}J_{H5H6S}$ measured for the monosaccharide GlcNAc are not affected by pH. Selective 1D HOHAHA-NMR spectra of β-GlcNAc with selective excitation of the anomeric proton at 4.7 ppm obtained at different pH.

Table S3: Experimentally calculated coupling constants $^{3}J_{H5H6R}$ and $^{3}J_{H5H6S}$ at different pH, confirming that the values are not significantly affected by the pH of the solution.

**β-GlcNAc**	**pH 4.0**	**pH 7.0**	**pH 8.5**
$^{3}J_{H5H6R}$	5.3 Hz	5.3 Hz	5.2 Hz
$^{3}J_{H5H6S}$	1.8 Hz	1.7 Hz	1.7 Hz

5. XRD Analysis

X-ray diffraction experiments were carried out using a D8 Avance diffractometer (Bruker) in reflection mode with monochromatic Cu Kα radiation (λ= 1.5418 Å) generated at 40 kV and 40 mA (Siemens X-ray tube KFL CU 2K). The scans were performed in the scattering angle range between 4°and 40°with a step of 0.02°and an accumulation time of 6 s. The oligosaccharide samples were lyophilized prior to XRD measurement. Raw XRD profiles were corrected by subtraction of the sample holder signal, smoothing and baseline correction.

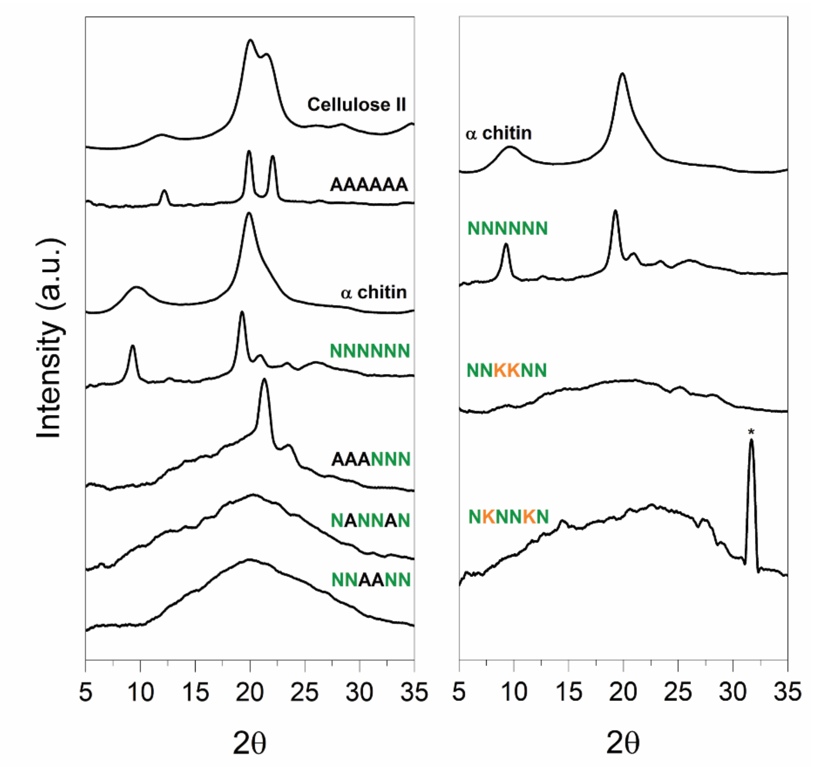

Figure S6: Powder XRD profiles of synthetic COS and hybrid chitin-cellulose oligomers. The XRD profiles obtained for the polysaccharide α-chitin and cellulose II are reported for comparison. XRD profiles for A_6 and N_6 were previously reported.[4] *Crystalline carbonate peak resulting from the prolonged exposure of the polyamino compound to air.[5]

6. Molecular Dynamics Simulations

All-atom molecular dynamics (MD) simulations were performed using gromacs 5.1.2[6]. The oligosaccharides were modeled using the modified $GLYCAM06_{OSMO,r14}$ force field[7], and the system was solvated with TIP5P[8] water molecules to avoid excessive interactions between the monomers.

Initial conformations of single hexamers were constructed with tleap. The topology was converted to gromacs format using the glycam2gmx.pl script and solvated with 2100 water molecules using gromacs tools. The systems were kept at a constant temperature of 300 K using a Nosé-Hoover thermostat[9] and at constant pressure of 1 bar with the Parrinello-Rahman barostat[10]. Non-bonded interactions were cut-off at 1.4 nm, long range electrostatics were calculated using the particle mesh Ewald method[11]. Bonds involving hydrogens were constrained using the LINCS[12] to allow a 2 fs time step algorithm; water molecules were kept rigid with SETTLE[13].

After energy minimization (steepest descent algorithm) and before the production run, the systems were equilibrated at 300 K for 50 ns in a canonical (NVT) ensemble (constant number of particles, volume and temperature) and subsequently at 300 K and 1 bar for 50 ns in an isothermal-isobaric (NPT) ensemble. All hexamers were simulated for 500 ns and concentrated solutions for 1 µs.

In each oligosaccharide the residues are numbered from the non-reducing end (R1) to the reducing end (R6).

Definition of dihedrals exemplified for a β-1,4-glucose disaccharide

ω = O5C5C6O6

Ψ = C1O4C4H4

Φ = H1C1O4C4

(The atoms in red belong to the following residue)

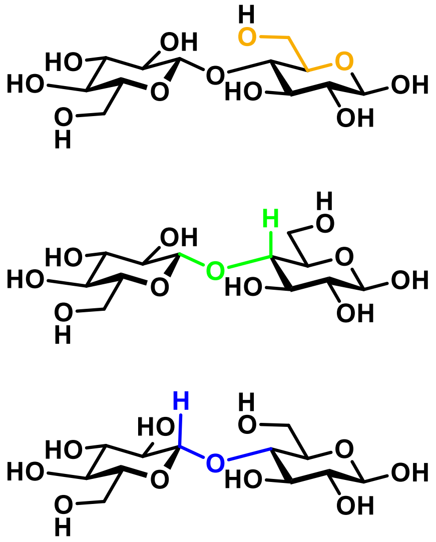

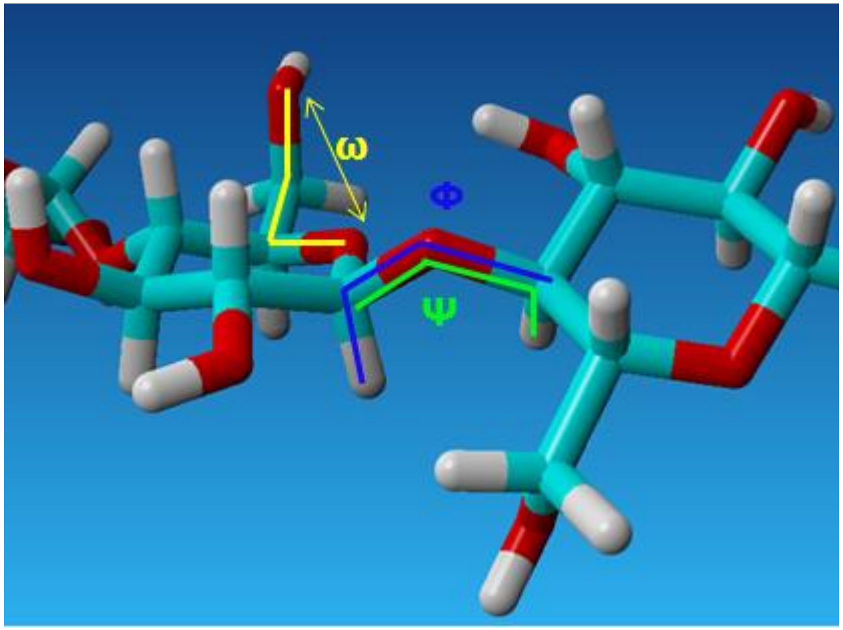

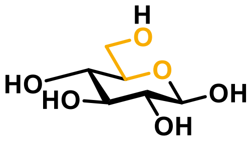

gt (ω = 60°)

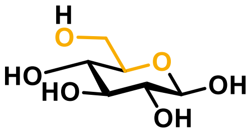

tg (ω = 180°)

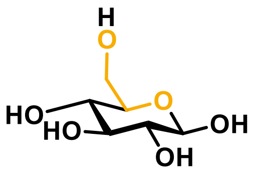

gg (ω = - 60°)

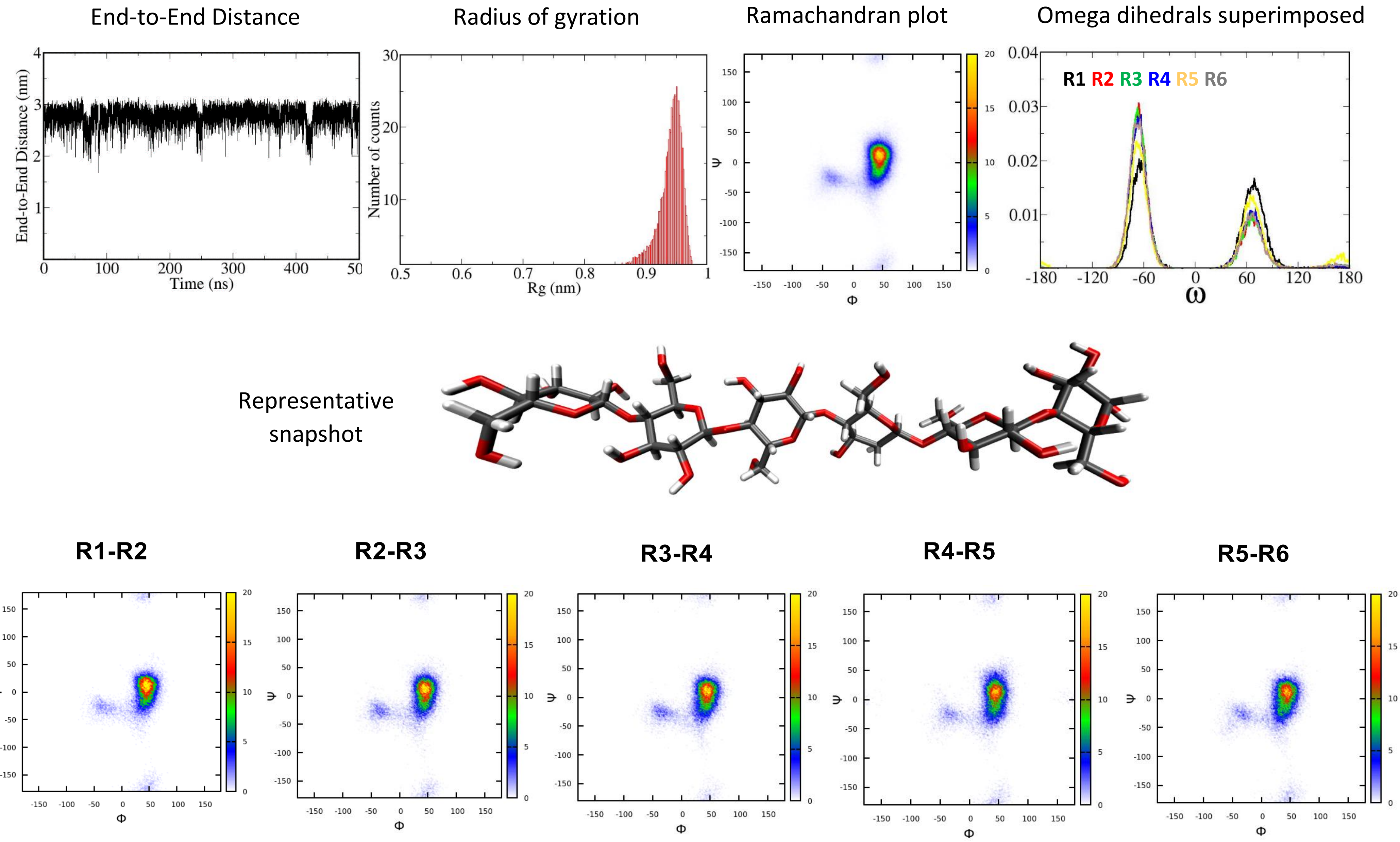

Figure S7: MD characterization of the hexaglucoside $\mathbf{A_6}$ (for comparison).[4]

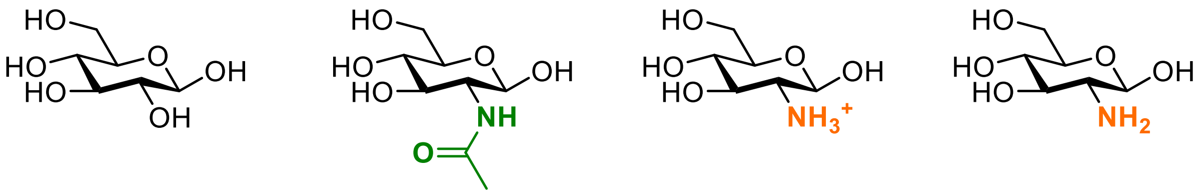

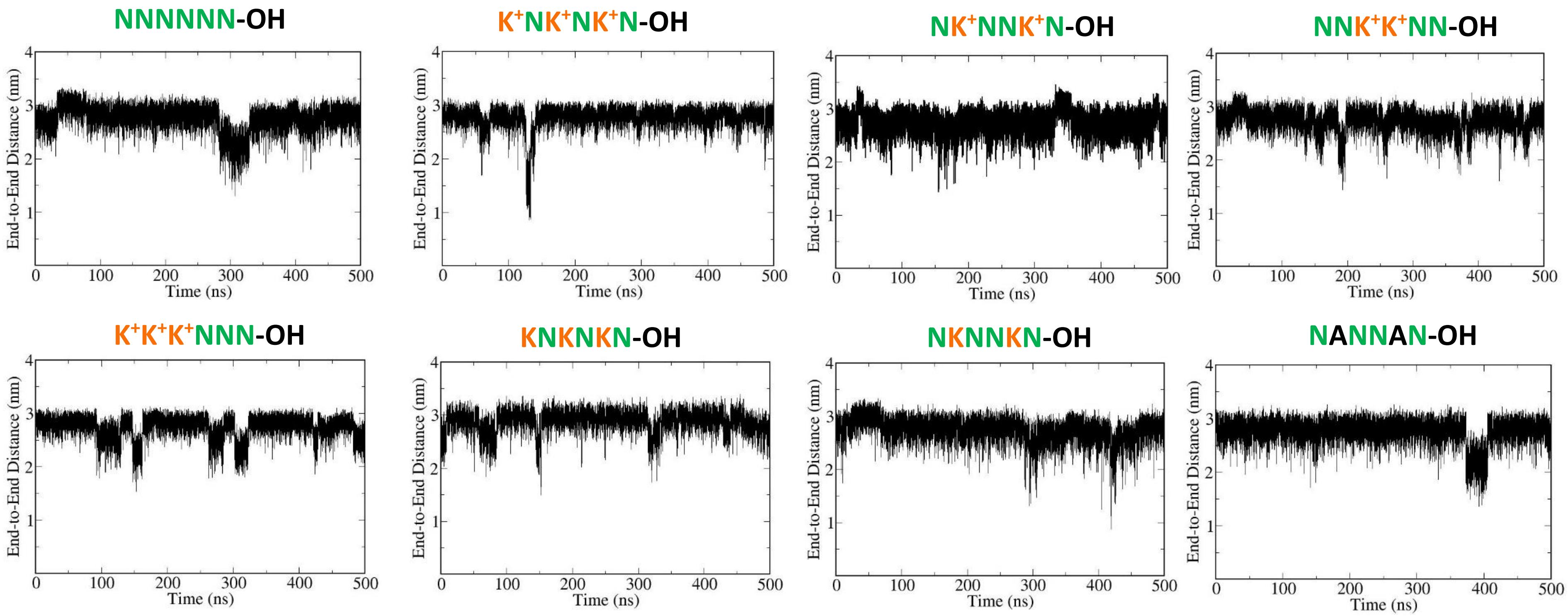

Hexamer	NNNNNN-OH	$K^{+}NK^{+}NK^{+}N$-OH	KNKNKN-OH	$NNK^{+}K^{+}NN$-OH	NNKKNN-OH	$K^{+}K^{+}K^{+}NNN$-OH	$NK^{+}NNK^{+}N$-OH	NKNNKN-OH	NANNAN-OH
Average Distance (nm)	2.76	2.76	2.88	2.73	2.73	2.73	2.73	2.73	2.75
Standard Deviation	0.22	0.22	0.20	0.21	0.21	0.21	0.21	0.23	0.23

Figure S8: End-to-end distance as a function of time.

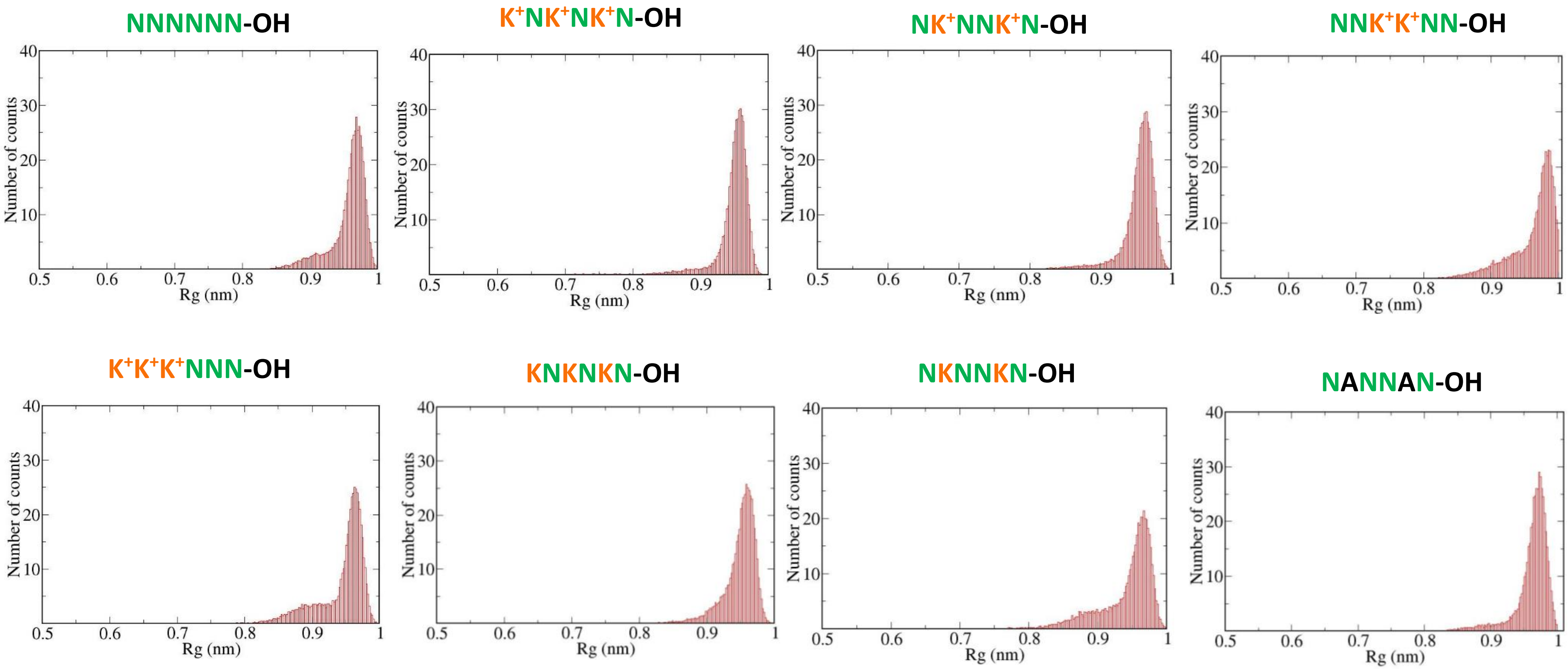

Figure S9: Histograms of radius of gyration (RoG).

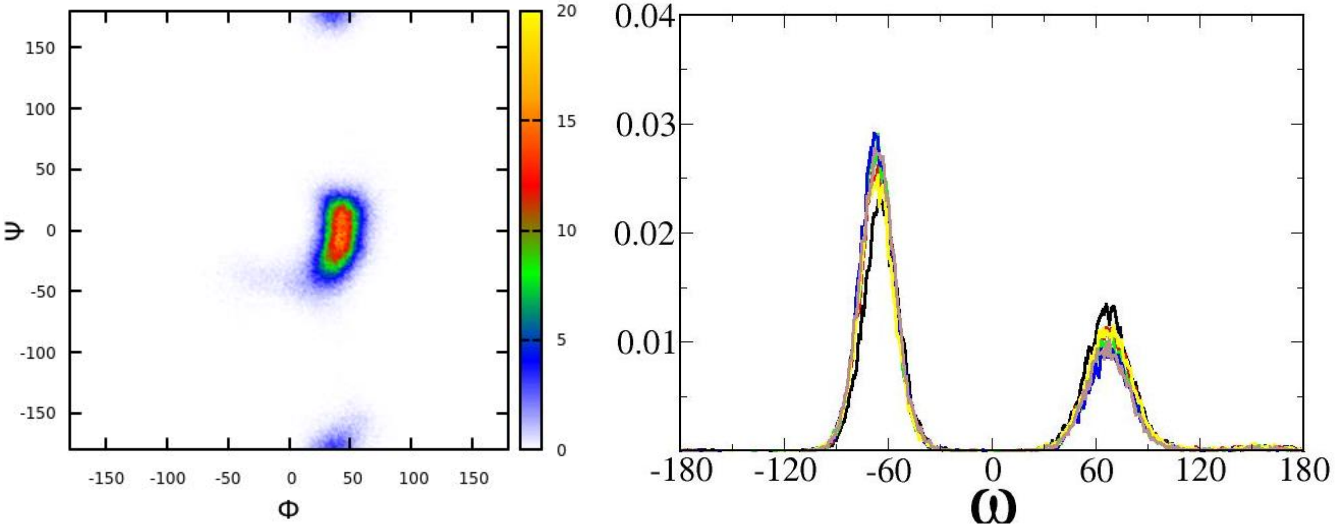

	gg(%)	gt(%)	tg(%)
**ω1**	56	43	1
**ω2**	61	38	1
**ω3**	68	31	1
**ω4**	63	36	1
**ω5**	73	26	1
**ω6**	70	29	1

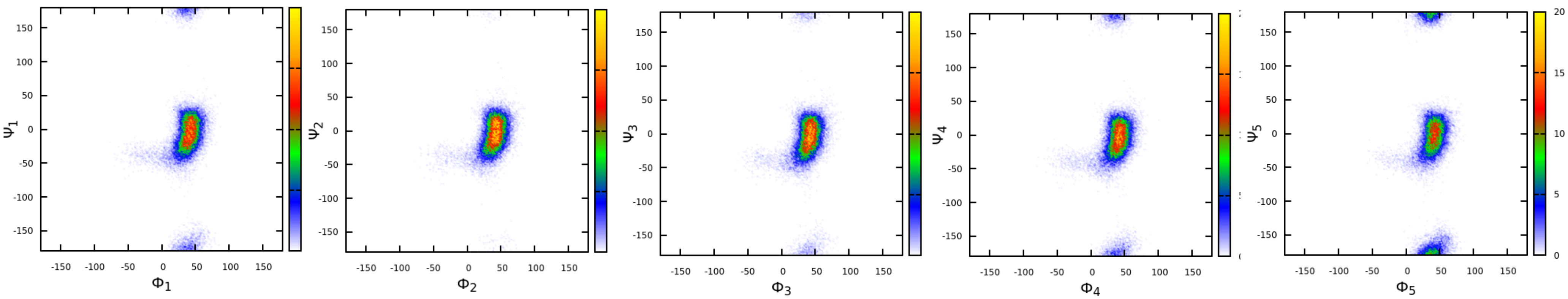

Figure S10a: Dihedral analysis of **NNNNNN-OH.**

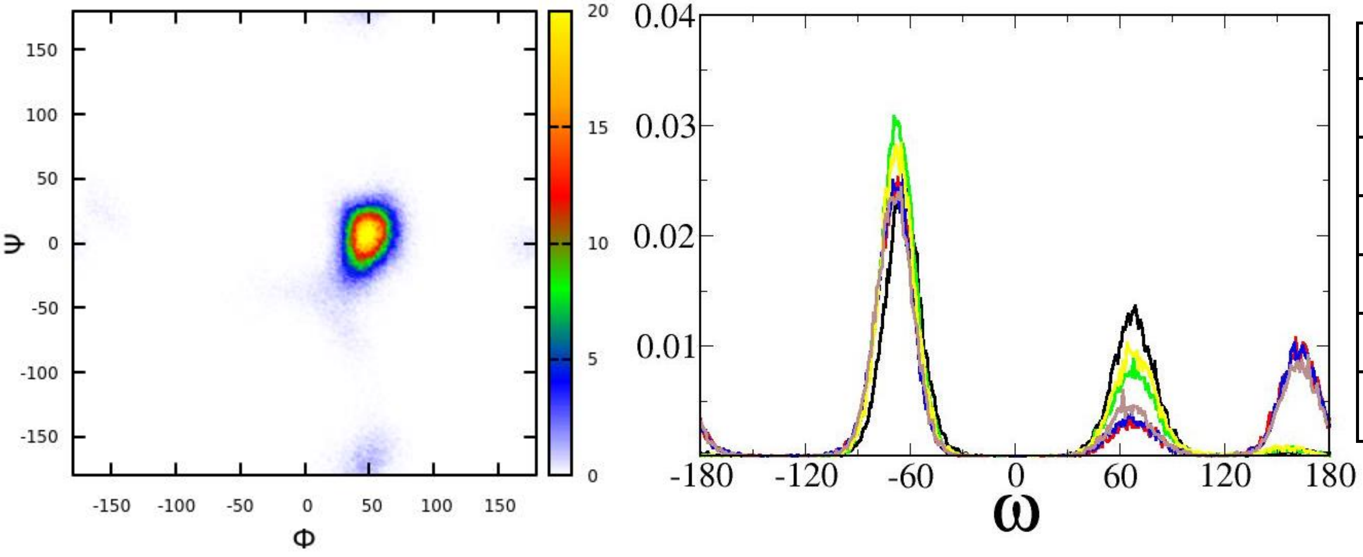

	gg(%)	gt(%)	tg(%)
**ω1**	60	38	2
**ω2**	62	10	**28**
**ω3**	73	25	2
**ω4**	62	11	**27**
**ω5**	68	30	2
**ω6**	62	14	**24**

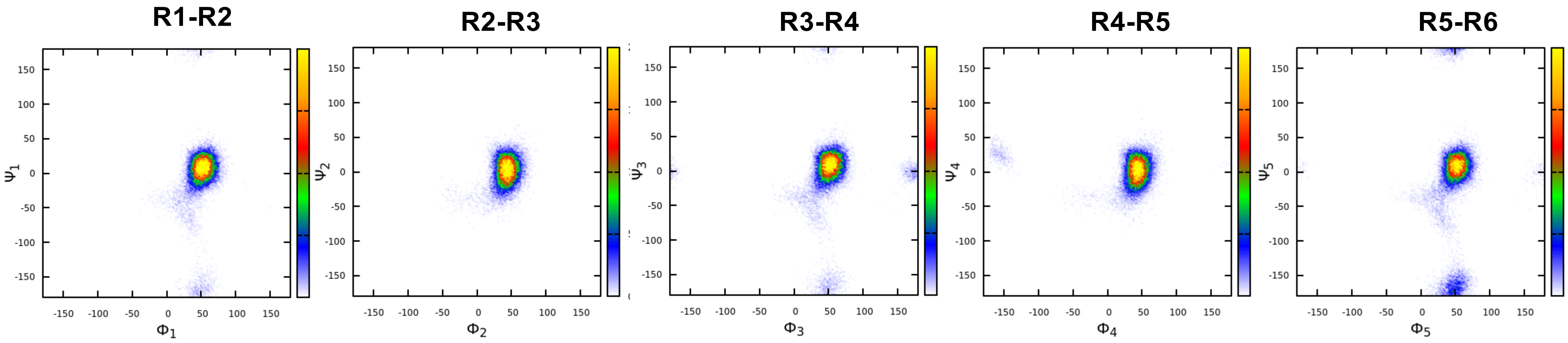

Figure S10b: Dihedral analysis of **$K^+NK^+NK^+N$-OH.**

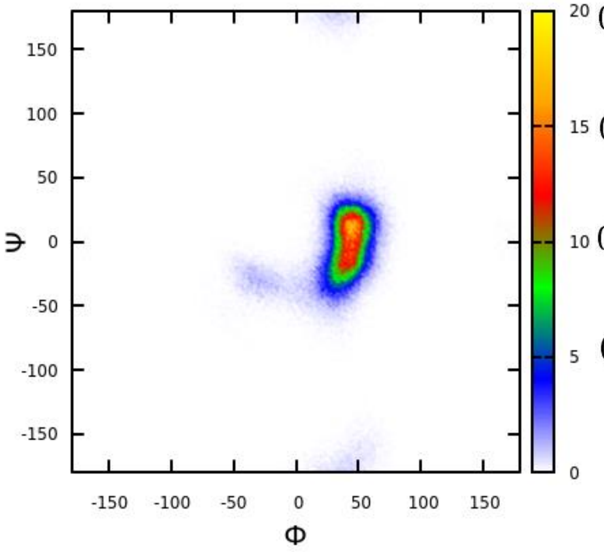

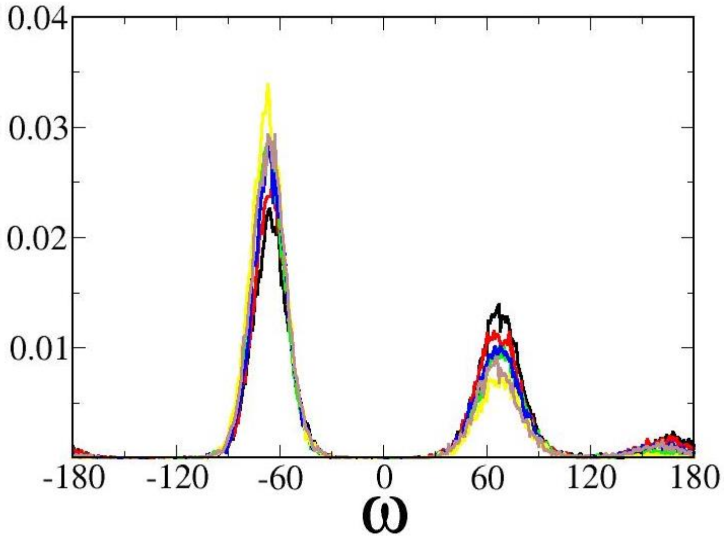

	gg(%)	gt(%)	tg(%)
**ω1**	54	40	6
**ω2**	59	36	5
**ω3**	68	30	2
**ω4**	67	31	4
**ω5**	76	22	1
**ω6**	70	25	5

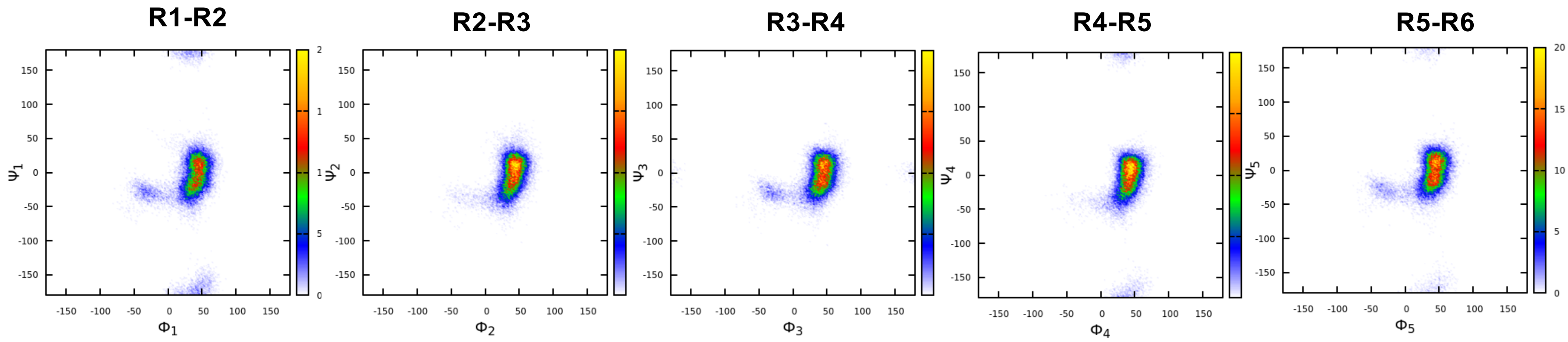

Figure S10c: Dihedral analysis of **KNKNKN-OH.**

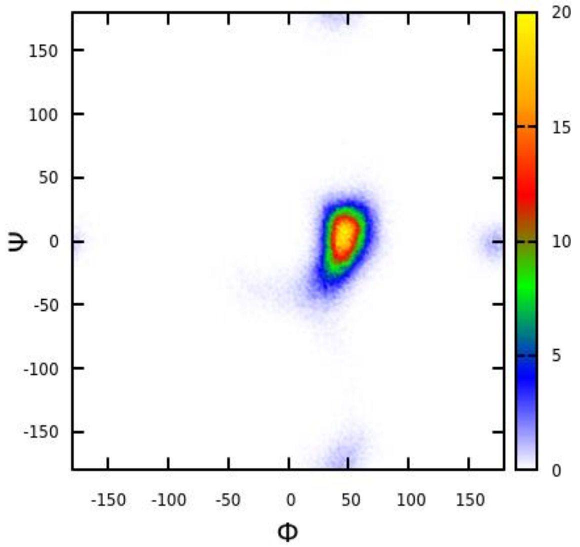

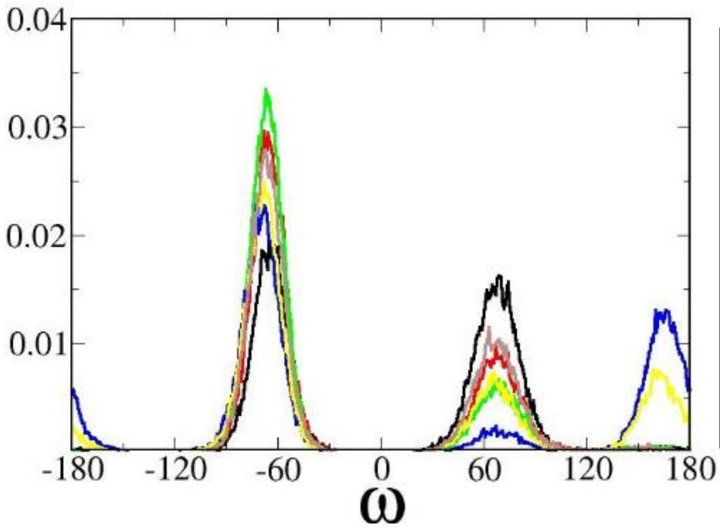

	gg(%)	gt(%)	tg(%)
**ω1**	50	49	1
**ω2**	70	29	1
**ω3**	79	19	2
**ω4**	57	6	**37**
**ω5**	60	20	**20**
**ω6**	66	33	1

R1-R2 **R2-R3** **R3-R4** **R4-R5** **R5-R6**

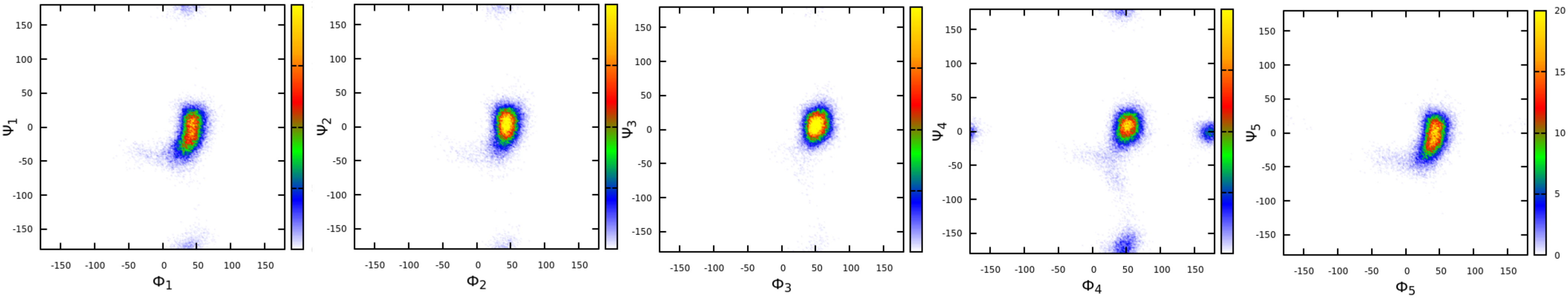

Figure S10d: Dihedral analysis of **NNKKNN-OH.**

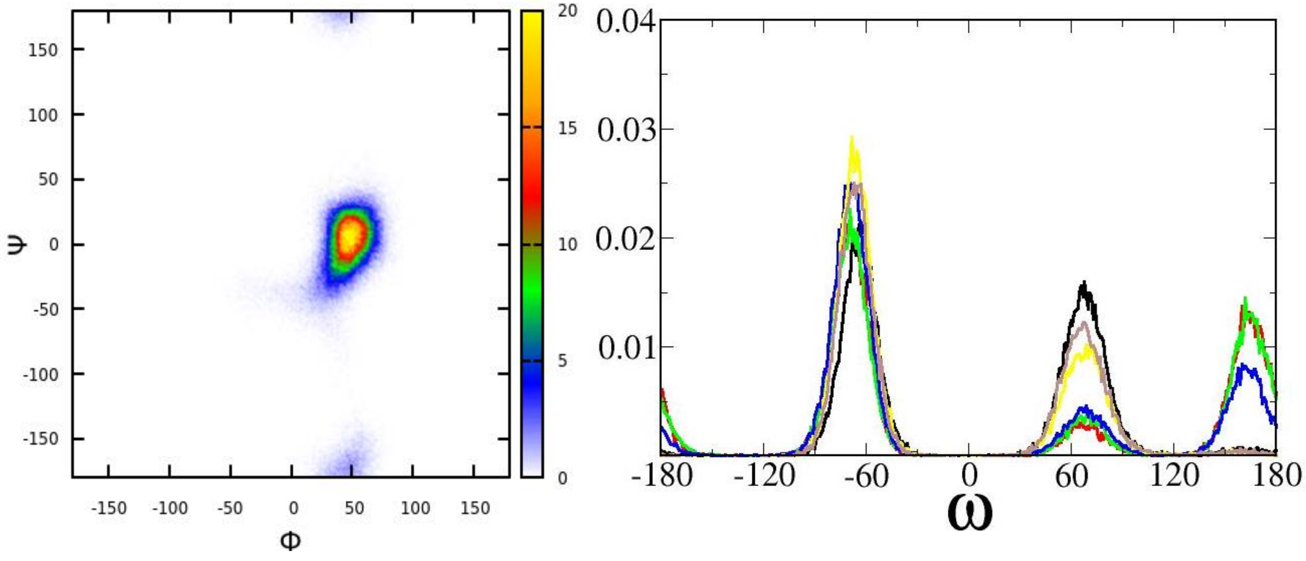

	gg(%)	gt(%)	tg(%)
**ω1**	52	46	2
**ω2**	54	9	**38**
**ω3**	53	10	**37**
**ω4**	63	13	**23**
**ω5**	67	32	1
**ω6**	61	37	2

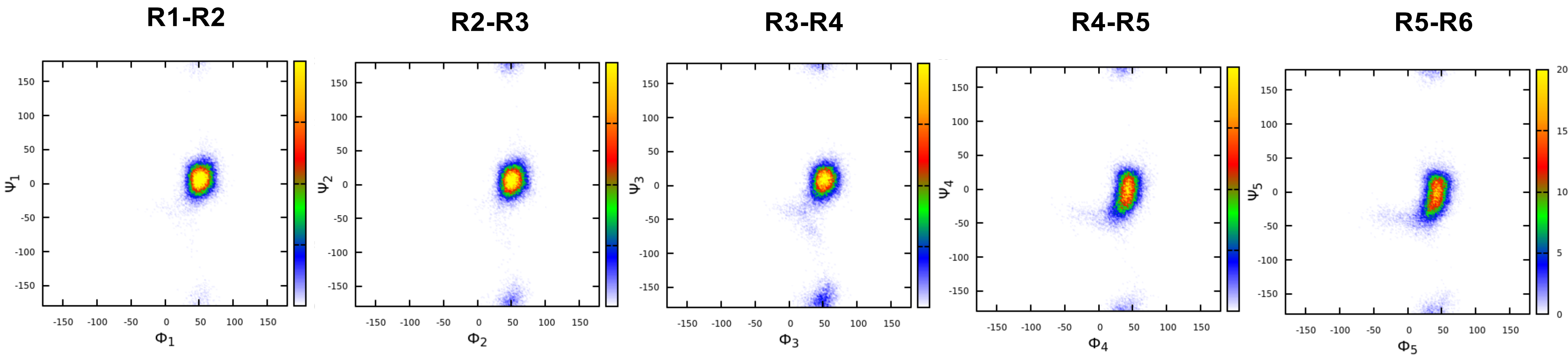

Figure S10e: Dihedral analysis of **$K^+K^+K^+$NNN-OH.**

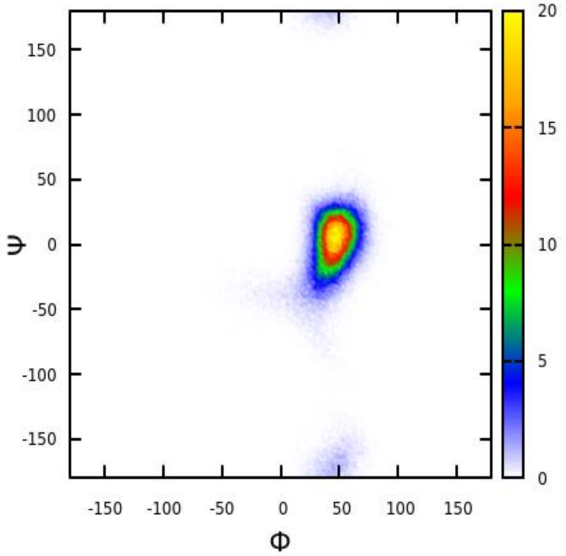

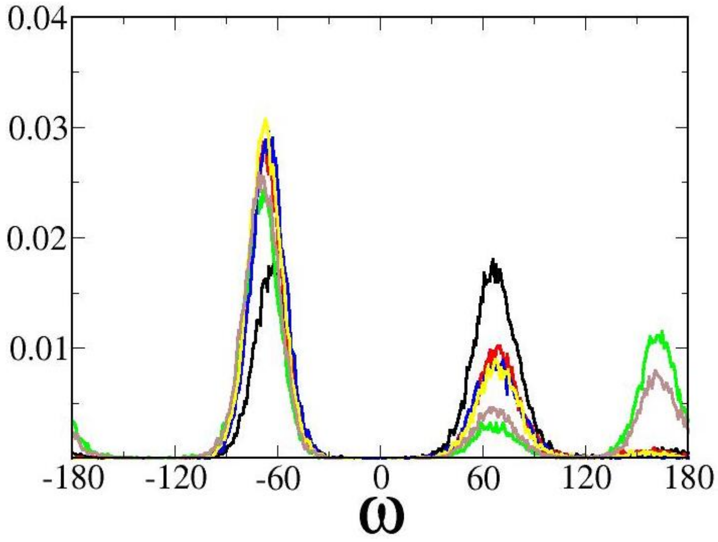

	gg(%)	gt(%)	tg(%)
**ω1**	44	54	2
**ω2**	68	30	2
**ω3**	59	9	32
**ω4**	71	28	1
**ω5**	72	26	1
**ω6**	65	13	22

R1-R2 **R2-R3** **R3-R4** **R4-R5** **R5-R6**

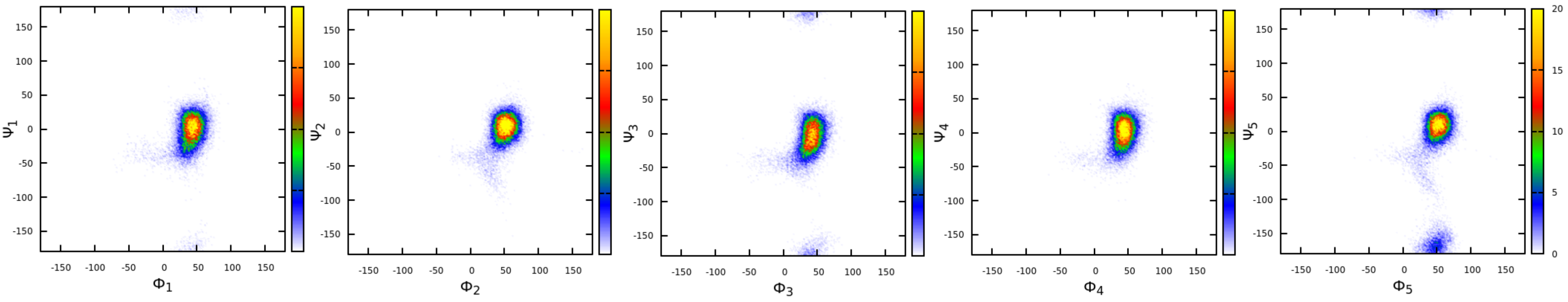

Figure S10f: Dihedral analysis of **NK⁺NNK⁺N-OH.**

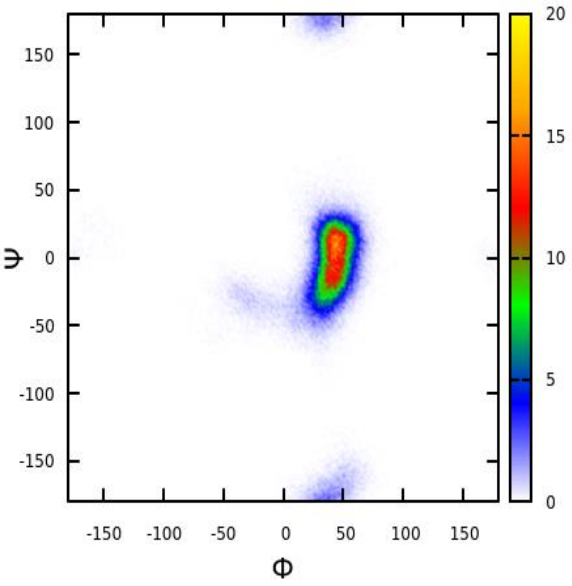

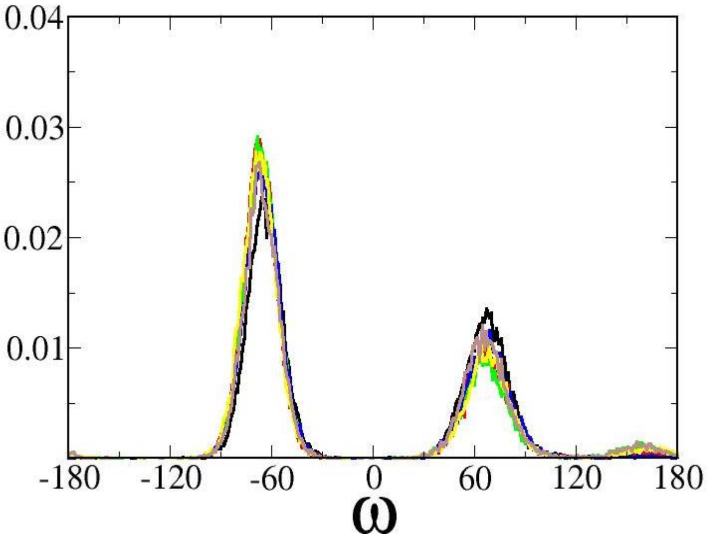

	gg(%)	gt(%)	tg(%)
**ω1**	59	40	1
**ω2**	68	30	2
**ω3**	68	28	4
**ω4**	65	34	1
**ω5**	68	29	3
**ω6**	62	34	4

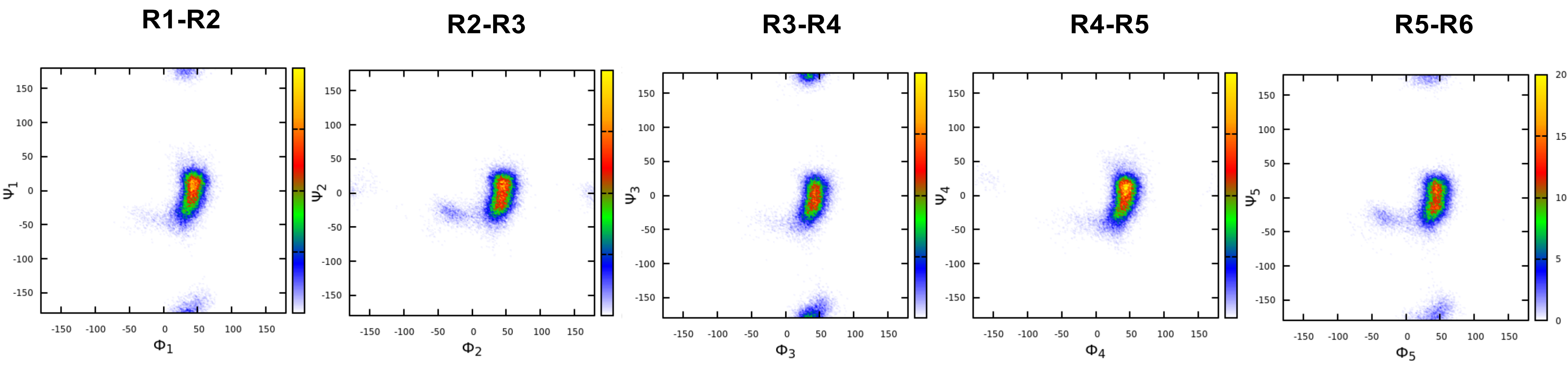

Figure S10g: Dihedral analysis of **NKNNKN-OH.**

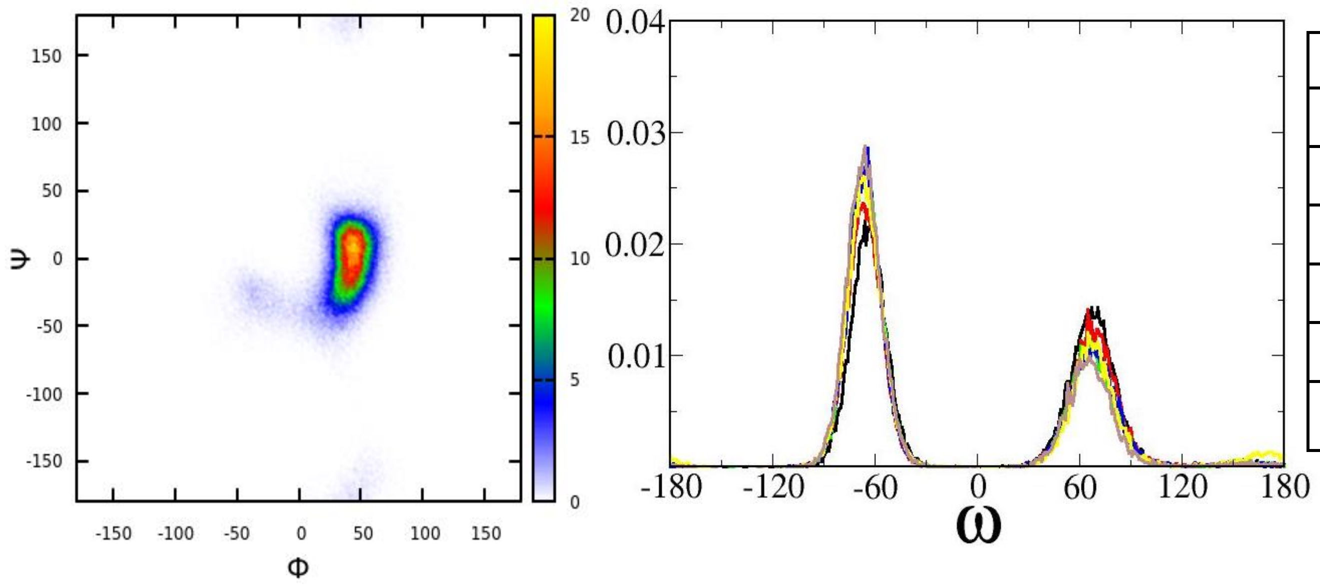

	gg(%)	gt(%)	tg(%)
**ω1**	65	34	1
**ω2**	65	32	3
**ω3**	69	30	1
**ω4**	65	34	1
**ω5**	64	35	1
**ω6**	75	24	1

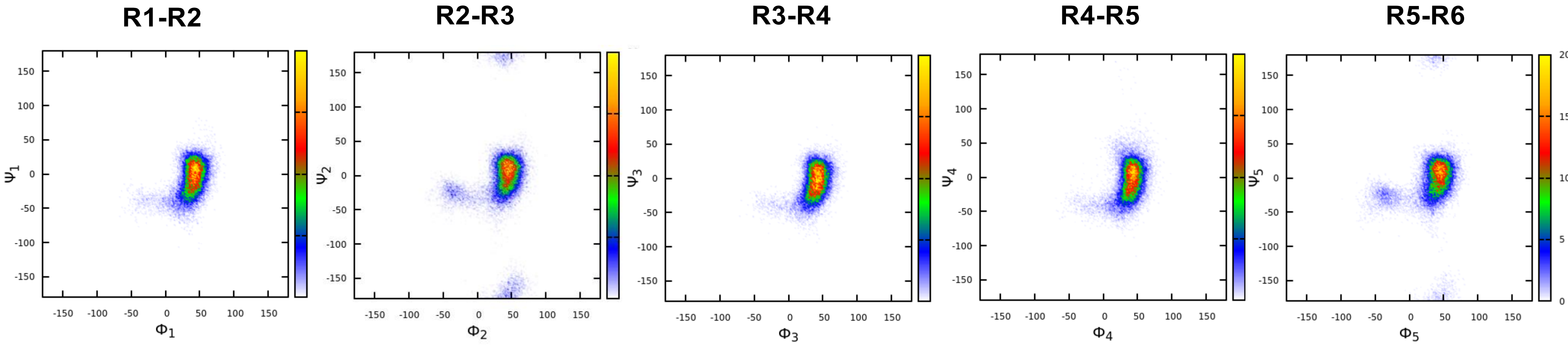

Figure S10h: Dihedral analysis of **NANNAN-OH.**

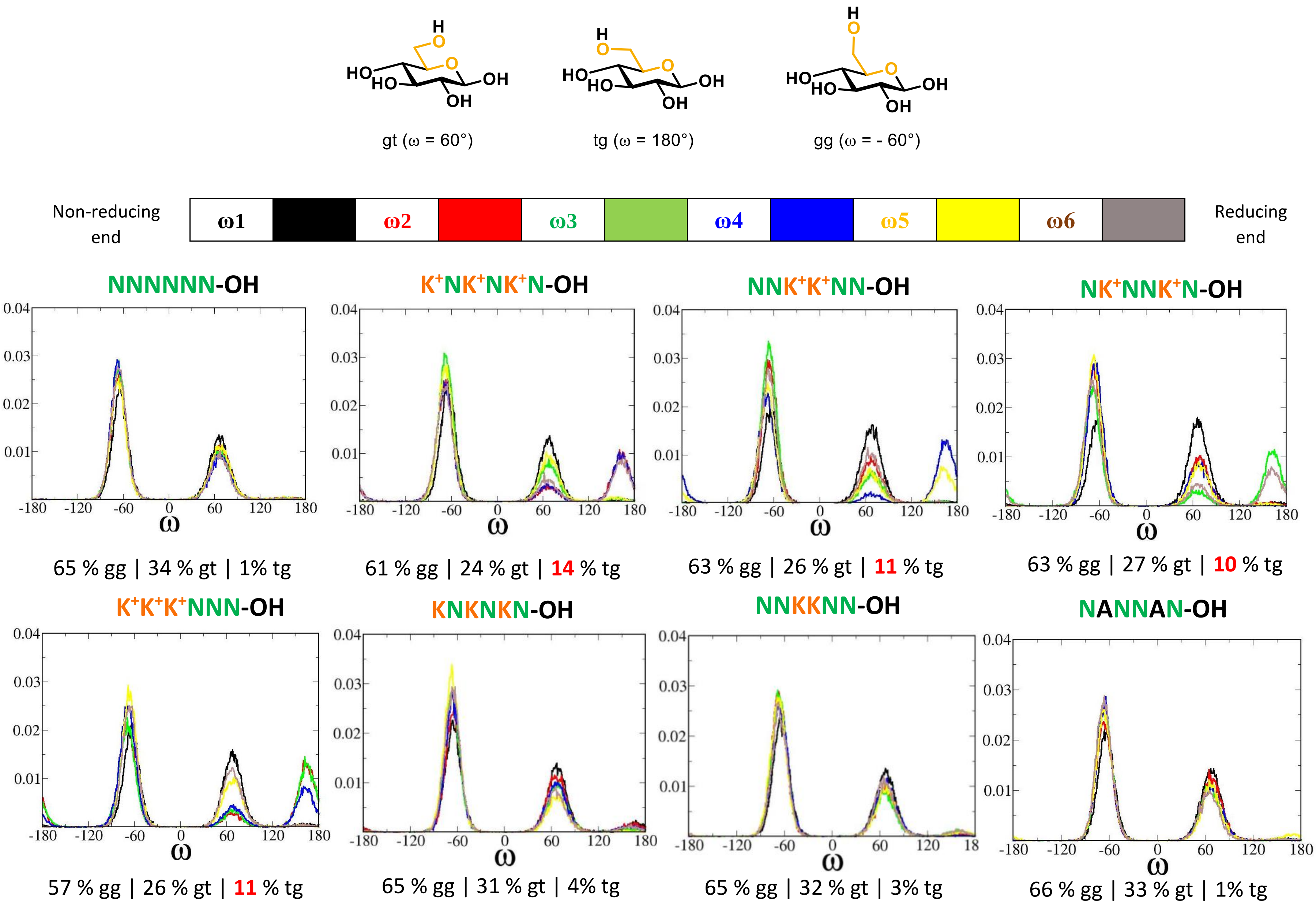

Figure S11: Superimposed omega (**ω**) torsion angles.

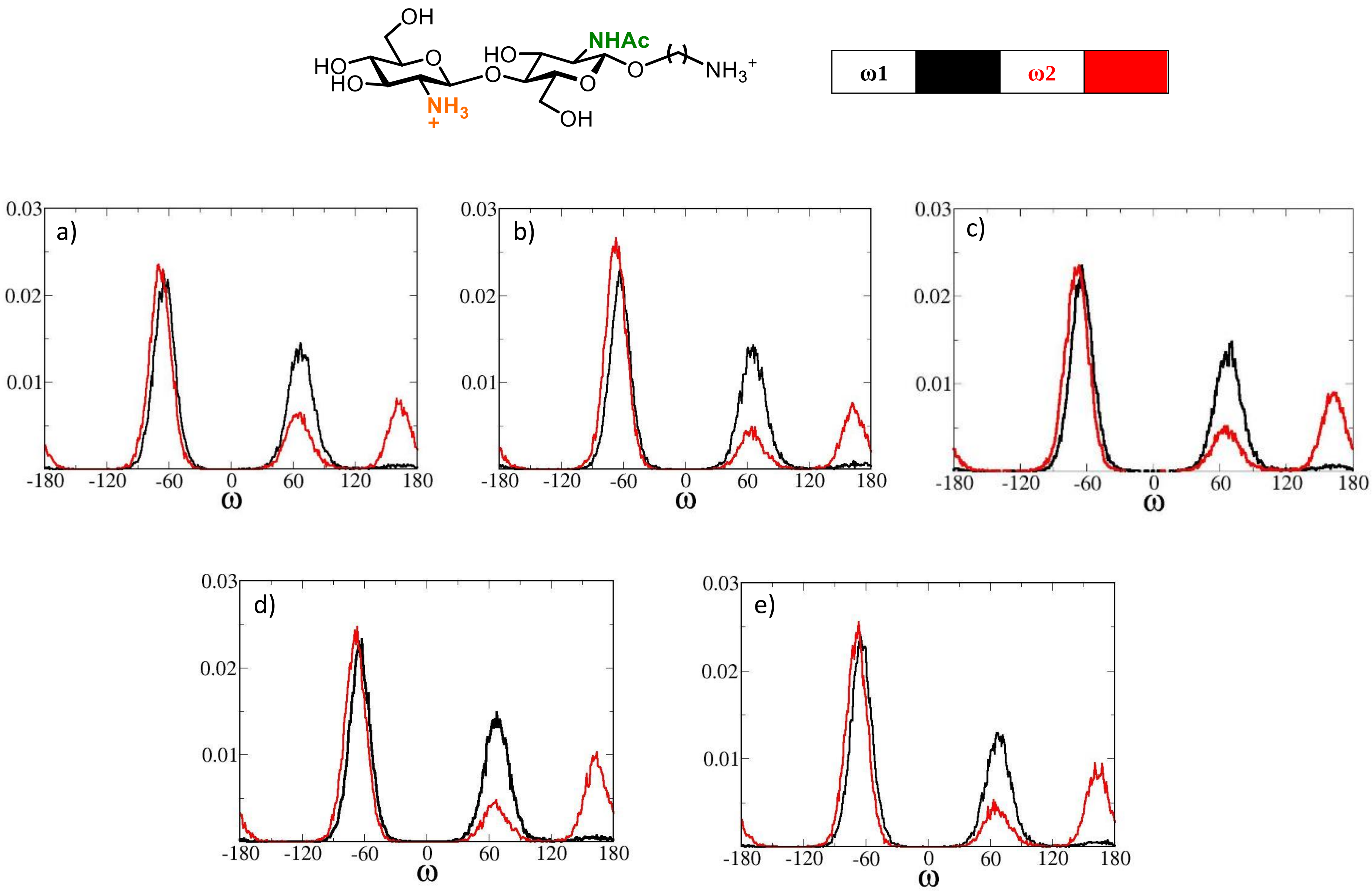

Figure S12: Omega torsional angles of K$^+$N-NH_3 measured with: (a) tip5p water model, (b) tip3p water model, (c) N3 angle parameters derived in the context of GAG, (d) increased ionic strength (50 solutes of Na^+ and Cl^-), and (d) increased ionic strength (50 solutes of Na^+ and Cl^-).

NNNNNN-OH	NANNAN-OH	NKNNKN-OH	NK⁺NNK⁺N-OH

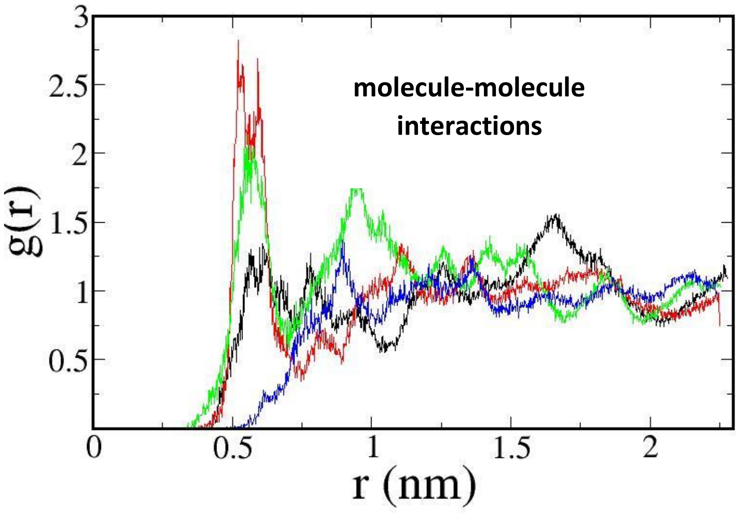

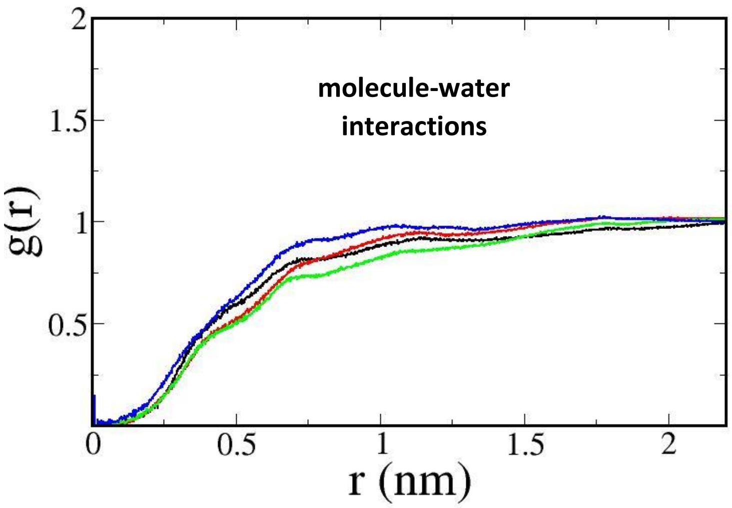

Intermolecular hydrogen bonds (concentrated exp.)

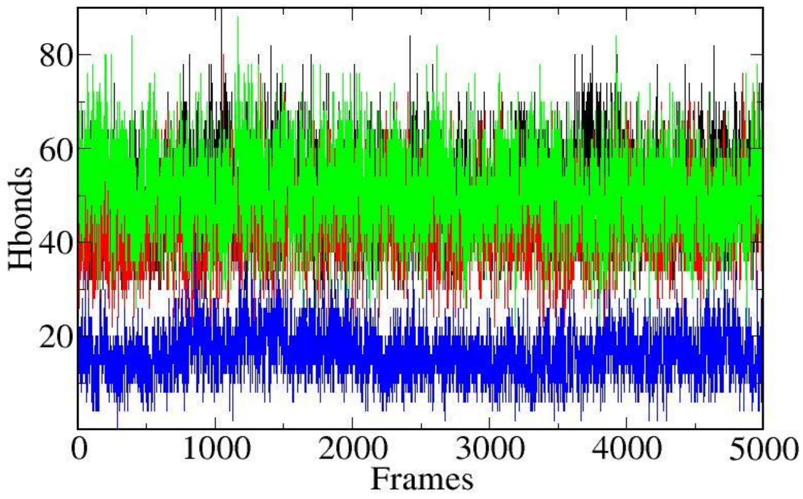

Figure S13: Radial Distribution Functions and hydrogen bonds.

NNNNNN-OH

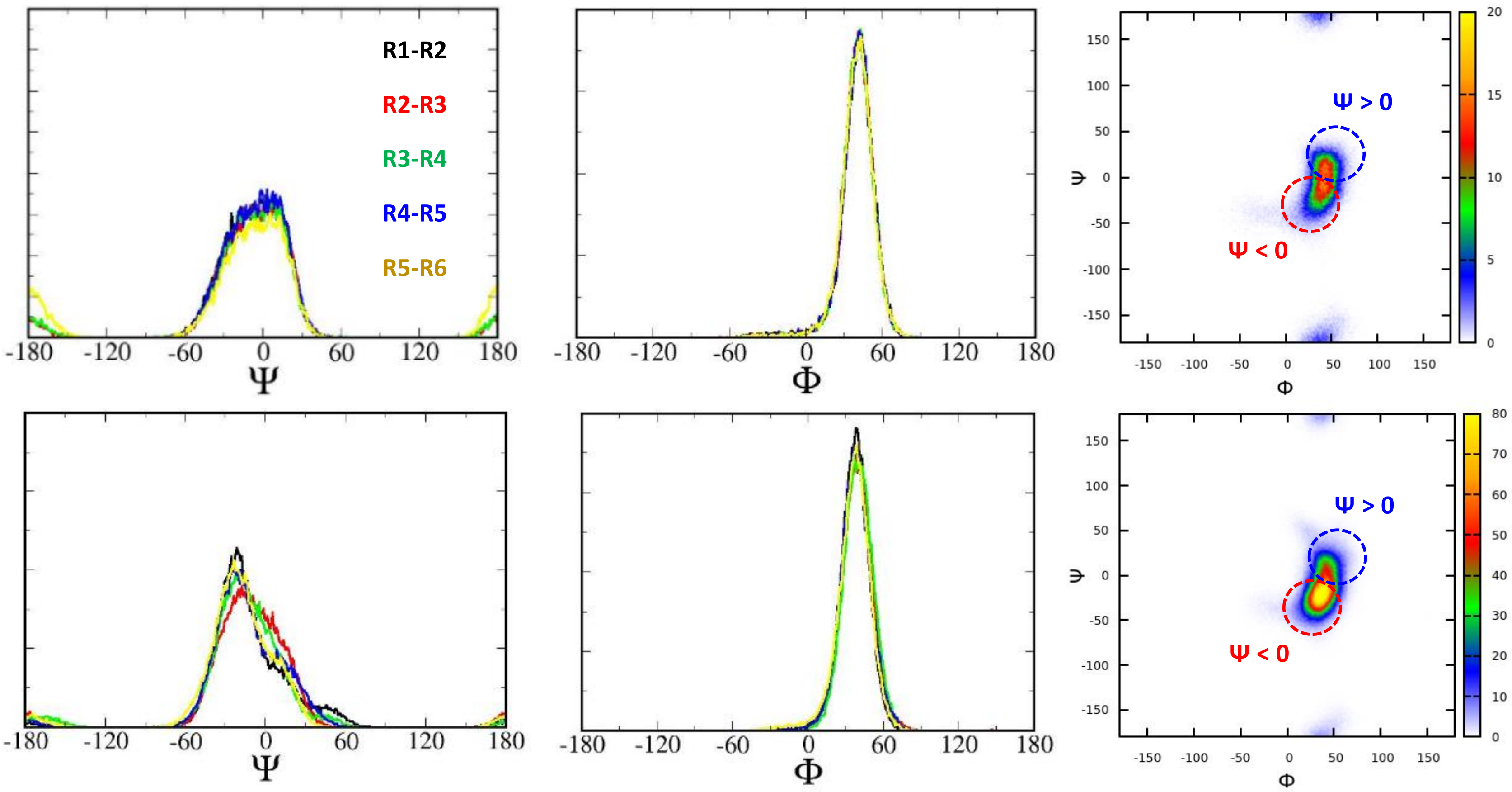

Figure S14a: Superimposed **ψ** and **ϕ** torsion angles for a single chain (*top*) *vs* the concentrated system (*bottom*).

NANNAN-OH

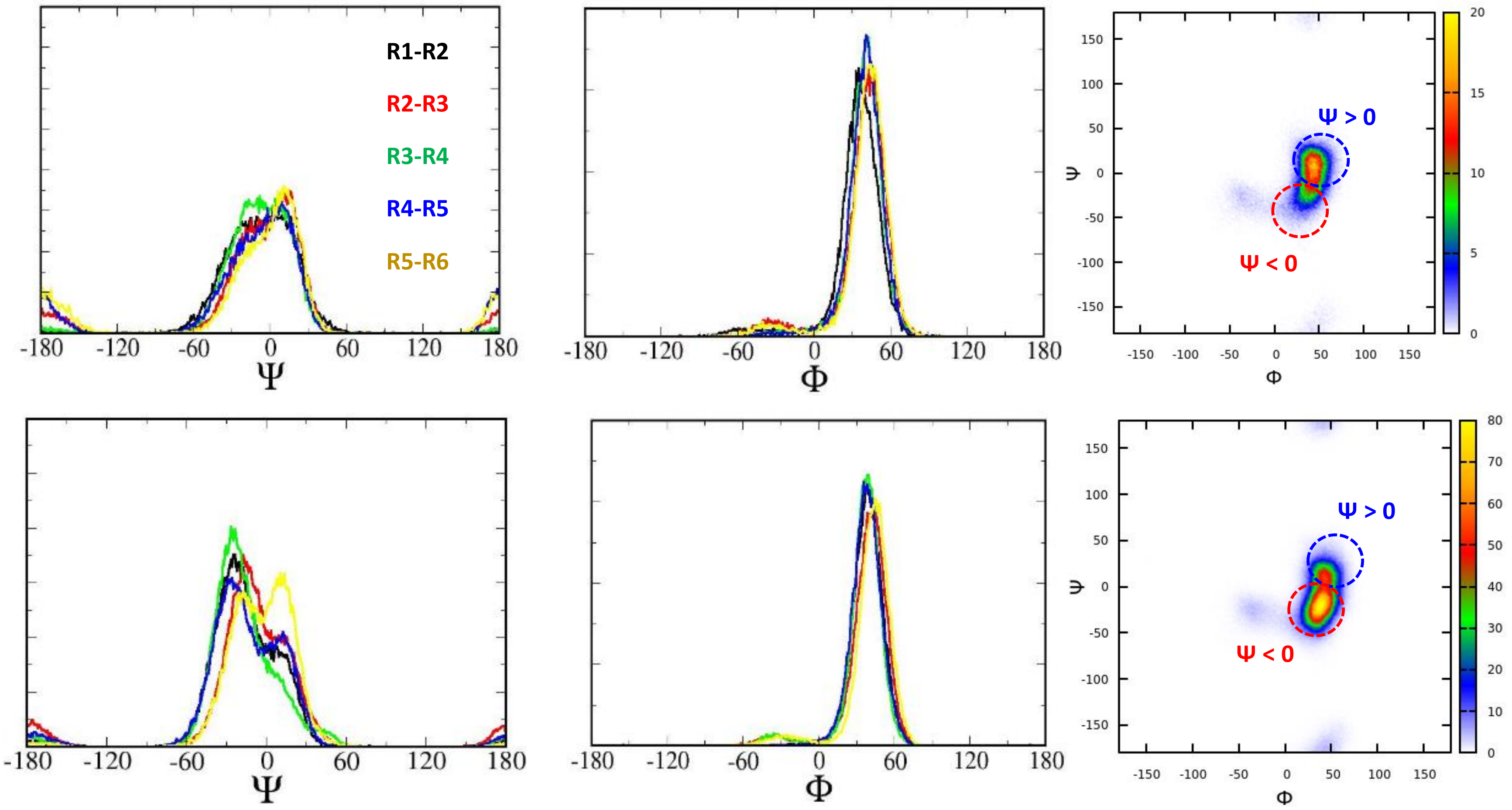

Figure S14b: Superimposed **ψ** and **ϕ** torsion angles for a single chain (*top*) *vs* the concentrated system (*bottom*).

$K^{+}NK^{+}NK^{+}N$-OH

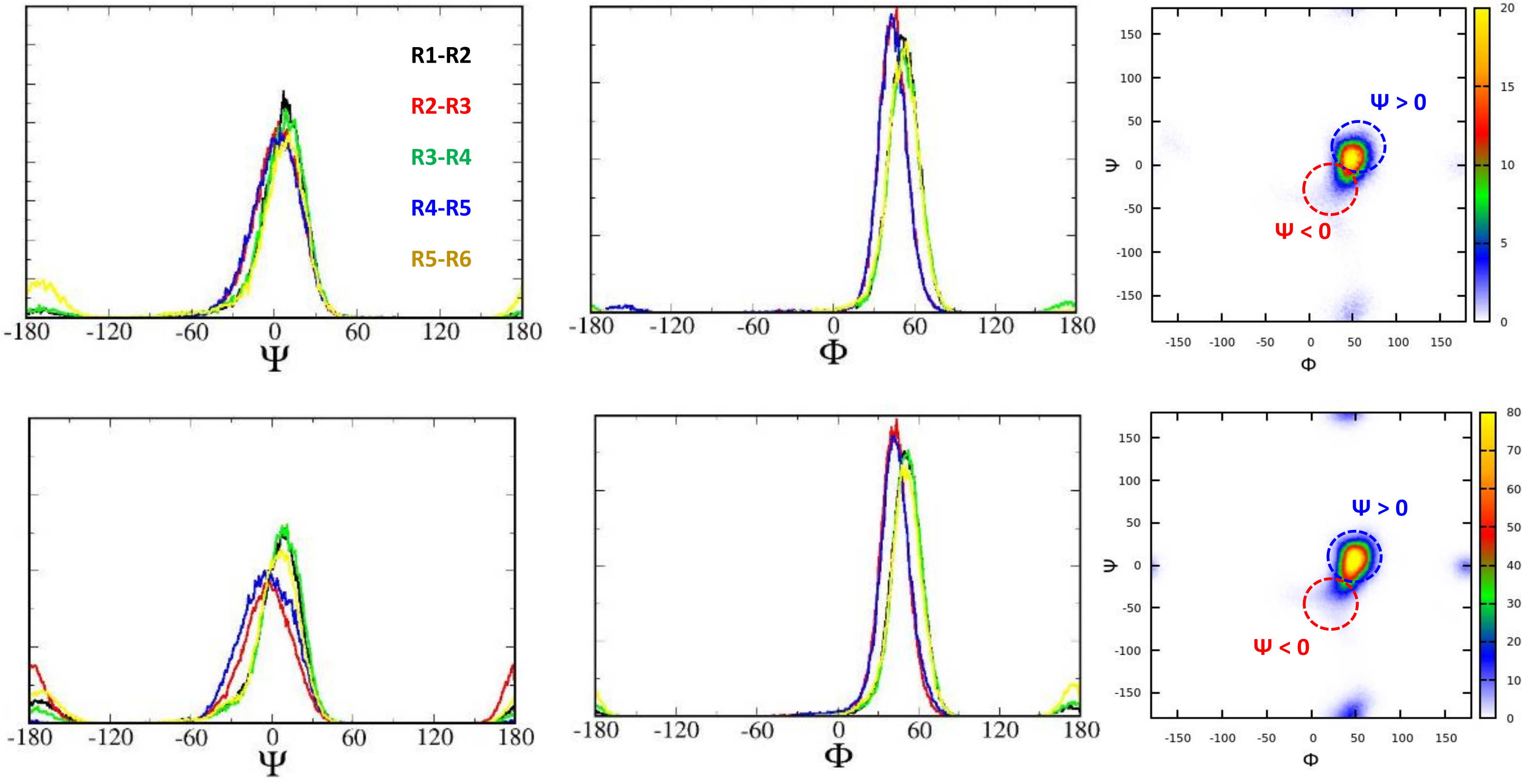

Figure S14c: Superimposed ψ and ϕ torsion angles for a single chain (*top*) *vs* the concentrated system (*bottom*).

NK$^+$NNK$^+$N-OH

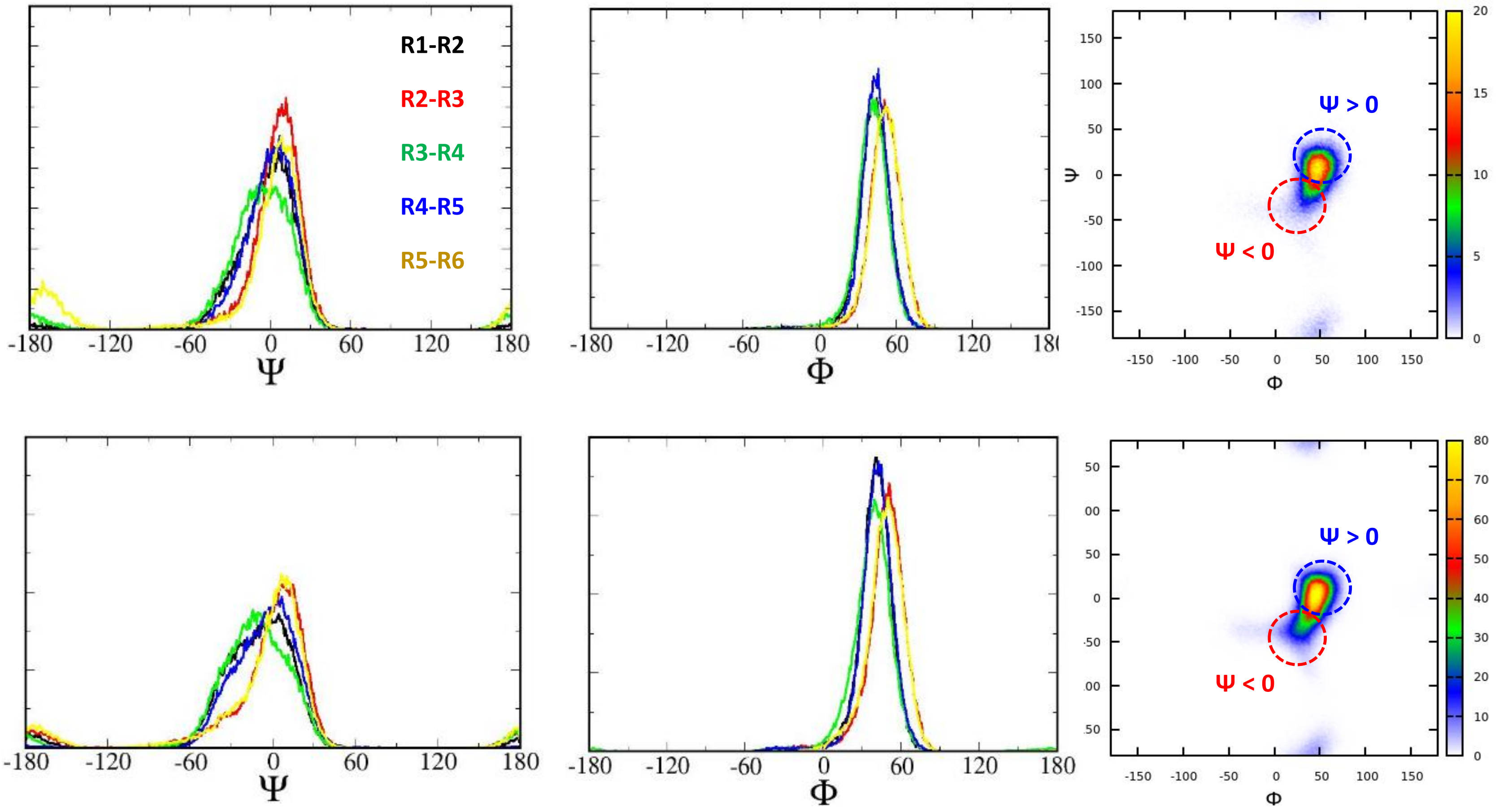

Figure S14d: Superimposed **ψ** and **ϕ** torsion angles for a single chain (*top*) *vs* the concentrated system (*bottom*).

NKNNKN-OH

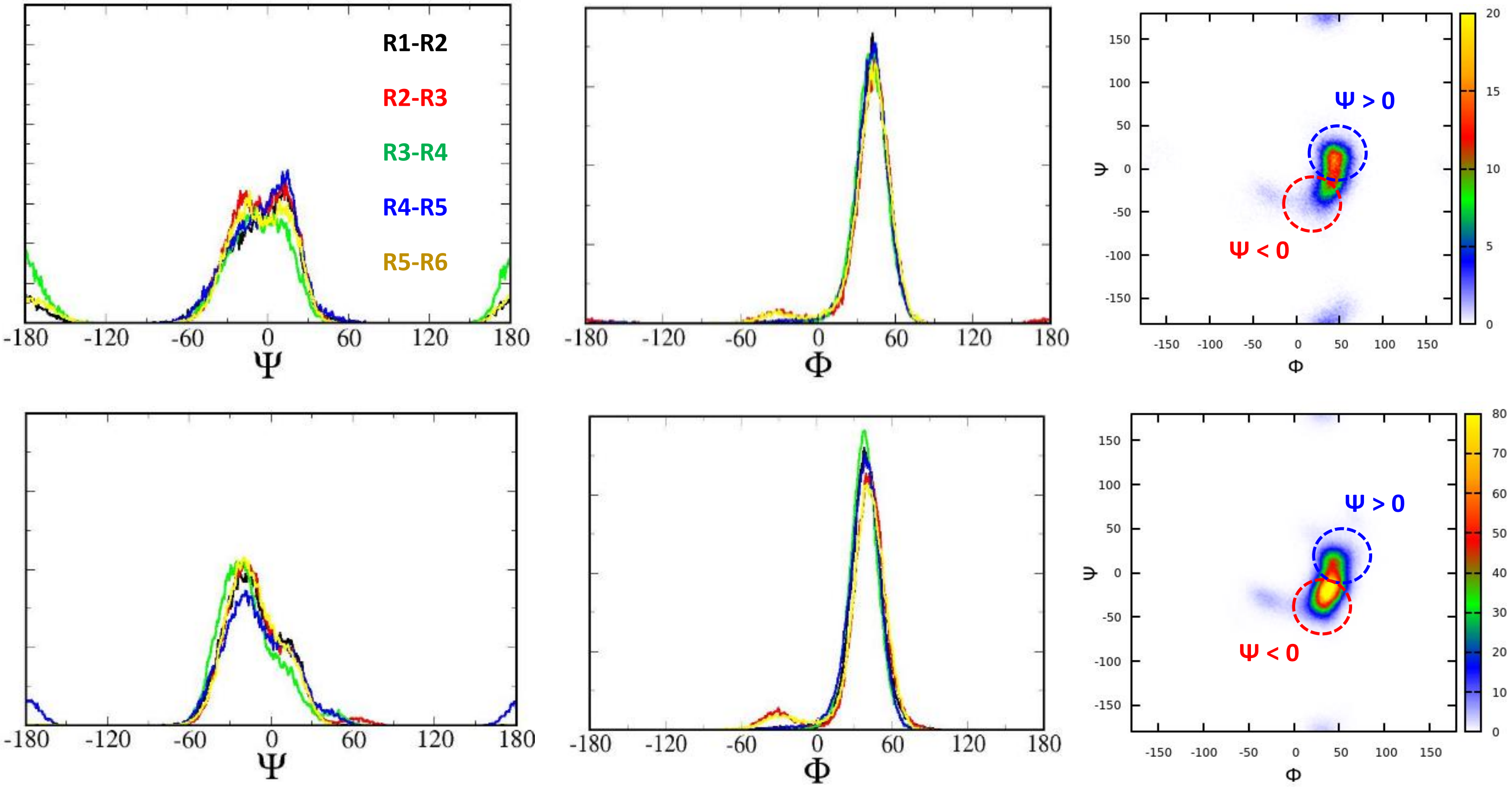

Figure S14e: Superimposed **ψ** and **ϕ** torsion angles for a single chain (*top*) *vs* the concentrated system (*bottom*).

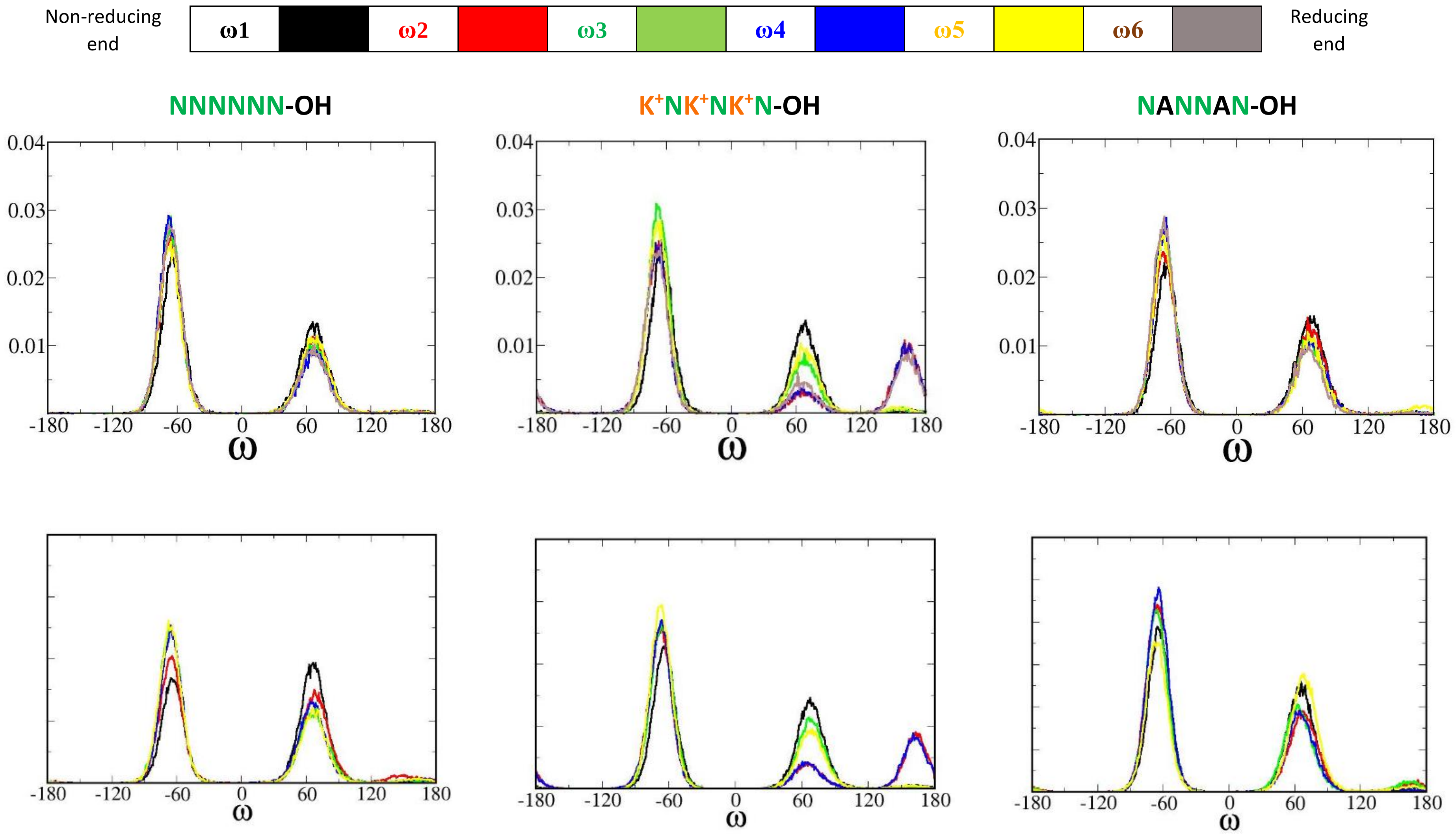

Figure S15: Superimposed Omegas (**ω**) torsion angles of N_6, **(K^+N)$_3$**, and **(NAN)$_2$** for a single chain (*top*) *vs* the concentrated system (*bottom*).

7. References

[1] a) K. Le Mai Hoang, A. Pardo-Vargas, Y. Zhu, Y. Yu, M. Loria, M. Delbianco, P. H. Seeberger, *J. Am. Chem. Soc.* **2019**, *141*, 9079-9086; b) L. Krock, D. Esposito, B. Castagner, C.-C. Wang, P. Bindschadler, P. H. Seeberger, *Chem. Sci.* **2012**, *3*, 1617-1622.

[2] M. Delbianco, A. Kononov, A. Poveda, Y. Yu, T. Diercks, J. Jiménez-Barbero, P. H. Seeberger, *J. Am. Chem. Soc.* **2018**, *140*, 5421-5426.

[3] R. Stenutz, I. Carmichael, G. Widmalm, A. S. Serianni, *J. Org. Chem.* **2002**, *67*, 949-958.

[4] Y. Yu, T. Tyrikos-Ergas, Y. Zhu, G. Fittolani, V. Bordoni, A. Singhal, R. J. Fair, A. Grafmüller, P. H. Seeberger, M. Delbianco, *Angew. Chem., Int. Ed.* **2019**, *58*, 1433-7851.

[5] M. Eddya, B. Tbib, K. El-Hami, *Heliyon* **2020**, *6*, e03486.

[6] D. van der Spoel, E. Lindahl, B. Hess, G. Groenhof, A. E. Mark, H. J. C. Berendsen, *J. Comput. Chem.* **2005**, *26*, 1701-1718.

[7] a) K. N. Kirschner, A. B. Yongye, S. M. Tschampel, J. González-Outeiriño, C. R. Daniels, B. L. Foley, R. J. Woods, *J. Comput. Chem.* **2008**, *29*, 622-655; b) J. r. Sauter, A. Grafmüller, *J. Chem. Theory Comput.* **2016**, *12*, 4375-4384.

[8] M. W. Mahoney, W. L. Jorgensen, *The Journal of Chemical Physics* **2000**, *112*, 8910-8922.

[9] a) W. G. Hoover, *Phys Rev A Gen Phys* **1985**, *31*, 1695–1697 ; b) S. Nosé, *J. Chem. Phys* **1984**, *81*, 511–519

[10] a) M. Parrinello, A. Rahman, *Physical Review Letters* **1980**, *45*, 1196-1199; b) M. Parrinello, A. Rahman, *Journal of Applied Physics* **1981**, *52*, 7182-7190.

[11] T. Darden, D. York, L. Pedersen, *Journal of Chemical Physics* **1993**, *98*, 10089-10092.

[12] B. Hess, H. Bekker, H. J. Berendsen, J. G. Fraaije, *J. Comput. Chem.* **1997**, *18*, 1463-1472.

[13] S. Miyamoto, P. A. Kollman, *J. Comput. Chem.* **1992**, *13*, 952-962.
